# Neosuberitenone, a New Sesterterpenoid Carbon Skeleton; New Suberitenones; and Bioactivity against Respiratory Syncytial Virus, from the Antarctic Sponge *Suberites* sp.

**DOI:** 10.3390/md21020107

**Published:** 2023-02-01

**Authors:** Joe Bracegirdle, Stine S. H. Olsen, Michael N. Teng, Kim C. Tran, Charles D. Amsler, James B. McClintock, Bill J. Baker

**Affiliations:** 1 Department of Chemistry, University of South Florida, 4202 E. Fowler Avenue, CHE205, Tampa, FL 33620, USA; 2 Department of Internal Medicine, University of South Florida, Tampa, FL 33612, USA; 3 Department of Biology, University of Alabama at Birmingham, 1300 University Blvd, Birmingham, AL 35233, USA

**Keywords:** antiviral, RSV, porifera, sesquiterpene, suberitane, ansellane

## Abstract

Respiratory syncytial virus (RSV) is a highly contagious human pathogen that poses a significant threat to children under the age of two, and there is a current need for new small molecule treatments. The Antarctic sponge *Suberites* sp. is a known source of sesterterpenes, and following an NMR-guided fractionation procedure, it was found to produce several previously unreported metabolites. Neosuberitenone (**1**), with a new carbon scaffold herein termed the ‘neosuberitane’ backbone, six suberitenone derivatives (**2**–**7**), an ansellane-type terpenoid (**8**), and a highly degraded sesterterpene (**9**), as well as previously reported suberitenones A (**10**) and B (**11**), were characterized. The structures of all of the isolated metabolites including absolute configurations are proposed on the basis of NMR, HRESIMS, optical rotation, and XRD data. The biological activities of the metabolites were evaluated in a range of infectious disease assays. Suberitenones A, B, and F (**3**) were found to be active against RSV, though, along with other *Suberites* sp. metabolites, they were inactive in bacterial and fungal screens. None of the metabolites were cytotoxic for J774 macrophages or A549 adenocarcinoma cells. The selectivity of suberitenones A, B, and F for RSV among other infectious agents is noteworthy.

## 1. Introduction

With the recent emergence of SARS-CoV-2, widespread attention has been drawn to the severity of respiratory illness caused by viral infections. Aside from this and well-known influenza, respiratory syncytial virus (RSV) is another major global pathogen, responsible for bronchiolitis and pneumonia in infants and toddlers under the age of two, as well as severe, sometimes deadly, pneumonia, chronic obstructive pulmonary disease, and asthma in elderly adults [1,2]. Currently there are only two FDA-approved drugs for the treatment of RSV, the guanosine analogue ribavirin and the monoclonal antibody palivizumab [3]; however, both have significant drawbacks. The use of ribavirin is limited to RSV infections in immunocompromised patients owing to its nonspecific activity and toxicity, in conjunction with its relatively high cost [4], while palivizumab is only recommended for prophylactic use in high-risk infants and children [5]. Therefore, there is a current need for new effective and affordable treatments for the widespread RSV pathogen.

**Figure 1:**
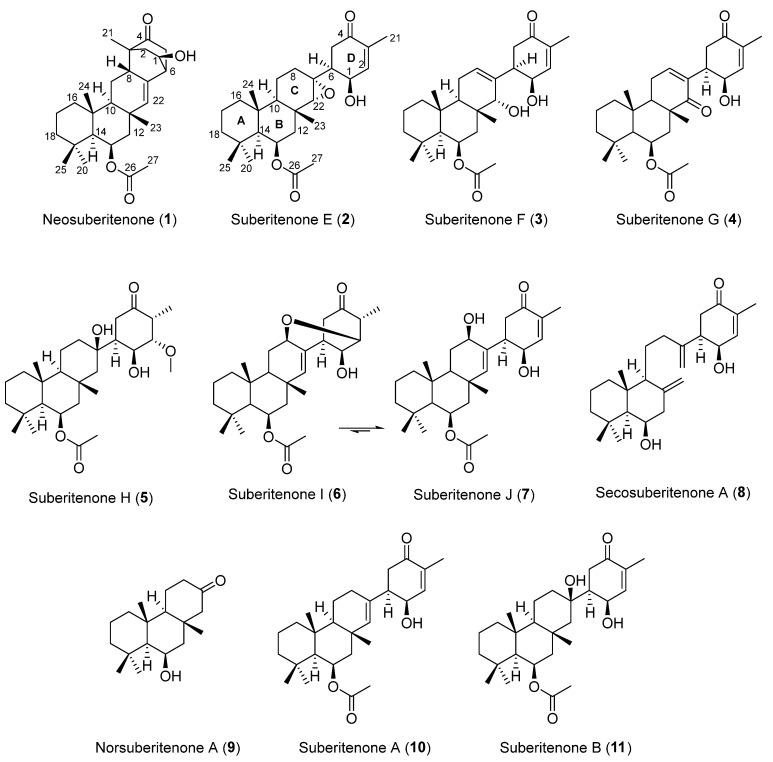
Terpenoids found in *Suberites* sp. from Palmer Station, Antarctica.

Marine sponges collected in the cold waters of Antarctica continue to be a fruitful source of novel bioactive metabolites [6,7,8]. The suberitenones are a class of oxidized sesterterpenes of the ‘suberitane’ carbon skeleton and have been reported from multiple Antarctic *Suberites* sp. samples as well as *Phorbus areolatus* collected in the same waters [9,10,11,12]. More recently, anvilone A and B, two metabolites that also share the same carbon skeleton backbone, were reported from a *Phorbus* sp. sample collected in the temperate waters of Anvil Island, British Columbia [13]. These metabolites have displayed a range of bioactivities, with oxaspirosuberitenone demonstrating mild antibacterial activity against MRSA, isosuberitenone B and 19-episuberitenone B showing weak cytotoxicity against a panel of tumor cell lines, and anvilone A turned on HIV gene expression, showcasing the potential of this class of metabolites for biomedical applications.

As part of our ongoing investigation into the chemistry of Antarctic marine organisms, a *Suberites* sp. sample collected in 2018 was subjected to a ^1^H NMR-guided purification procedure using reversed-phase HPLC. The previously reported compounds suberitenones A (**10**) and B (**11**) (Figure 1) were both isolated in large quantities from the least polar fractions, while the ^1^H NMR spectra of earlier eluting fractions showed a number of interesting resonances warranting further analysis. This led to the isolation of nine previously unreported sesterterpene compounds, **1** to **9**, of which **1** was made up of a previously unreported carbon skeleton and **6** existed as an interconverting equilibrium between two structures, **6** and **7**. Structure elucidation of the isolated compounds and an assessment of their RSV antiviral activity are reported herein. 

## 2. Results and Discussion

Neosuberitenone A (**1**) was isolated as white crystals, with a molecular formula C_27_H_40_O_4_ (eight double bond equivalents) established by analysis of the (−)-HRESIMS formate adduct ion at *m*/*z* 473.2915. The 1D ^13^C NMR spectrum (125 MHz, CDCl_3_) gave signals for all 27 carbons (Table 1) and in conjunction with the multiplicity-edited HSQC data, it suggested that **1** contained a ketone, a carboxylate, four sp^3^ and one sp^2^ quaternary carbons, seven methine carbons of which one is olefinic and two are oxygen-bearing, seven methylenes, and six methyl groups. These data account for three degrees of unsaturation, therefore **1** must also contain five rings. The ^1^H NMR data (Table 2) showed signals of two oxygenated methines at δ_H_ 4.18 (dd, *J* = 3.5, 8.7 Hz) and δ_H_ 5.51 (br q, *J* = 3.9 Hz) and an olefin at δ_H_ 5.60 (d, *J* = 3.1 Hz), and it was also observed that the five aliphatic and acetate methyl groups were all singlets, while the remainder of the 1D ^1^H NMR spectrum was complicated by overlapping aliphatic signals.

**Figure 2:**
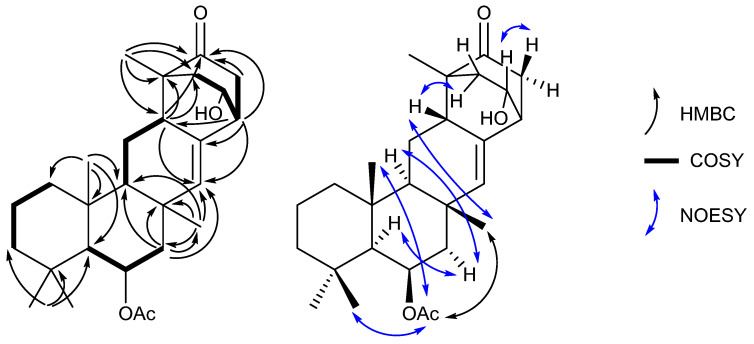
Key 2D NMR correlations establishing the structure of neosuberitenone A (**1**).

These data, along with correlations in the COSY and HMBC spectra (Table S1), suggested the structure of neosuberitenone A (**1**) to be closely related to those of the suberitane class of sesterterpenoids (Figure 2). The first spin system was deduced from COSY correlations from H_2_-16 (δ_H_ 0.82 and 1.55) to H_2_-17 (δ_H_ 1.41 and 1.66) and then H_2_-18 (δ_H_ 1.14 and 1.35), which comprise the backbone of ring A. The ring assignment was completed using HMBC correlations from the geminal dimethyl H_3_-20 and H_3_-25 (δ_H_ 0.92 and 1.01, respectively) to C-14, C-18, and C-19 (δ_C_ 57.1, 44.2 and 34.0, respectively), in conjunction with methyl H_3_-24 (δ_H_ 1.28) to C-10, C-15, C-16 (δ_C_ 55.1, 38.2 and 41.7, respectively), and C-14. Next, the cycle of ring B was assigned using COSY correlations between H-14 (δ_H_ 1.04) and oxygenated H-13 (δ_H_ 5.51) and then onto methylene H_2_-12 (δ_H_ 2.09 and 1.44), and HMBC correlations from H_2_-12 to C-11, C-22, C-23 (δ_C_ 35.7, 140.0 and 19.6, respectively), and C-10. Methyl group H_3_-23 (δ_H_ 1.18) showed HMBC correlations to C-10, C-11, C-12, and C-22 so it was therefore placed on quaternary C-12. Ring C, however, showed differences from the suberitane class, lacking the C-8 methylene which was instead replaced by a methine (δ_H_ 2.68) that was assigned using COSY correlations to H_2_-9 (δ_H_ 1.75 and 1.26) and H-10 (δ_H_ 1.02) and HMBC correlation to olefin C-22.

The remainder of neosuberitenone A (**1**) consisted of one major spin system between H_2_-2 (δ_H_ 2.13 and 1.64), oxygenated H-1 (δ_H_ 4.18), H-6 (δ_H_ 2.69), and H_2_-5 (δ_H_ 2.25 and 2.10) deduced from COSY correlations. The HMBC spectrum showed correlations from H-8 to C-2, C-3, and C-4 (δ_C_ 42.9, 48.7, and 214.3, respectively) which connected the two units, while the methyl H_3_-21 (δ_H_ 1.01) was placed at C-3 as it showed HMBC correlations to C-2, C-3, C-4, and C-8. This spin system was reasoned to be cyclized between C-4 and C-5 (δ_C_ 41.9) as both H_2_-5 and H-6 showed HMBC correlations to ketone C-4. HMBC correlations from H-6 were able to finalize the ring structure of **1**, with correlations to C-7, C-8 (δ_C_ 134.3 and 40.8, respectively), and C-22 providing evidence for a bond between C-6 and C-7. The planar structure of **1** was completed by placing the acetyl group on the oxygen of C-13, based on correlations in the NOESY spectrum between H_3_-27 (2.06) and H_3_-23, H_3_-24, and H_3_-25 which established that the acetyl group was not on the C-1 oxygen.

The stereochemistry of neosuberitenone A (**1**) was deduced by correlations in the NOESY spectrum. The similar elements of **1** and suberitenone A (**10**) were determined to have the same stereochemistry, with methyl groups H_3_-23, H_3_-24, and H_3_-25 all deduced to be cofacial with acetyl group H_3_-27 by NOESY correlations, while correlations between H-12′ to both H-10 and H-14 also placed these protons on the opposite face of the molecule. A correlation between H_3_-23 to H-8 set the orientation of this center relative to the methyl groups deduced previously, and the correlation from H-8 and H-2′ also suggested these two protons to be *syn*. As C-3 and C-6 are joined by a two-carbon bridge, this also set the stereochemistry at C-6 as the bridging carbons must be on the same side of the molecule. Finally, correlations between H-1 and H-5 suggested the H-1 proton to be oriented toward C-5 and thus **1** was deemed to have the 1R*,3R*,6R*,8S*,10S*,11S*,13R*,14S*,15R* configuration.

**Figure 3:**
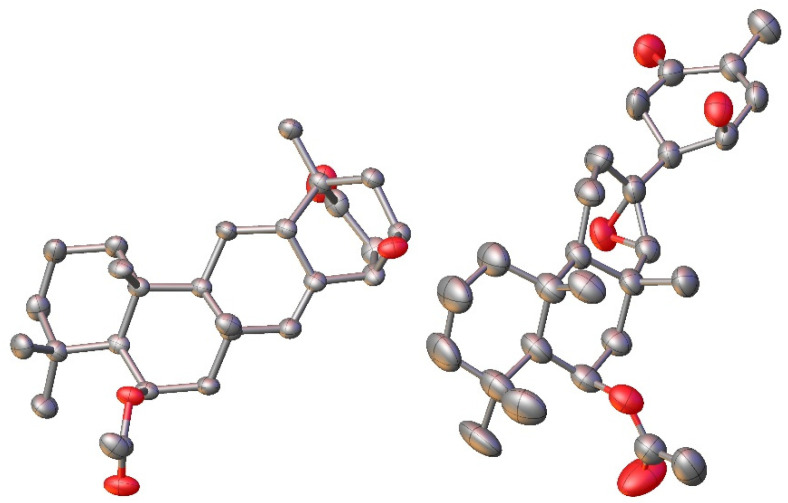
X-ray crystal structure for **1** (**left**) and **2** (**right**). Hydrogens omitted for clarity.

This spectroscopic data analysis was confirmed by single-crystal X-ray diffraction (XRD) studies, with neosuberitenone A (**1**) forming suitable crystals from 9:1 ACN:H_2_O. The absolute configuration of **1** was determined to be 1*R*,3*R*,6*R*,8*S*,10*S*,11*S*,13*R*,14*S*,15*R* (Figure 3), which is similar to that deduced by Shin and coworkers on suberitenone A (**10**) and B (**11**) using modified Mosher’s method and CD analysis [9]. This terpenoid carbon backbone of **1** is an unprecedented class to date and is termed the ‘neosuberitane’ backbone.

Suberitenone E (**2**) was also isolated as white crystals, with a molecular formula C_27_H_40_O_5_ established by analysis of the formate adduct ion at *m*/*z* 489.2865 in the (−)-HRESIMS data. Analysis of the 1D and 2D NMR data (Table S2) suggested **2** to be structurally related to suberitenone A (**10**), with major differences present in ring C, notably the absence of the olefin and introduction of a new singlet for H-22 (δ_H_ 2.63; δ_C_ 70.1). A comparison of the molecular formulas shows **2** to have one extra oxygen atom, all of which points to the presence of an epoxide across the C-7/C-22 bond. Correlations in the NOESY spectrum along with coupling constant analysis were used to deduce that all of the stereocenters shared with suberitenone A were of the same orientation, with a correlation between epoxide H-22 and H_3_-23 (δ_H_ 1.27) suggesting a *syn* relationship and therefore establishing the configuration of both C-7 and C-22. Single-crystal XRD confirmed this interpretation and established the absolute configuration as 1*R*,6*S*,7*R*,10*R*,11*R*,13*R*,14*S*,15*R*,22*R,* as in Figure 3.

**Figure 4:**
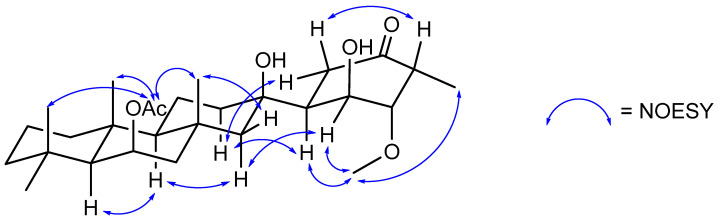
Key NOESY correlations establishing the relative stereochemistry of suberitenone H (**5**).

Suberitenone F (**3**) was isolated as a white film, with a molecular formula C_27_H_40_O_5_ established by analysis of the formate adduct ion at *m*/*z* 489.2841 in the (−)-HRESIMS data and is thus isomeric with suberitenone E (**2**). The major noticeable differences in the NMR data are related to the resonances of ring C, markedly the presence of an olefinic ^1^H resonance (δ_H_ 5.67) that showed COSY correlations to H_2_-9 (δ_H_ 2.12). The 2D NMR correlations (Table S3) inferred this signal to be from H-8 and thus suggest a C-7/C-8 double bond, as found in that of suberitenone C (**12**, Figure 4). Similar to **2**, the NMR data gave evidence for an oxymethine at C-22 (δ_H_ 3.15; δ_C_ 75.3) and therefore placed a hydroxyl group at this center, completing the planar structure of **3**. Correlations in the NOESY spectrum were used to deduce the chiral centers, where the key correlation between H-22 and H_3_-23 (δ_H_ 0.99) showed these two to be related *syn* to each other, leading to the 22*R* assignment. All other observed correlations confirmed the remaining configurations to be the same as those deduced by XRD on **2**.

Suberitenone G (**4**) was isolated as a white film, with a molecular formula C_27_H_38_O_5_ established by analysis of the deprotonated molecule at *m*/*z* 441.2645 in the (−)-HRESIMS data. The NMR data (in MeOD as decomposition was observed in CDCl_3_, Table S4) for **4** suggested the molecule to be related to suberitenone A (**10**); however, the C ring olefin resonances were further deshielded moving from δ_H_ 5.18; δ_C_ 138.9 to δ_H_ 6.78; δ_C_ 147.2. Combined with the observation of a second α,β-unsaturated ketone (δ_C_ 205.5) that showed HMBC correlations from this new olefin methine, it was suggested that **4** contained a enone in ring C as well as ring D. A HMBC correlation from H_3_-23 (δ_H_ 1.27) to the new ketone placed it as C-22, while COSY correlations between H-10 (δ_H_ 1.71) and H_2_-9 (δ_H_ 2.52) to olefin H-8 (δ_H_ 6.78) confirmed the ring C enone orientation. The configuration of the stereocenters of **4** was deduced from NOESY correlations to be the same as that of **2,** as was previously deduced by XRD.

Suberitenone H (**5**) was isolated as a white film, with a molecular formula C_28_H_46_O_6_ established by analysis of the formate adduct ion at *m*/*z* 523.3284 in the (−)-HRESIMS data. Analysis of the ^1^H and ^13^C NMR data suggested **5** to be similar to suberitenone B (**11**), hydrated across the C-7/C-22 bond with tertiary alcohol at C-7. However, **5** exhibited several differences in the resonances associated with ring D including the loss of the olefinic ^13^C/^1^H signals, splitting of the methyl signal into a doublet, and the gain of an aliphatic methine, an oxymethine, and a methoxy signal. The doublet methyl H_3_-21 (δ_H_ 1.02) correlated in the COSY spectrum to H-3 (δ_H_ 3.03) which correlated with H-2 (δ_H_ 3.52). The signal of H-2 and methoxy H_3_-28 showed mutual HMBC to the other carbons for C-28 and C-2 (δ_C_ 59.1 and 88.6, respectively) and this accounts for the loss of the C-2/C-3 double bond. The remainder of ring D remains the same as suberitenone B, assigned on the basis of COSY and HMBC correlations (Table S5).

The stereochemistry of suberitenone H (**5**) was deduced by a combination of *J* coupling constant analysis and correlations in the NOESY spectrum. The similar elements of **5** and suberitenone B (**11**) were determined to have the same configurations as above, with methyl groups H_3_-23 (δ_H_ 1.37), H_3_-24 (δ_H_ 1.23), and H_3_-25 (δ_H_ 1.04) all deduced to be cofacial with acetyl group H_3_-27 (δ_H_ 2.03) by NOESY correlations, while a correlation between H-22 (δ_H_ 1.81) and H_3_-23 also placed this proton *syn*. In contrast, H-22′ (δ_H_ 1.15) showed a NOESY correlation to H-10 (δ_H_ 0.99) which shared a correlation with H-14 (δ_H_ 1.14), placing these two on the opposite side of the molecule. With the large coupling constant (*J* = 13.7) between H-5 (δ_H_ 2.65) and H-6 (δ_H_ 1.81), these protons share a pseudo-axial–axial relationship, and as has been seen for previously isolated suberitenones [12], this suggested C-6 (δ_C_ 48.3) to have an *S* configuration. The relatively smaller coupling constant between H-6 and H-1 (*J* = 3.3) suggested C-1 (δ_H_ 66.6) to also have the *S* configuration. The cofacial relationship of these two protons is further affirmed by both showing NOESY correlations with methoxy H_3_-28, which also correlated to H_3_-21, suggesting that this group was on the same face. In addition, a NOESY correlation between pseudo-axial H-3 and H-5 provided the final evidence to assign 2*S* and 3*R* to the ring. The last chiral center, C-7, is a tertiary alcohol and was assigned an *R* configuration, the same as that for suberitenone B. The chemical shift is consistent with the previously isolated molecules, while the NOESY correlations between H-1 and H-22′ and H-8′ with both H-5′ and H-6 mirror those used diagnostically for isosuberitenone B [12].

Suberitenone I (**6**) was isolated as a white film with a molecular formula C_27_H_40_O_5_ established by analysis of the formate adduct ion at *m*/*z* 523.3284 in the (−)-HRESIMS data, therefore being isomeric with **2**. Like suberitenone H (**5**), the ^1^H and ^13^C NMR data showed no evidence of a ring D double bond, and instead, the signal for H_3_-21 (δ_H_ 1.19) was a doublet, and the chemical shift of C-2 and H-2 (δ_C_ 79.9, δ_H_ 4.03) suggested the center was oxygenated (Table S6). Based on COSY correlations throughout the spin system with H-10 (δ_H_ 1.13) and H_2_-9 (δ_H_ 1.90 and 1.67), H-8 was assigned (δ_H_ 3.79, δ_C_ 70.8) and both the ^13^C and ^1^H chemical shifts suggest the center was also oxygen-bearing. The planar structure of **6** was finally deduced by a key HMBC correlation from H-2 to C-8, thus providing evidence for an oxa-bicyclo[3.3.1]nonane moiety and providing the correct number of degrees of unsaturation.

While NMR data were collected on purified suberitenone I (**6**), the signals decreased in intensity while those of a new species grew in intensity. This more-polar compound was separable by reversed-phase HPLC (Figure S44), and from analysis of the signals, it was clear the new species was a suberitenone-type molecule, with the same enone D ring as suberitenone A (**10**), and COSY/HSQC correlations revealed C-8 (δ_H_ 4.30) to be hydroxylated (Table S7). Thus, suberitenone J (**7**) is the oxaspiro-ring-opened isomer, existing in equilibrium with **6**. When the two were separated by reversed-phase HPLC, both reverted to an equilibrium mixture of ~3:1 **6**:**7** over 24 h.

To determine the configuration of the chiral centers, key NOESY correlations and the ^1^H NMR coupling constants for both compounds were taken into account. In the NOESY spectra of both compounds, a key correlation between H-8 and H-10 placed these two protons *syn*, establishing the orientation of C-8 relative to the suberitane scaffold. As with oxaspirosuberitenone, the extra ring distorts the D ring away from a pseudo-chair conformation in suberitenone I (**6**) and thus, coupling constants cannot be understood to rapidly assign configurations. This is not the case for suberitenone J (**7**) where the pseudo-axial–axial coupling constant of H-5 and H-6 (*J* = 13.7 Hz) established that C-6 shares the same configuration as the previously isolated congeners and the same can be applied between H-6 and H-1 (*J* = 4.3 Hz) to establish C-1. Finally, correlations to H_3_-21 from H-2 were used to assign the orientation of the methyl group on C-3, which is in accordance with the other molecules isolated from this organism.

**Figure 5:**
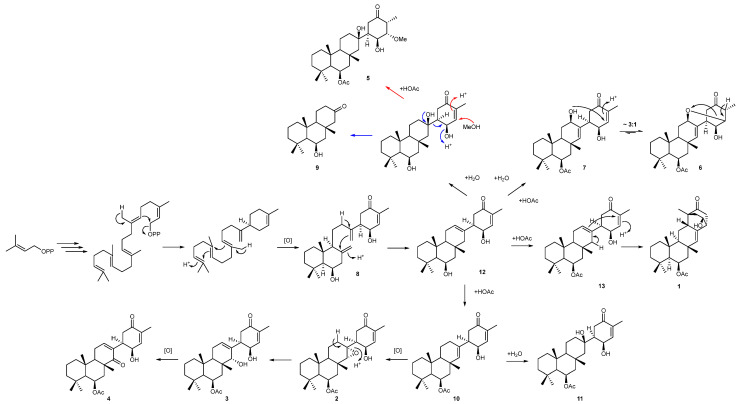
Proposed biogenesis of suberitane and neosuberitane backbone based on the compounds isolated in this study. Blue arrows indicate the degradation path to **9** while red arrows indicate the methanol addition pathway leading to **5**.

Secosuberitenone A (**8**) was isolated as a white film, with a molecular formula of C_25_H_38_O_3_ established by analysis of the formate adduct ion at *m*/*z* 431.2814 in the (−)-HRESIMS data. The 1D and 2D NMR data suggested major differences present in ring C and also lacked the signals of the acetyl group which suggested that the ring B hydroxyl did not bear an acetyl group as in the previously isolated metabolites (Table S8). The most significant difference with other suberitenones was the presence of two terminal vinylidene moieties, thereby accounting for all seven degrees of unsaturation and therefore suggesting that ring C was not cyclized. The ^1^H signals for H_2_-23 (δ_H_ 5.03 and 4.77) showed HMBC correlations to C-10, C-11, and C-12 (δ_C_ 57.5, 144.4, and 47.8, respectively); therefore, this replaced the methyl group normally present there, whereas vinylidene H_2_-22 (δ_H_ 5.16 and 4.92) showed correlations to C-1, C-6, C-7, and C-8 (δ_C_ 63.6, 45.5, 148.8, and 34.2, respectively) which revealed it to be adjacent to ring D. Both sets of correlations lead to the conclusion that compound **8** lacks the cyclization bond from C-10 to C-22 typically formed in the terpene backbone cyclization to form the suberitane scaffold (Figure 5), instead forming the ansellane backbone first observed in ansellone A [14]. The configuration of chiral centers in **8** were deduced from NOESY correlations to be the same as that of **2** as was previously deduced by XRD. 

Norsuberitenone A (**9**) was isolated as a white crystalline solid with the molecular formula C_18_H_30_O_2_ deduced from analysis of the protonated molecular at *m*/*z* 279.2320 (+)-HRESIMS data. Norsuberitenone A is significantly smaller than previously isolated suberitenones, and the NMR spectra lacked signals associated with the D ring or the acetyl group. Using HMBC correlations (Table S9) from H_3_-15, H_3_-17, and H_3_-18, rings A and B were deduced to be the same as the suberitenone A (with a hydroxyl in place of the acetyl group at C-8); however, ring C was determined to have a ketone at C-2 (δ_C_ 215.1) based on HMBC correlations from H_2_-1 (δ_H_ 2.21 and 1.90), H_2_-3 (δ_H_ 2.37 and 2.33), and H-4 (δ_H_ 2.05). This position is the tertiary alcohol carbon that bridges to ring D in suberitenone B (**11**); therefore, it is likely that **9** forms as a degradation product from this (blue arrows, Figure 5). The stereochemistry of the chiral centers was assigned the same configuration as the other congeners based on correlations in the NOESY spectrum.

A proposed biogenesis of newly isolated metabolites **1** to **11** is presented in Figure 5. Both **1** and **4** can be envisaged to form from common intermediate **12** (desacetoxy suberitenone C = suberitenol C) [10] by either an intramolecular cyclization or oxidation reaction, respectively. Compound **2** forms from suberitenone A (**10**) by a further epoxidation reaction of the C7/C22 double bond, while **5** is likely to be a methanol addition product of suberitenone B (**11**). While we cannot rule out **5** as an artifact of isolation, it would be unusual that only suberitenone B, and none of the other six α,β-unsaturated ketones, added methanol. Furthermore, stirring pure isolated suberitenone B in methanol for one week did not produce any detectable amount of **5**. Metabolite **9** bearing only 18 carbon atoms is likely to be a ‘heptanorsesquiterpene’ formed by the elimination of the suberitenone B D ring. The purification procedure used avoided the use of ethyl acetate, which proved the acetyl group at C-13 to be biogenerated.

The newly isolated compounds alongside suberitenones A (**10**) and B (**11**) were assessed for their ability to inhibit RSV gene expression and their effect on the cell viability (Table 3). Neosuberitenone (**1**) was the only compound that did not show any viral inhibitory effects at 25 µg/mL (**6**, **8,** and **9** were not tested due to a lack of sample supply), suggesting that the ring D must be appended by a single bond to result in activity. Suberitenones A and B demonstrated the strongest antiviral potential of the tested compounds, with IC_50_ values of 7.9 and 3.5 µM, respectively, while suberitenone F (**3**) was also active (IC_50_ = 9.8 µM). The other isolated compounds had IC_50_ values ranging from 10.9–20.5 µM (Table S12), therefore suggesting that oxidation about ring C and removing the enone double bond of ring D are not likely to be alterations that would result in an increase in activity for future structure–activity relationship studies. The CC_50_ values were ~4–5 fold higher than the IC_50_ values for the newly isolated compounds (Table 3). Suberitenone B (**11**) showed the highest activity and greatest selectivity index.

*Suberites* sp. compounds were also submitted to a broad range of infectious disease screening. No antibacterial activity was detected against the ESKAPE pathogens, nor antifungal activity against several *Candida* strains. The cytotoxicity was assayed against the murine macrophage J774 cell line, where no significant activity (EC_50_ below 10 µM) was observed for any compound. Taken with low cytotoxicity, the selectivity of suberitenones A and B (**10**, **11**) for RSV among other infectious diseases is noteworthy.

## 3. Materials and Methods

### 3.1. General Experimental Procedures

Optical rotations were measured using an AutoPol IV polarimeter at 589 nm. UV/Vis spectra were extracted from HPLC chromatograms. NMR spectra were acquired using either a Varian Inova 500 spectrophotometer or a Varian 600 MHz broadband spectrophotometer. The residual solvent peak was used as an internal chemical shift reference (CD_3_OD: δ_C_ 49.0; δ_H_ 3.31, CDCl_3_: δ_C_ 77.0; δ_H_ 7.26). High-resolution mass spectrometry−liquid chromatography data were obtained on an Agilent 6540 QTOF LCMS with electrospray ionization detection. Reversed-phase HPLC was performed on a Shimadzu LC20-AT system equipped with a photodiode array detector (M20A) using a preparative Phenominex C18 column (5 μm, 100 Å, 250 × 21.2 mm; 9 mL/min) or on a semipreparative Phenominex C18 column (10 μm, 100 Å, 250 × 10 mm; 4 mL/min). All of the solvents used for column chromatography were of HPLC grade, and H_2_O was distilled. Solvent mixtures are reported as % *v*/*v* unless otherwise stated.

### 3.2. Biological Material, Extraction and Isolation

Sponge specimens were collected in the austral fall, 2018, within a 3.5 km radius of Palmer Station, Antarctica (64° 46.50’ S; 64° 03.30’ W). The frozen sponge was freeze-dried (1.35 kg wet weight and 200 g dry weight) and then crushed by hand before being extracted in MeOH (1.6 L) twice overnight. The extracts were combined, were then passed through an HP20 column (250 mL), pre-equilibrated in H_2_O, and combined following elution. The eluent was then diluted with an equal volume of H_2_O and passed back through the column twice, followed by a 750 mL H_2_O wash. The column was then eluted with 750 mL portions of (1) 75% Me_2_CO/H_2_O and (2) Me_2_CO (fractions A1 and A2, respectively). Fraction A1 (5 g) was then reconstituted in MeOH (25 mL), filtered, and fractionated generating fractions (B1–B25) by repeated preparative C18 HPLC (9 mL/min) using the following method: 70% can/H_2_O (0.1% CH_2_O_2_) for 6 min, a linear gradient to 100% ACN over 8 min, and 100% ACN isocratic for 11 mins. Fraction B25 contained suberitenone A (~500 mg), while fraction B23 contained suberitenone B (~250 mg). Fraction B20 was purified using semipreparative C18 HPLC using a linear gradient from 50% MeOH/H_2_O (0.1% CH_2_O_2_) to 100% MeOH over 25 min to afford **1** (5.6 mg) and **2** (1.6 mg), while fractions B15, B16, B17, B18, B19, and B21 were also purified by the same method to afford **6** (0.8 mg), **4** (3.4 mg), **8** (0.8 mg) **3** (1.9 mg), **9** (0.8 mg), and **5** (6.5 mg), respectively. 

*Neosuberitenone A* (**1**): white crystals; [*α*]^22^_D_ 10.5 (*c* 0.2, MeOH); UV (MeOH/H_2_O) *λ*_max_ 210, 235 (sh) nm; ^1^H and ^13^C NMR spectra (CDCl_3_), see Table 1 and Table 2; (−)-HRESIMS *m*/*z* 473.2915 [M + HCOO]^−^ (calcd for C_28_H_41_O_6_, 473.2909; Δ 1.32 ppm).

*Suberitenone E* (**2**): white crystals; [*α*]^22^_D_ −42 (*c* 0.1, MeOH); UV (MeOH/H_2_O) *λ*_max_ 231 nm; ^1^H and ^13^C NMR spectra (CD_3_OD), see Table 1 and Table 2; (−)-HRESIMS *m*/*z* 489.2865 [M + HCOO]^−^ (calcd for C_28_H_41_O_7_, 489.2858; Δ 1.58 ppm).

*Suberitenone F* (**3**): white film; [*α*]^22^_D_ −45 (*c* 0.1, MeOH); UV (MeOH/H_2_O) *λ*_max_ 233 nm; ^1^H and ^13^C NMR spectra (CD_3_OD), see Table 1 and Table 2; (−)-HRESIMS *m*/*z* 489.2841 [M + HCOO]^−^ (calcd for C_28_H_41_O_7_, 489.2858; Δ 3.34 ppm).

*Suberitenone G* (**4**): white film; [*α*]^22^_D_ −62 (*c* 0.2, MeOH); UV (MeOH/H_2_O) *λ*_max_ 233 nm; ^1^H and ^13^C NMR spectra (CD_3_OD), see Table 1 and Table 2; (−)-HRESIMS *m*/*z* 441.2645 [M − H]^−^ (calcd for C_27_H_37_O_5_, 441.2646; Δ 0.42 ppm).

*Suberitenone H* (**5**): white film; [*α*]^22^_D_ −9.0 (*c* 0.2, MeOH); UV (MeOH/H_2_O) *λ*_max_ 233, 280 (sh) nm; ^1^H and ^13^C NMR spectra (CD_3_OD), see Table 1 and Table 2; (−)-HRESIMS *m*/*z* 523.3284 [M + HCOO]^−^ (calcd for C_29_H_47_O_8_, 523.3276; Δ 1.36 ppm).

*Suberitenone I* (**6**): white film; [*α*]^22^_D_ 7.7 (*c* 0.1, MeOH, 3:1 mixture with **7**); UV (MeOH/H_2_O) *λ*_max_ 210 nm; ^1^H and ^13^C NMR spectra (CD_3_OD), see Table 1 and Table 2; (−)-HRESIMS *m*/*z* 489.2855 [M + HCOO]^−^ (calcd for C_28_H_41_O_7_, 489.2858; Δ 0.54 ppm).

*Suberitenone J* (**7**): white film; [*α*]^22^_D_ 7.7 (*c* 0.1, MeOH, 3:1 mixture with **7**); UV (MeOH/H_2_O) *λ*_max_ 232 nm; ^1^H and ^13^C NMR (CD_3_OD) could not be fully assigned due to rapid conversion to **6** upon purification. The observed signals that differed from **6** are reported in Table S7; (−)-HRESIMS *m*/*z* 489.2855 [M + HCOO]^−^ (calcd for C_28_H_41_O_7_, 489.2858; Δ 0.54 ppm).

*Secosuberitenone A* (**8**): white film; [*α*]^22^_D_ −11.7 (*c 0.1*, MeOH); UV (MeOH/H_2_O) *λ*_max_ 230 nm; ^1^H and ^13^C NMR spectra (CDCl_3_), see Table 1 and Table 2; (−)-HRESIMS *m*/*z* 431.2814 [M + HCOO]^–^ (calcd for C_26_H_39_O_5_, 431.2803; Δ −2.51 ppm).

*Norsuberitenone A* (**9**): white crystalline solid; [*α*]^22^_D_ 6.3 (*c* 0.1, MeOH); UV (MeOH/H_2_O) *λ*_max_ 210, 237, 285 (sh) nm; ^1^H and ^13^C NMR spectra (CD_3_OD), see Table S9; (+)-HRESIMS *m*/*z* 279.2320 [M + H]^+^ (calcd for C_18_H_31_O_2_, 279.2319; Δ 0.38 ppm).

### 3.3. X-ray Crystallography

Crystallographic data for the structures reported in this article were deposited at the Cambridge Crystallographic Data Center under the deposition numbers CCDC 2164632 (**1**) and 2164633 (**2**). Copies of the data can be obtained free of charge via https://www.ccdc.cam.ac.uk/structures/ (accessed on 5 April 2022). XRD data were measured on Bruker D8 Venture PHOTON II CMOS diffractometer equipped with a Cu Kα INCOATEC ImuS micro-focus source (λ = 1.54178 Å). Indexing was performed using APEX4 (difference vectors method) [15]. Data integration and reduction were performed using SaintPlus [16]. Absorption correction was performed by the multi-scan method implemented in SADABS [17]. Space group was determined using XPREP implemented in APEX3 [15]. The structure was solved using SHELXT [18] and refined using SHELXL-2018/3 (full-matrix least-squares on F2) [19] through the OLEX2 interface program [20]. The ellipsoid plot was completed with Platon [21].

*Neosuberitenone A* (**1**): A hydrogen atom of the -OH group was found from a difference Fourier map and was freely refined. All remaining hydrogen atoms were refined using a riding model. Crystal data: C_27_H_40_O_4_, M = 428.59 g/mol, monoclinic, space group C2, a = 24.7343(6) Å, b = 6.27570(10) Å, c = 18.2166(4) Å, V = 2308.80(9) Å^3^, Z = 4, T = 100.00 K, μ(Cu Kα) = 0.636 mm^−1^, ρ_calc_ = 1.233 g/cm^3^, 21421 reflections measured (5.942° ≤ 2θ ≤ 159.866°), 4852 independent reflections (R_int_ = 0.0409, R_sigma_ = 0.0334), which were used in all of the calculations. The final R_1_ was 0.0330 (I >= 2σ(I)) and the wR_2_ was 0.0881 (all data). Flack parameter: 0.06(6). A full table of these parameters for the crystal structure can be found in the supplementary information.

*Suberitenone E* (**2**): A hydrogen atom of the -OH group was found from a difference Fourier map and was refined with distance restraint. All remaining hydrogen atoms were refined using a riding model. Crystal data: C_27_H_40_O_5_, M = 444.59 g/mol, monoclinic, space group P2_1_, a = 6.7821(2) Å, b = 8.9780(3) Å, c = 20.5457(6) Å, V = 1245.06(7) Å^3^, Z = 2, T = 298.00 K, μ(Cu Kα) = 0.638 mm^−1^, ρ_calc_ = 1.186 g/cm^3^, 28530 reflections measured (8.648° ≤ 2θ ≤ 158.82°), 5185 independent reflections (R_int_ = 0.0572, R_sigma_ = 0.0426), which were used in all of the calculations. The final R_1_ was 0.0465 (I >= 2σ(I)) and the wR_2_ was 0.1325 (all data). Flack parameter: 0.04(11). A full table of these parameters for the crystal structure can be found in the Supplementary Materials.

### 3.4. RSV Antiviral Assay

A549 (CCL-185, ATCC) cells were seeded at 1.6 × 10^4^ cells in 100 μL of 5% FBS/1X penicillin–streptomycin/F12 medium per well in 96-well µClear® black plates with a clear bottom (Greiner 655090). The cells were infected with 400 PFU/well of a recombinant RSV encoding *Renilla* luciferase as an additional transcription unit (rA2-Rluc [22]) in a total volume of 50 µL/well and allowed to adsorb for 1 h. Purified compounds were serially diluted two-fold in triplicate and 50 µL/well of the diluted extracts were added to the infected cells. After further incubation at 37 °C and 5% CO_2_ for 24 h, the supernatants were removed and cells were lysed using *Renilla* lysis buffer (Promega). Luciferase activity was measured with the *Renilla* Luciferase reagent (Promega) and BioTek Synergy Mx microplate luminometer using Gen5 version 2.00.18 software. The RSV antiviral effect for the test samples was determined by normalizing to the DMSO-treated control samples and multiplying by 100 to obtain the percent of control. Statistical analysis was performed using GraphPad Prism software version 9.4.1.

### 3.5. Cytotoxicity Assay

To test for cell viability, duplicate 96-well plates of uninfected A549 cells were set up in parallel with the RSV antiviral assay. Extracts were diluted and used to treat cells as described above for the RSV antiviral assay. The negative control for cytotoxicity was 2% DMSO while the positive was 2 µM (final concentration) of PDKI inhibitor. After the 24 h incubation period, the cells were processed for an MTT Proliferation Assay (Provost and Wallert Research) according to the manufacturer’s protocol. Absorbance at 570 nm was measured by a microplate luminometer and standardized to control as above. Two percent DMSO and 2 µM PDK1 inhibitor (CalBioChem) were used as negative and positive controls for cell death, respectively. Statistical analysis was performed using GraphPad Prism software version 9.4.1.

## 4. Conclusions

In summary, a ^1^H NMR-guided fractionation procedure of the polar extract constituents of the Antarctic sponge *Suberites* sp. resulted in the isolation of nine new sesterterpenoids; neosuberitenone A (**1**), suberitenones E–J (**2**–**7**), secosuberitenone A (**8**), and norsuberitenone A (**9**) along with large quantities of suberitenones A (**10**) and B (**11**). The previously unreported compounds showed various oxidations and ring formations never before reported from this class of compounds, including the new neosuberitane terpenoid carbon skeleton solved by a combination of NMR interpretation and XRD data. Although the compounds did not show activity in antibacterial or antifungal assays, they showed the ability to inhibit RSV viral transcription with relatively low levels of cytotoxicity. The new compounds demonstrated weaker activity than suberitenones A and B and provide important information for future structure–activity relationship studies and structure optimization.

## Figures and Tables

**Table 1:** ^13^C NMR spectroscopic data of compounds **1**–**6** and **8**.

Position	1 ^a,c^	2 ^b,c^	3 ^b,d^	4 ^b,d^	5 ^b,d^	6 ^b,d^	8 ^a,d^
1	67.5, CH	65.9, CH	66.0, CH	64.7, CH	66.6, CH	71.0, CH	63.6, CH
2	42.9, CH_2_	145.5, CH	145.8, CH	145.5, CH	88.6, CH	79.9, CH	141.7, CH
3	48.7, C	136.9, C	136.9, C	136.8, C	44.6, CH	48.1, CH	137.6, C
4	214.3, C	201.6, C	202.6, C	202.2, C	214.6, C	212.8, C	200.0 C
5	41.9, CH_2_	35.3, CH_2_	38.5, CH_2_	37.7, CH_2_	37.4, CH_2_	46.7, CH_2_	37.3, CH_2_
6	44.1, CH	47.9, CH	45.4, CH	38.8, CH	48.3, CH	45.5, CH	45.5, CH
7	134.3, C	63.4, C	137.2, C	135.7, C	75.1, C	132.0, C	148.8, C
8	40.8, CH	26.7, CH_2_	128.9, CH	147.2, CH	38.7, CH_2_	70.8, CH	34.2, CH_2_
9	21.5, CH_2_	16.9, CH_2_	24.1, CH_2_	24.6, CH_2_	18.0, CH_2_	26.0, CH_2_	22.5, CH_2_
10	55.1, CH	49.2, CH	46.8, CH	54.7, CH	59.7, CH	55.1, CH	57.5, CH
11	35.7, C	34.8, C	38.0, C	45.7, C	35.5, C	37.1, C	144.4, C
12	45.5, CH_2_	42.5, CH_2_	39.8, CH_2_	39.4, CH_2_	48.1, CH_2_	44.3, CH_2_	47.8, CH_2_
13	70.8, CH	72.0, CH	72.3, CH	71.3, CH	72.3, CH	72.1, CH	69.5, CH
14	57.1, CH	57.5, CH	57.2, CH	56.5, CH	57.7, CH	57.7, CH	57.6, CH
15	38.2, C	38.0, C	38.0, C	39.0, C	38.3, C	38.0, C	41.2, C
16	41.7, CH_2_	43.1, CH_2_	43.5, CH_2_	42.6, CH_2_	43.0, CH_2_	42.6, CH_2_	42.2, CH_2_
17	18.4, CH_2_	19.6, CH_2_	19.6, CH_2_	19.4, CH_2_	19.7, CH_2_	19.5, CH_2_	19.7, CH_2_
18	44.2, CH	45.2, CH_2_	45.1, CH_2_	44.9, CH_2_	45.4, CH_2_	45.3, CH_2_	44.0, CH_2_
19	34.0, C	35.0, C	34.8, C	34.8, C	35.0, C	35.0, C	34.6, C
20	33.2, CH_3_	33.4, CH_3_	33.8, CH_3_	33.5, CH_3_	33.3, CH_3_	33.1, CH_3_	33.8, CH_3_
21	16.4, CH_3_	15.7, CH_3_	15.6, CH_3_	15.6, CH_3_	10.4, CH_3_	11.6, CH_3_	15.8, CH_3_
22	140.0, CH	70.1, CH	75.3, CH	205.5, C	55.3, CH_2_	140.9, CH	112.4, CH_2_
23	19.6, CH_3_	19.7, CH_3_	20.8, CH_3_	19.6, CH_3_	23.4, CH_3_	23.7, CH_3_	110.4, CH_2_
24	16.1, CH_3_	17.9, CH_3_	17.6, CH_3_	18.1, CH_3_	17.9, CH_3_	18.1, CH_3_	17.3, CH_3_
25	23.2, CH_3_	23.6, CH_3_	23.8, CH_3_	23.7, CH_3_	23.7, CH_3_	23.5, CH_3_	23.8, CH_3_
26	170.6, C	172.3, C	172.4, C	172.0, C	172.3, C	172.1, C	
27	22.0, CH_3_	21.8, CH_3_	21.9, CH_3_	21.8, CH_3_	21.8, CH_3_	21.8, CH_3_	
28					59.1, CH_3_		

^a^: CDCl_3_; ^b^: CD_3_OD; ^c^: 125 MHz; ^d^: 150 MHz.

**Table 2:** ^1^H NMR spectroscopic data of compounds **1**–**6** and **8**.

Position	1 ^a,c^	2 ^b,c^	3 ^b,d^	4 ^b,c^	5 ^b,c^	6 ^b,c^	8 ^a,d^
1	4.18, dd (8.7, 3.5)	4.40, t (4.4)	4.30, dd (5.6, 3.3)	4.14, br t (4.2)	4.46, br t (3.3)	4.17 br t (2.7)	4.31, br t
2a	2.13, o/l	6.76, dq (5.4, 1.3)	6.85, dq (5.5, 1.4)	6.80, dq (5.6, 1.5)	3.52, t (3.6)	4.03, br s	6.78, br d (4.4)
2b	1.64, o/l				3.03, m	2.64, m	
5a	2.25, d (18.6)	2.73, dd (16.6, 12.4)	2.83, dd (15.9, 12.8)	2.82, dd (16.2, 13.4)	2.65, t (13.7)	2.83, dd (16.2, 5.2)	2.84, m
5b	2.10, o/l	2.37, dd (16.6, 4.1)	2.31, (16.1, 2.8)	2.18, dd (16.2, 3.6)	2.23, dd (13.8, 4.7)	2.30, dd (16.2, 2.5)	2.36, o/l
6	2.69, o/l	1.90, o/l	2.90, m	3.39, m	1.81, o/l	2.68, m	2.75, m
8a	2.68, o/l	2.35, o/l	5.67, t (3.2)	6.78, m	1.91, o/l	3.79, dd (9.4, 6.6)	2.35, o/l
8b		1.71, o/l			1.21, o/l		1.86 o/l
9a	1.75, m	1.43, o/l	2.12, o/l	2.52, m	1.66, td (12.8, 3.4)	1.90, o/l	1.76, o/l
9b	1.26, o/l				1.57, m	1.67, td (12.4, 9.5)	1.52, o/l
10	1.02, o/l	1.28, m	1.60, dd (11.3, 5.9)	1.71, o/l	0.99, dd (12.3, 2.5)	1.13, o/l	1.68, m
12a	2.09, o/l	1.87, o/l	2.16, o/l	2.13, dd (15.5, 2.7)	1.88, o/l	1.88, o/l	2.34, o/l
12b	1.44, dd (15.1, 3.7)	1.65, dd (14.6, 3.6)	1.53, dd (15.0, 2.7)	1.69, o/l	1.32, m	1.41, dd (14.6, 3.7)	
13	5.51, br q (2.5)	5.55, br q (2.5)	5.61, dt (4.4, 2.4)	5.61, q (3.2)	5.48, br q (3.2)	5.49, q (3.1)	4.38, br t
14	1.04, o/l	1.07, br d (2.0)	1.13, d (2.1)	1.13, m	1.14, br s	1.12, o/l	1.08, o/l
16a	1.55, br d (13)	1.69, m	1.72, o/l	1.76, o/l	1.77, o/l	1.74, o/l	1.78, o/l
16b	0.82, td (13.1, 3.1)	0.87, m	0.99, o/l	0.98, m	0.92, o/l	0.85, td (13.1, 3.9)	1.07, o/l
17a	1.66, o/l	1.74, o/l	1.73, o/l	1.77, o/l	1.77, o/l	1.78, o/l	1.64, m
17b	1.41, o/l	1.45, o/l	1.46, m	1.49, m	1.47, m	1.49, m	1.50, m
18a	1.35, br d (13.1)	1.36, m	1.39, m	1.40, m	1.37, o/l	1.36, m	1.39, m
18b	1.14, o/l	1.22, m	1.24, td (13.1, 3.9)	1.24, m	1.23, o/l	1.23, o/l	1.19, m
20	0.92, s	0.92, s	0.96, s	0.95, s	0.91, s	0.91, s	1.01, s
21	1.01, s	1.76, s	1.78, s	1.79, s	1.02, d (6.8)	1.19, d (6.8)	1.84, s
22a	5.60, d (3.1)	2.63, br s	3.15, s		1.81, o/l	5.24, s	5.16, s
22b					1.15/ o/l		4.92, s
23a	1.18, s	1.27, s	0.99, s	1.27, s	1.37, s	1.26, s	5.03, s
23b							4.77, s
24	1.28, s	1.21, s	1.34, s	1.42, s	1.23, s	1.24, s	1.00, s
25	1.01, s	1.02, s	1.04, s	1.04, s	1.04, s	1.03, s	1.22, s
27	2.06, s	2.05, s	2.04, s	2.04, s	2.03, s	2.04, s	
28					3.35, s		

^a^: CDCl_3_; ^b^: CD_3_OD; o/l = overlapped resonances.; ^c^: 500 MHz; ^d^: 600 MHz.

**Table 3:** RSV antiviral (IC_50_) and cytotoxicity (CC_50_) activities of **2**–**5**, **10,** and **11** (means ± SEM of triplicate samples). Calculated from data in Figure S12 using Prism. Selectivity index = CC_50_/IC_50_.

Compound	IC_50_ (mM)	CC_50_ (mM)	Selectivity Index
2	20.5 ± 0.7	87.3 ± 38.1	4.3
3	9.8 ± 1.4	36.0 ± 5.3	3.7
4	11.0 ± 1.3	40.7 ± 8.3	3.7
5	10.9 ± 1.8	51.1 ± 17.5	4.7
10	7.8 ± 0.2	33.3 ± 1.3	4.3
11	3.2 ± 0.4	67.6 ± 16.3	21.1

## Data Availability

Data generated in the process of this research are available in the Supplementary Materials.

## Supplementary Materials

The following supporting information can be downloaded at: https://www.mdpi.com/article/10.3390/md21020107/s1. Figures S1–S7. NMR spectra and MS data for neosuberitenone A (**1**); Figures S8–S14. NMR spectra and MS data for suberitenone E (**2**); Figures S15–S21. NMR spectra and MS data for suberitenone F (**3**); Figures S22–S28. NMR spectra and MS data for suberitenone G (**4**); Figures S29–S35. NMR spectra and MS data for suberitenone H (**5**); Figures S36–S44. NMR spectra and MS data for suberitenone I (**6**) and suberitenone J (**7**); Figures S45–S51. NMR spectra and MS data for secosuberitenone A (**8**); Figures S52–S58. NMR spectra and MS data for norsuberitenone A (**9**); Figure S59. Ellipsoid plot of neosuberitenone A (**1**); Figure S60. Ellipsoid plot of suberitenone E (**2**); Figure S61. Antiviral activity and cytotoxicity of selected compounds. A549 Cells were infected with rA2-Rluc for 1 h (a) or left uninfected (b) then treated in triplicate with serially diluted purified compounds. 24 h post-infection, antiviral activity was determined by Renilla luciferase assay (a) and cytotoxicity was measured by MTT assay (b). Values are normalized to DMSO-treated cells. Shown are means ± SEM; Table S1. NMR data for neosuberitenone A (**1**); Table S2. NMR data for suberitenone E (**2**); Table S3. NMR data for suberitenone F (**3**); Table S4. NMR data for suberitenone G (**4**); Table S5. NMR data for suberitenone H (**5**); Table S6. NMR data for suberitenone I (**6**); Table S7. NMR data for suberitenone J (**7**); Table S8. NMR data for secosuberitenone A (**8**); Table S9. NMR data for norsuberitenone A (**9**); Table S10. Crystal data for neosuberitenone A (**1**); Table S11. Crystal data for suberitenone E (**2**); Table S12. Antiviral activity against RSV.
