# Supplementary material for: Neosuberitenone, a New Sesterterpenoid Carbon Skeleton; New Suberitenones; and Bioactivity against Respiratory Syncytial Virus, from the Antarctic Sponge Suberites sp
Source: Mar Drugs. 2023 Feb 1;21(2):107. doi: 10.3390/md21020107 (PMC9964336; doi:10.3390/md21020107)
Supplement: Supplementary file 1 [file marinedrugs-21-00107-s001.zip › marinedrugs-2154342-supplementary.pdf]

Neosuberitenone, a New Sesterterpenoid Carbon Skeleton, New Suberitenones, and Bioactivity against Respiratory Syncytial Virus, from the Antarctic sponge *Suberites* sp.

Joe Bracegirdle [1], Stine S. H. Olsen [1], Michael N. Teng [2], Kim C. Tran [2], Charles D. Amsler [3], James B. McClintock [3] and Bill. J. Baker [1,*]

[1] Department of Chemistry, University of South Florida, 4202 E. Fowler Avenue, CHE205, Tampa, FL 33620, USA
[2] Department of Internal Medicine, University of South Florida, Tampa, FL 33612, USA
[3] Department of Biology, University of Alabama at Birmingham, 1300 University Blvd, Birmingham, AL 35233, USA
* Correspondence: bjbaker@usf.edu; Tel.: +1 (813) 974-1967

Table of Contents

Table S1. NMR data for neosuberitenone A (**1**) 2
Figure S1 – S7. NMR spectra and MS data for **1** 3
Table S2. NMR data for suberitenone E (**2**) 8
Figure S8 – S14. NMR spectra and MS data for **2** 9
Table S3. NMR data for suberitenone F (**3**) 14
Figure S15 – S21. NMR spectra and MS data for **3** 15
Table S4. NMR data for suberitenone G (**4**) 20
Figure S22 – S28. NMR spectra and MS data for **4** 21
Table S5. NMR data for suberitenone H (**5**) 26
Figure S29 – S35. NMR spectra and MS data for **5** 27
Table S6. NMR data for suberitenone I (**6**) 32
Table S7. NMR data for suberitenone J (**7**) 33
Figure S36 – S44. NMR spectra and MS data for **6** and **7** 34
Table S8. NMR data for secosuberitenone A (**8**) 41
Figure S45 – S51. NMR spectra and MS data for **8** 42
Table S9. NMR data for norsuberitenone A (**9**) 47
Figure S52 – S58. NMR spectra and MS data for **9** 48
Table S10. Crystal data for neosuberitenone A (**1**) 53
Table S11. Crystal data for suberitenone E (**2**) 54
Figure S59. Ellipsoid plot of neosuberitenone A (**1**) 55
Figure S60. Ellipsoid plot of suberitenone E (**2**) 55
Figure S61. Antiviral activity and cytotoxicity of selected compounds 56
Table S12. Antiviral activity against RSV 56

Table S1 – NMR data for neosuberitenone A (**1**) (500 (^{1}H) and 125 (^{13}C) MHz, $CDCl_3$).

pos	δ_C, type	δ_H	gCOSY	gHMBC	NOESY
**1**	67.5, CH	4.18, dd (3.5, 8.7)	2, 2', 6	7	5
**2**	42.9, CH_2	2.13, o/l	1, 2'	3, 4, 8	
**2'**		1.64, o/l	1, 2	1, 3, 4, 8, 21	8
**3**	48.7, C				
**4**	214.3, C				
**5**	41.9, CH_2	2.25, d (18.6)	5', 6	1, 4, 6, 7	1
**5'**		2.10, o/l	5, 6	1, 6, 7	
**6**	44.1, CH	2.69, o/l	1, 5, 5'	1, 2, 4, 7, 8, 22	
**7**	134.3, C				
**8**	40.8, CH	2.68, o/l	9, 9', 22	2, 3, 4, 22	2'
**9**	21.5, CH_2	1.75, m	8, 9', 10	7, 8, 10, 15	
**9'**		1.26, o/l	8, 9, 10	3, 8, 11	
**10**	55.1, CH	1.02, o/l	9, 9'	9, 11, 23, 24	12', 22
**11**	35.7, C				
**12**	45.5, CH_2	2.09, o/l	12', 13	10, 11, 13, 14, 23	22, 23
**12'**		1.44, dd (3.7, 15.1)	12, 13	11, 22, 23	10, 14, 22
**13**	70.8, CH	5.51, br q (2.5)	12, 12', 14		20
**14**	57.1, CH	1.04, o/l	13	15, 19, 24, 25	12', 20
**15**	38.2, C				
**16**	41.7, CH_2	1.55, br d (13)	16', 17, 17'		
**16'**		0.82, td (13.1, 3.1)	16, 17, 17'	10, 15, 17, 24	
**17**	18.4, CH_2	1.66, o/l	16, 17', 18, 18'		25
**17'**		1.41, o/l	16, 16', 17		
**18**	44.2, CH	1.35, br d (13.1)	17, 18'		20, 25
**18'**		1.14, o/l	17', 18	17, 25	
**19**	34.0, C				
**20**	33.2, CH_3	0.92, s		14, 18, 19, 25	13, 14, 18
**21**	16.4, CH_3	1.01, s		2, 3, 4, 8	
**22**	140.0, CH	5.60, d (3.1)	8	6, 8, 11, 12	10, 12, 12', 23
**23**	19.6, CH_3	1.18, s		10, 11, 12, 22	12, 22, 24, 27
**24**	16.1, CH_3	1.28, s		10, 14, 15, 16	23, 25, 27
**25**	23.2, CH_3	1.01, s		14, 18, 19, 20	17, 18, 24, 27
**26**	170.6, C				
**27**	22.0, CH_3	2.06, s		26	23, 24, 25

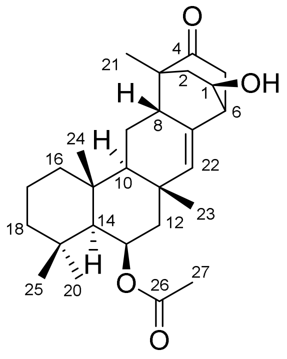

Figure S1 – ^{1}H NMR spectrum (500 MHz, $CDCl_3$) of **1**

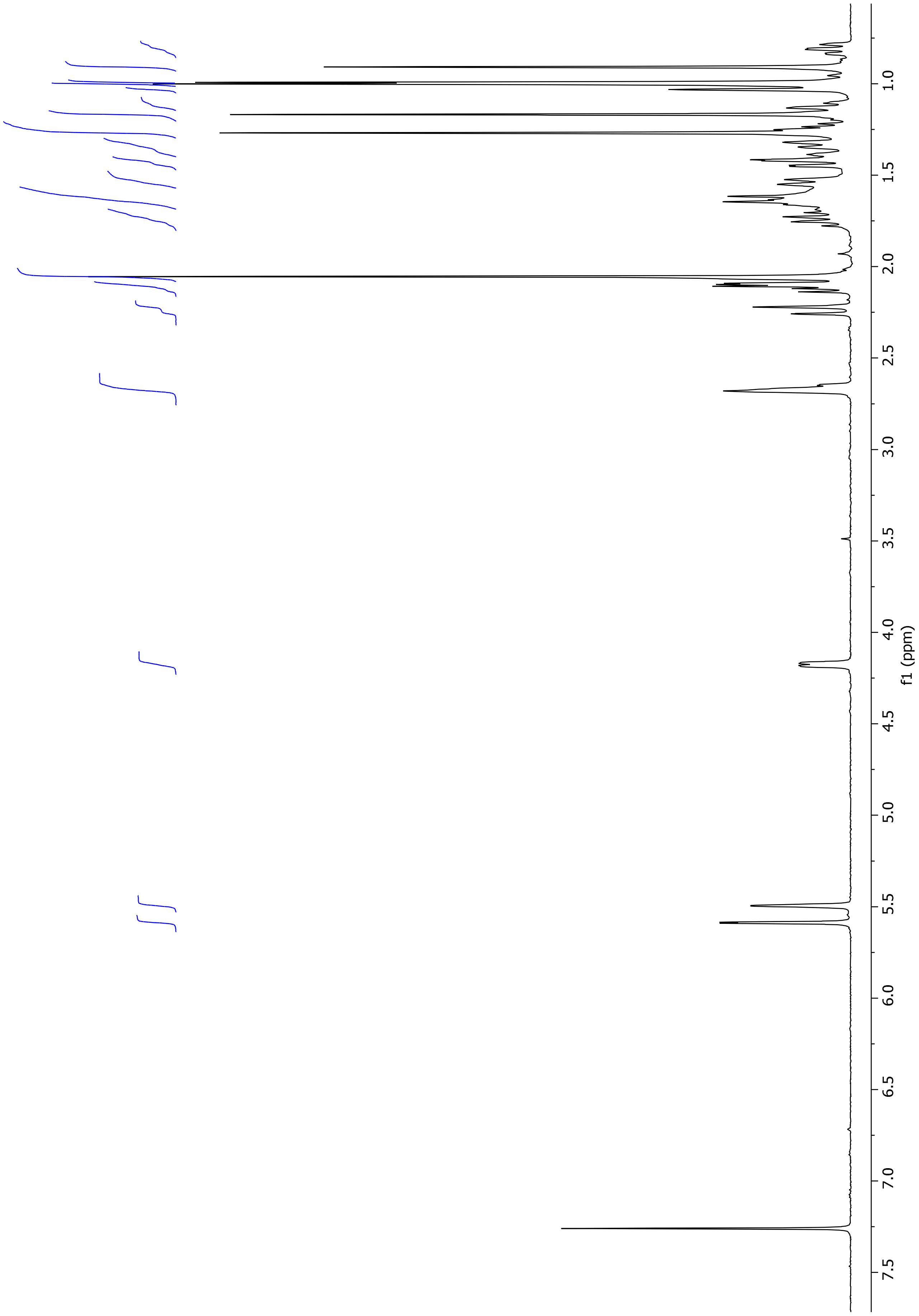

Figure S2 – ^{13}C NMR spectrum (125 MHz, $CDCl_3$) of **1**

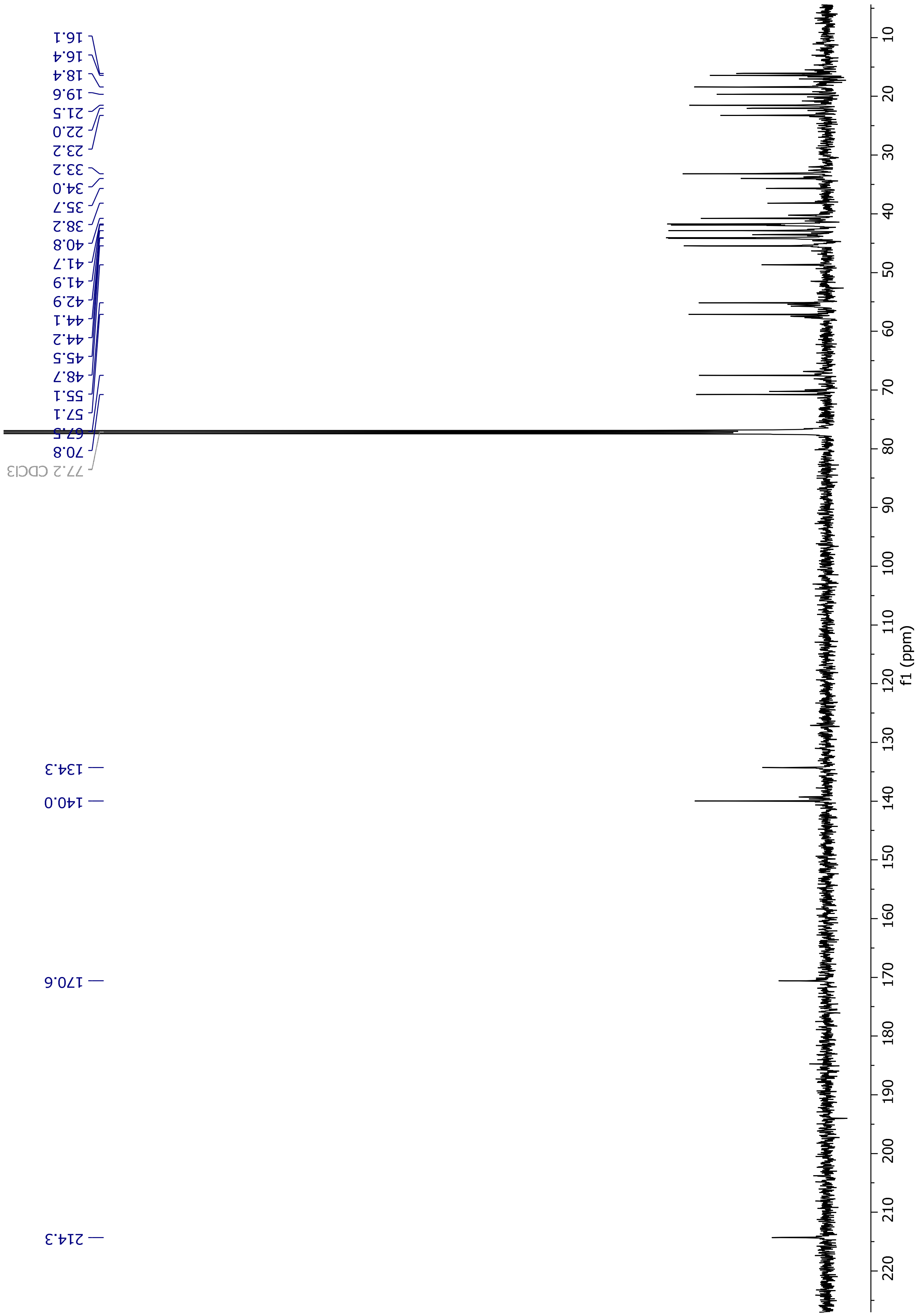

Figure S3 – COSY NMR spectrum (500 MHz, $CDCl_3$) of **1**

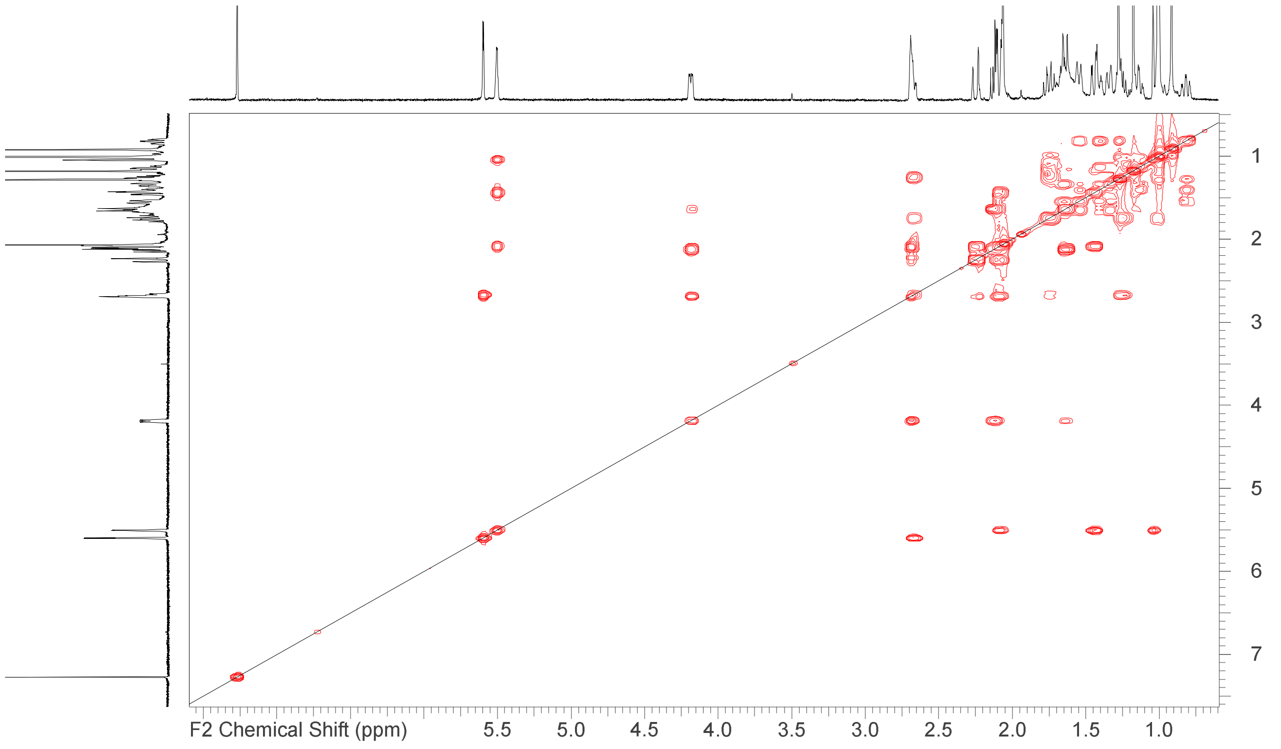

Figure S4 – HSQC NMR spectrum (500 MHz, $CDCl_3$) of **1**

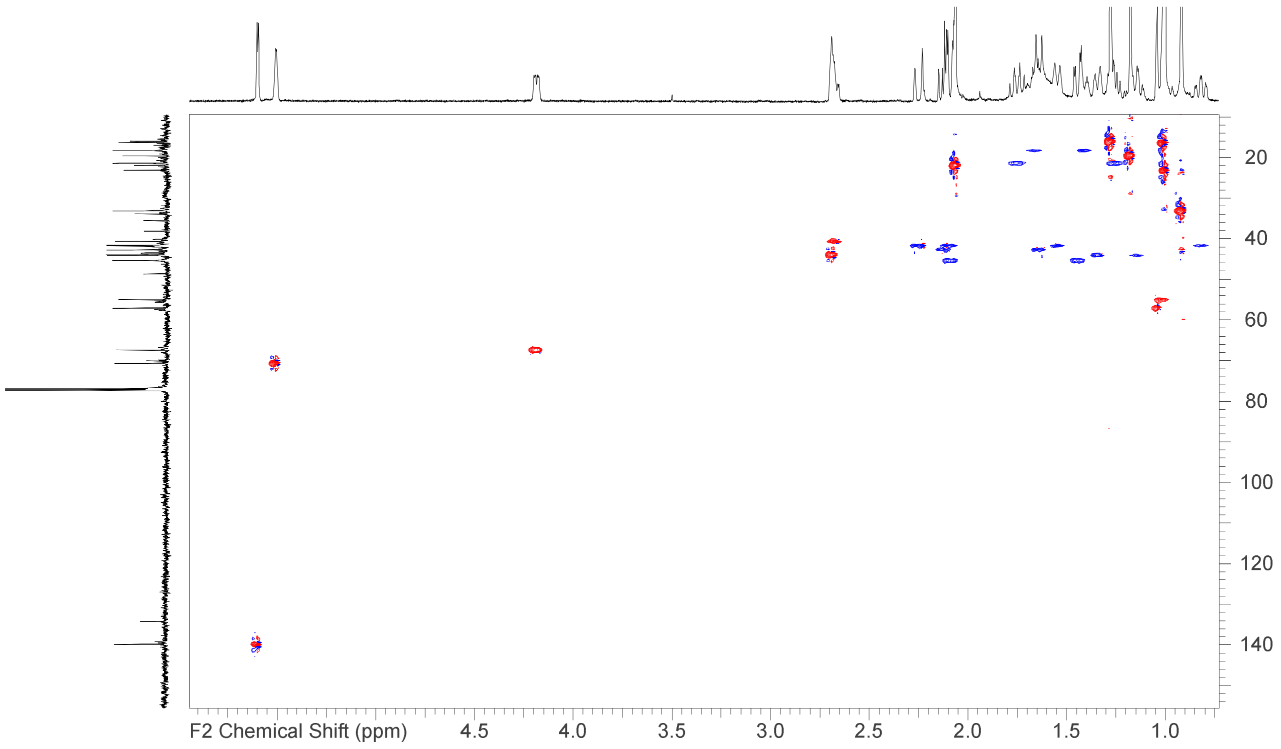

Figure S5 – HMBC NMR spectrum (500 MHz, $CDCl_3$) of **1**

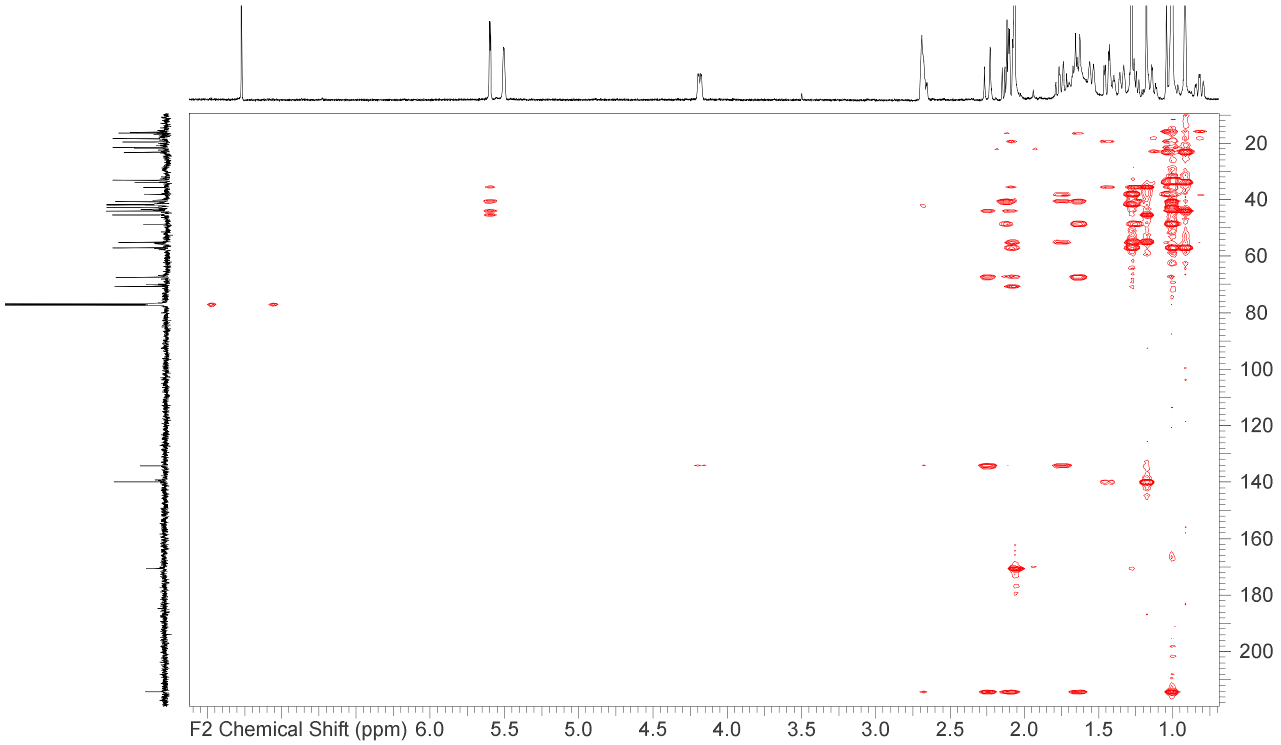

Figure S6 – NOESY NMR spectrum (600 MHz, $CDCl_3$) of **1**

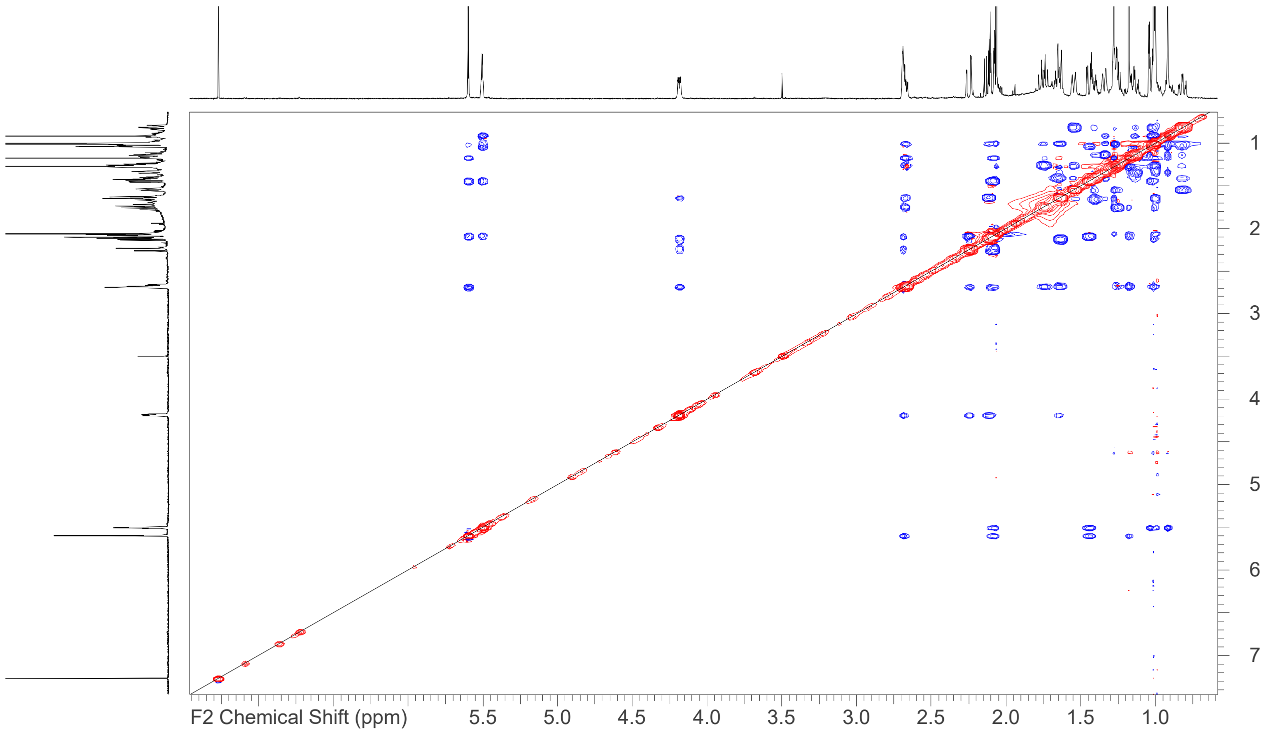

Figure S7 – HRESIMS analysis of **1**

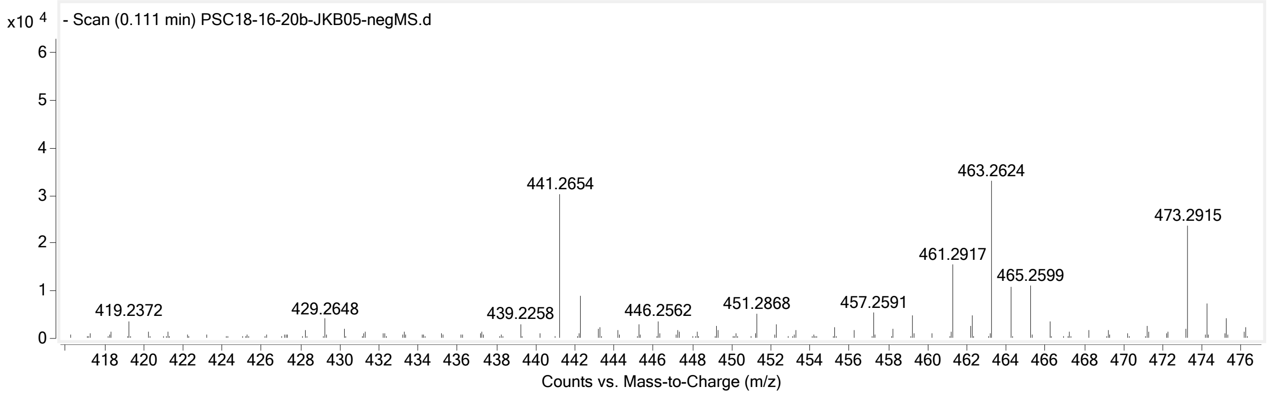

		C27 H40 O4	93.9	428.29332			-1.54	1.54	-0.66	0.111

Species	Ion Formula	m/z	Height	Score (MFG)	Score (MS)	Score (mass)	Score (iso. abund)	Score (iso. spacing)
(M-H)-		427.2805	199.1		14	29.41	0	0
(M+Cl)-		463.2628	14983.1		82.58	98.37	44.66	96.5
(M+HCOO)-		473.2915	12188.9		93.9	96.88	88.47	94.44

m/z	m/z (Calc)	Diff (ppm)	Diff (mDa)	Height	Height (Calc)	Height %	Height % (Calc)	Height Sum %	Height Sur
473.2915	473.2909	-1.32	-0.6	11783.3	12188.9	100	100	70.6	
474.2952	474.2943	-2.02	-1	3760.4	3776.6	31.9	31	22.5	
475.2996	475.2971	-5.26	-2.5	1137.2	715.4	9.7	5.9	6.8	

Table S2 – NMR data for suberitenone E (**2**) (500 (^{1}H) and 125 (^{13}C) MHz, CD_3OD).

pos	δ_C, type	δ_H	gCOSY	gHMBC	NOESY
**1**	65.9, CH	4.40, t (4.4)	2, 6	2, 3, 5, 6, 7	23, 22, 8
**2**	145.5, CH	6.76, dq (5.4, 1.3)	1, 21	1, 4, 6, 21	6, 5, 5'
**3**	136.9, C				
**4**	201.6, C				
**5**	35.3, CH_2	2.73, dd (16.6, 12.4)	5', 6	1, 4, 6, 7	8', 2
**5'**		2.37, dd (16.6, 4.1)	5, 6	1, 3, 4, 6, 7	2
**6**	47.9, CH	1.90, o/l	1, 5, 5'	1, 4, 5, 7, 8	22, 2
**7**	63.4, C				
**8**	26.7, CH_2	2.35, o/l	8', 9	9, 10	1
**8'**		1.71, o/l	8, 9	7, 9, 22	5
**9**	16.9, CH_2	1.43, o/l	8, 8', 10	7, 8, 10, 11	22
**10**	49.2, CH	1.28, m	9	8, 9, 15, 23, 24	14, 16'
**11**	34.8, C				
**12**	42.5, CH_2	1.87, o/l	12', 13	10, 11, 13, 14, 23	23
**12'**		1.65, dd (14.6, 3.6)	12, 13	11, 22, 23	14, 22
**13**	72.0, CH	5.55, br q (2.5)	12, 12', 14	11, 15	20
**14**	57.5, CH	1.07, br d (2.0)	13	10, 15, 16, 18, 19, 20, 24, 25	10, 12', 20, 16'
**15**	38.0, C				
**16**	43.1, CH_2	1.69, m	16', 17, 17'	18	
**16'**		0.87, m	16, 17'	10, 15, 17, 24	10, 14
**17**	19.6, CH_2	1.74, o/l	16, 17', 18, 18'	16	25, 24
**17'**		1.45, o/l	16, 16', 17, 18, 18'		
**18**	45.2, CH_2	1.36, m	17, 17', 18'		20, 25
**18'**		1.22, m	17, 17', 18		
**19**	35.0, C				
**20**	33.4, CH_3	0.92, s		14, 18, 19, 25	13, 14, 18
**21**	15.7, CH_3	1.76, s		2, 3, 4	
**22**	70.1, CH	2.63, br s		6, 7, 10, 11, 12, 23	1, 6, 12', 9, 23
**23**	19.7, CH_3	1.27, s		10, 11, 12, 22	1, 12, 22, 17
**24**	17.9, CH_3	1.21, s		10, 14, 15, 16	17
**25**	23.6, CH_3	1.02, s		14, 18, 19, 20	17, 18
**26**	172.3, C				
**27**	21.8, CH_3	2.05, s		26	25, 23, 24

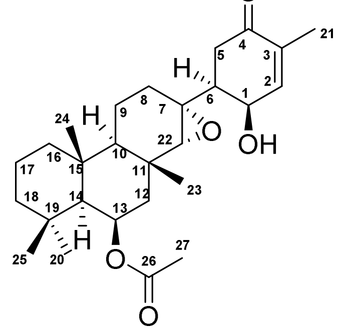

Figure S8 – ^{1}H NMR spectrum (500 MHz, CD_3OD) of **2**

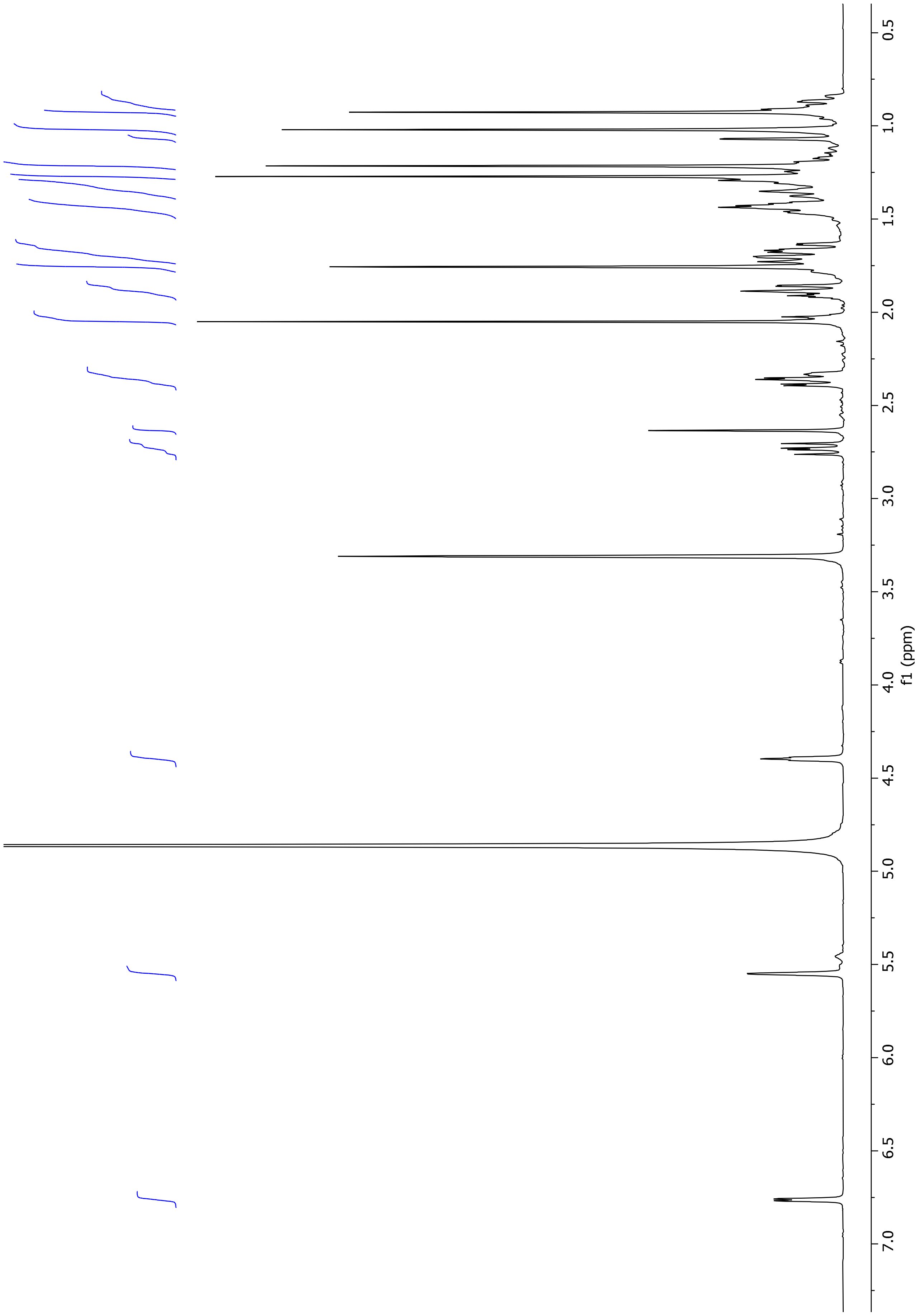

Figure S9 – ^{13}C NMR spectrum (150 MHz, CD_3OD) of **2** (formic acid impurity δ_C 170.3)

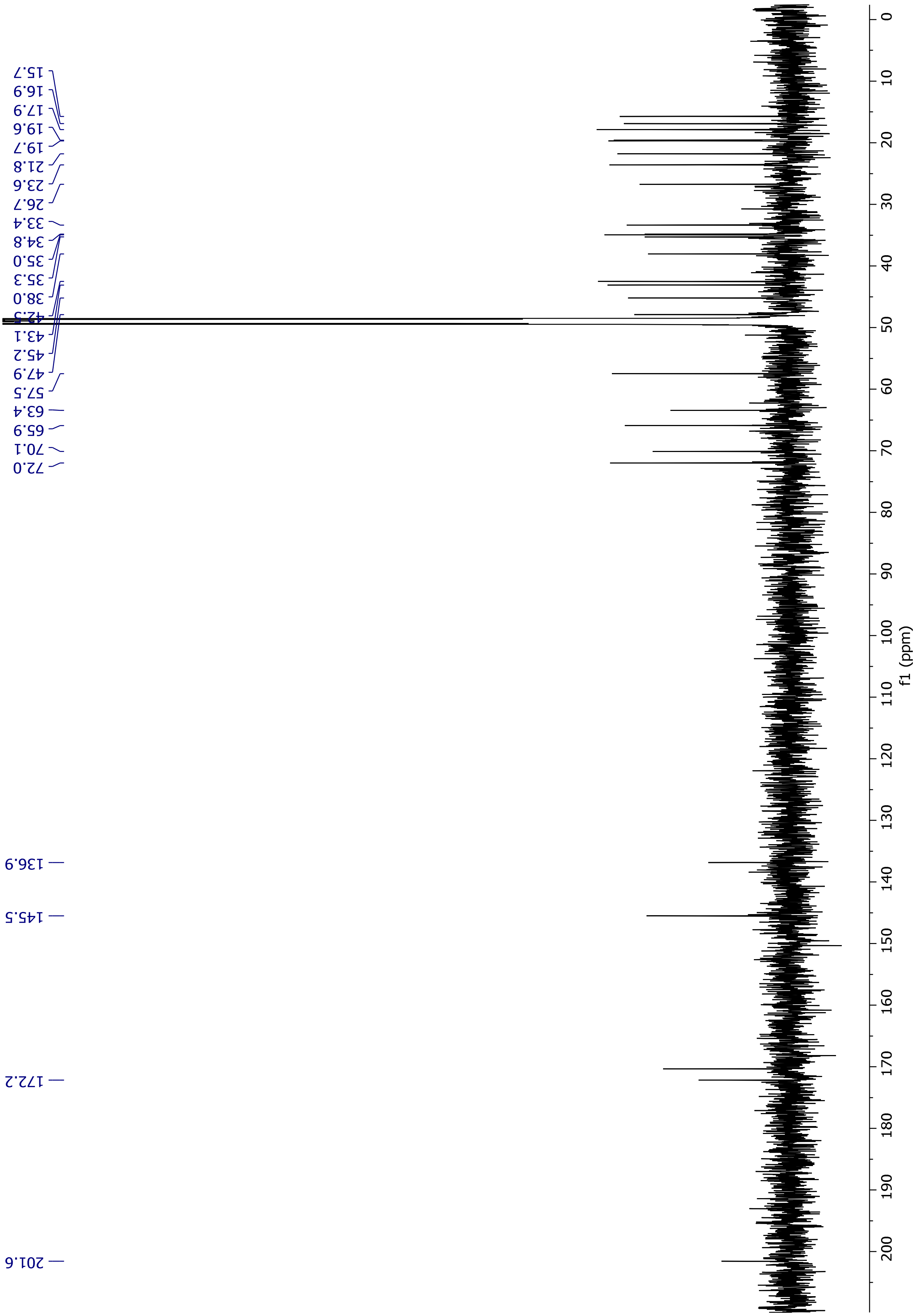

Figure S10 – COSY NMR spectrum (500 MHz, CD_3OD) of **2**

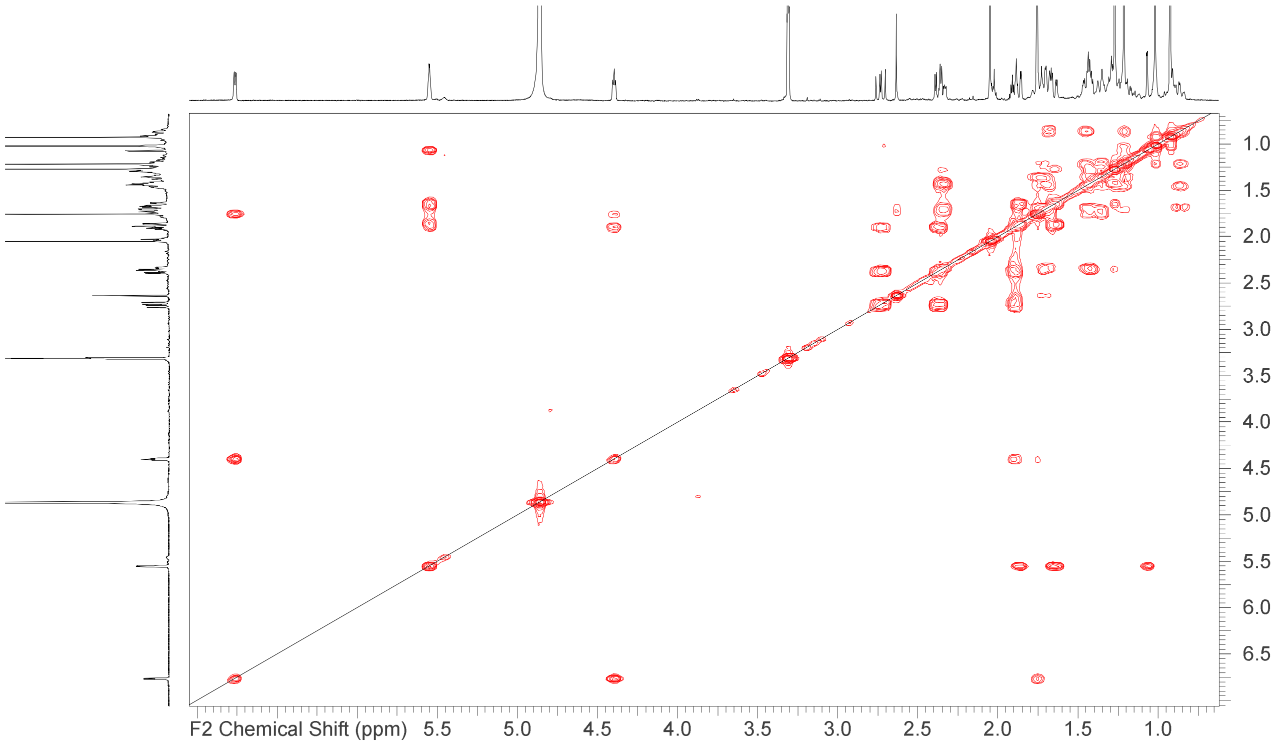

Figure S11 – HSQC NMR spectrum (500 MHz, CD_3OD) of **2**

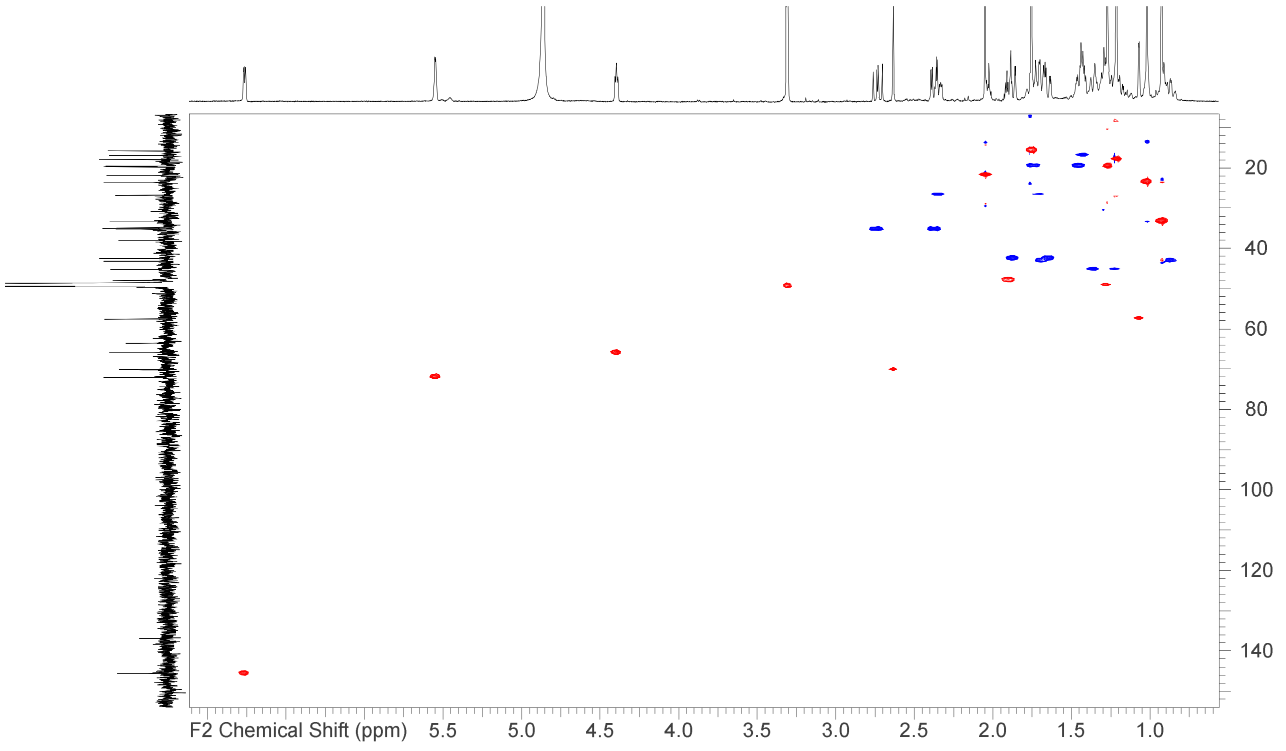

Figure S12 – HMBC NMR spectrum (500 MHz, CD_3OD) of **2**

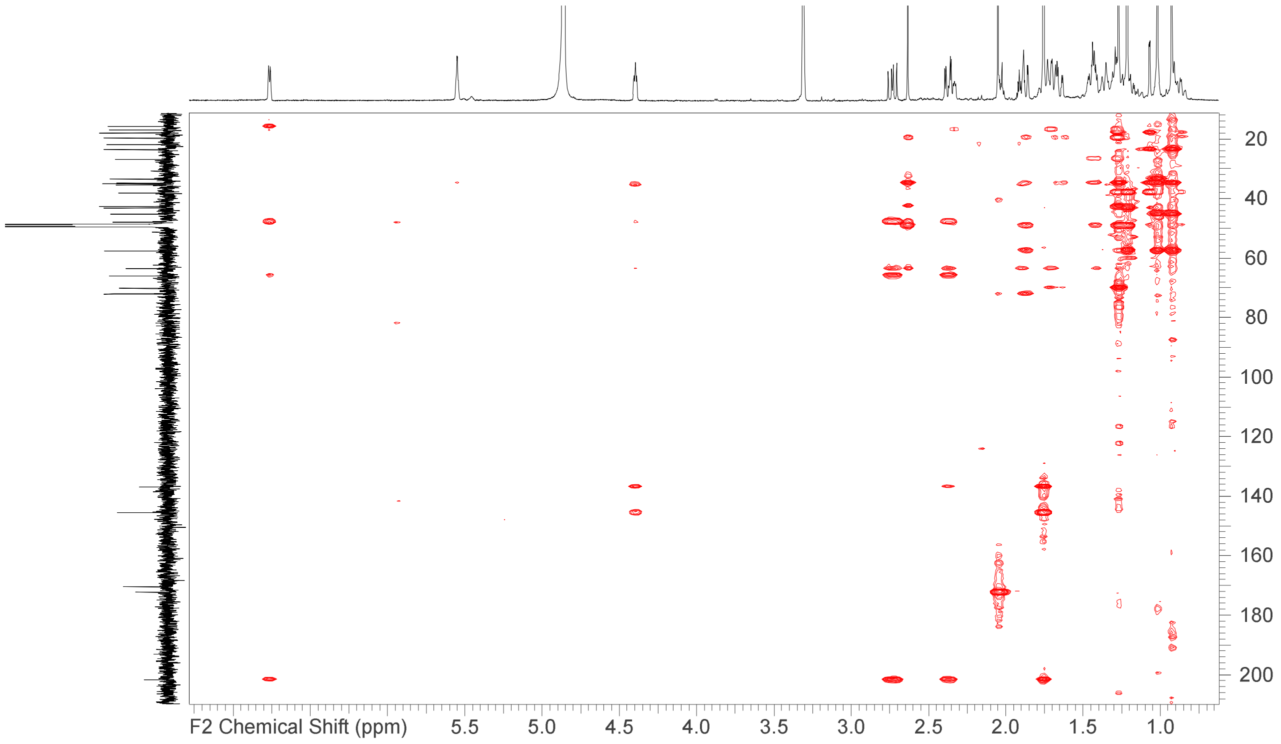

Figure S13 – NOESY NMR spectrum (500 MHz, CD_3OD) of **2**

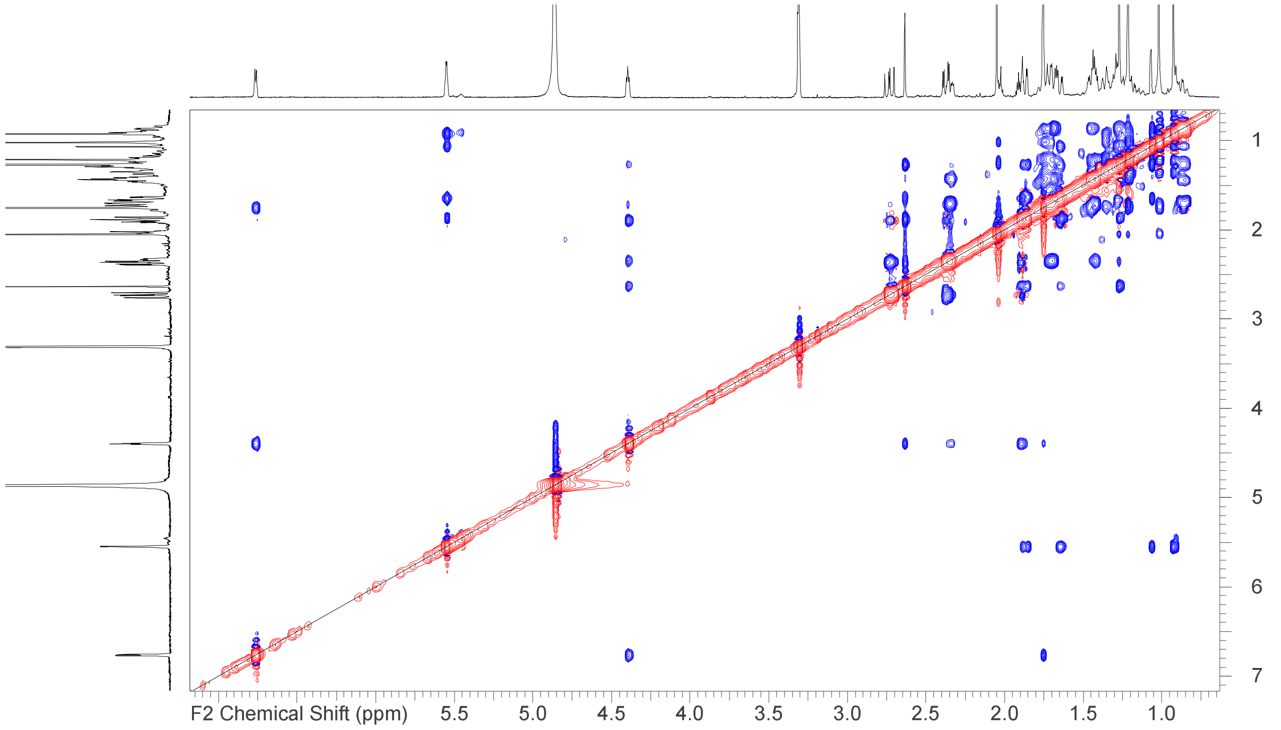

Figure S14 – HRESIMS analysis of **2**

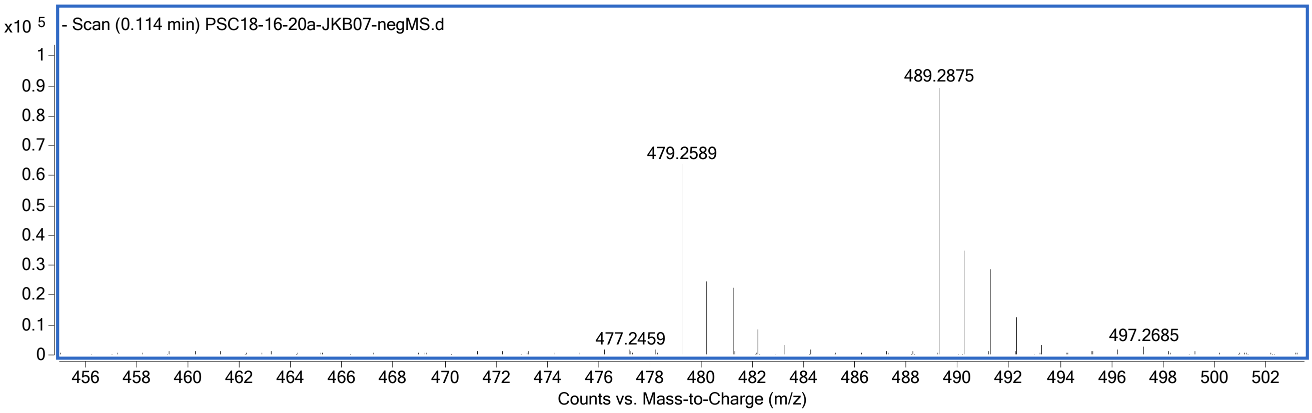

		C27 H40 O5	72.3	444.29012			-5.72	5.72	-2.54	0.1[illegible]

Species	Io	m/z	Height	Score (MFG)	Score (MS)	Score (mass)	Score (iso. abund)	Score (iso. spacing)
(M-H)-		443.281	913.2		72.3	95.09	31.13	76.12
(M+Cl)-		479.2578	32198.2		71.88	69.79	76.99	69.92
(M+HCOO)-		489.2865	52077.2		52.82	78.14	2.46	62.61

m/z	m/z (Calc)	Diff (ppm)	Diff (mDa)	Height	Height (Calc)	Height %	Height % (Calc)	Height Sum %	Heig[illegible]
489.28...	489.2858	-1.58	-0.8	41853.9	52077.2	100	100	58.6	
490.29...	490.2892	-2.31	-1.1	16103.4	16155.5	38.5	31	22.6	
491.30...	491.292	-17.78	-8.7	13445.1	3169.7	32.1	6.1	18.8	

Table S3 – NMR data for suberitenone F (**3**) (600 (^{1}H) and 150 (^{13}C) MHz, CD_3OD).

pos	δ_C, type	δ_H	gCOSY	gHMBC	NOESY
1	66.0, CH	4.30, dd (5.6, 3.3)	2, 6, 21	2, 3, 5	8, 5', 22, 23
2	145.8, CH	6.85, dq (5.5, 1.4)	1, 21	1, 4, 6, 21	5, 6
3	136.9, C				
4	202.6, C				
5	38.5, CH_2	2.83, dd (15.9, 12.8)	5', 6	1, 4, 6	2, 8, 22
5'		2.31, (16.1, 2.8)	5, 6	1, 3, 4, 6	1, 8, 22
6	45.4, CH	2.90, m	1, 5, 5'	1, 4, 7	2, 8, 22
7	137.2, C				
8	128.9, CH	5.67, t (3.2)	9	6, 9, 10, 22	1, 5, 5', 6, 10, 23
9	24.1, CH_2	2.12, o/l	8, 10	7, 8, 10	23, 24
10	46.8, CH	1.60, dd (11.3, 5.9)	9	9, 11, 15, 16, 22, 23, 24	8, 12, 14, 16'
11	38.0, C				
12	39.8, CH_2	2.16, o/l	12', 13	23, 11, 10, 22,	10, 14, 16, 22
12'		1.53, dd (15.0, 2.7)	12, 13	10, 11, 13, 14, 23	22, 23
13	72.3, CH	5.61, dt (4.4, 2.4)	12, 12', 14	11, 15	20
14	57.2, CH	1.13, d (2.1)	13	10, 12, 15, 16, 18, 19, 20, 24, 25	10, 12, 16', 18', 20
15	38.0, C				
16	43.5, CH_2	1.72, o/l	16', 17, 17'	14, 18	9, 10, 24
16'		0.99, o/l	16, 17, 17'		
17	19.6, CH_2	1.73, o/l	16, 16', 17', 18, 18'		24, 25
17'		1.46, m	16, 16', 17', 18, 18		
18	45.1, CH_2	1.39, m	17, 17', 18'		20, 25
18'		1.24, td (13.1, 3.9)	17, 17', 18	19, 25	20
19	34.8, C				
20	33.8, CH_3	0.96, s		14, 18, 19, 25	13, 14, 18, 18', 25
21	15.6, CH_3	1.78, s	1, 2	2, 3, 4	
22	75.3, CH	3.15, s		6, 7, 8, 10, 11, 12, 23	1, 5, 5', 6, 12, 12', 23
23	20.8, CH_3	0.99, s		10, 11, 12, 22	1, 8, 9, 22, 24, 27
24	17.6, CH_3	1.34, s		10, 14, 15, 16	9, 16, 17, 23, 25
25	23.8, CH_3	1.04, s		14, 18, 19, 20	17, 18, 20, 24, 27
26	172.4, C				
27	21.9, CH_3	2.04, s		26	23, 24, 25

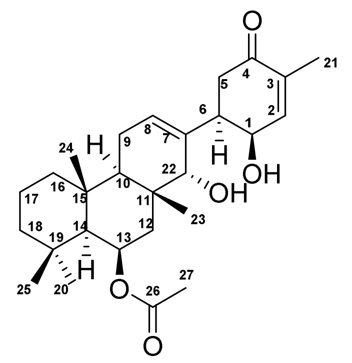

Figure S15 – ^{1}H NMR spectrum (600 MHz, CD_3OD) of **3**

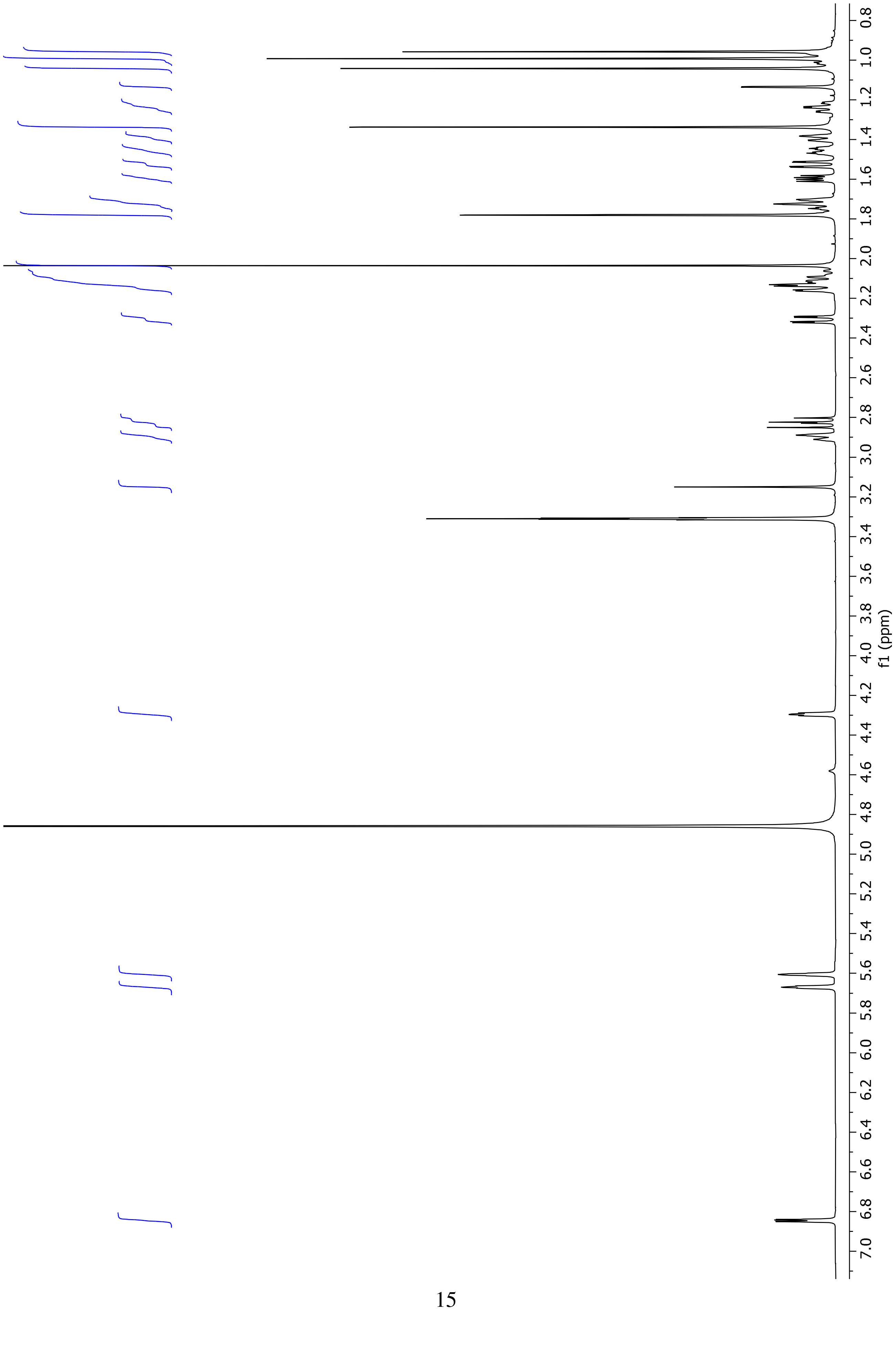

Figure S16 – ^{13}C NMR spectrum (150 MHz, CD_3OD) of **3**

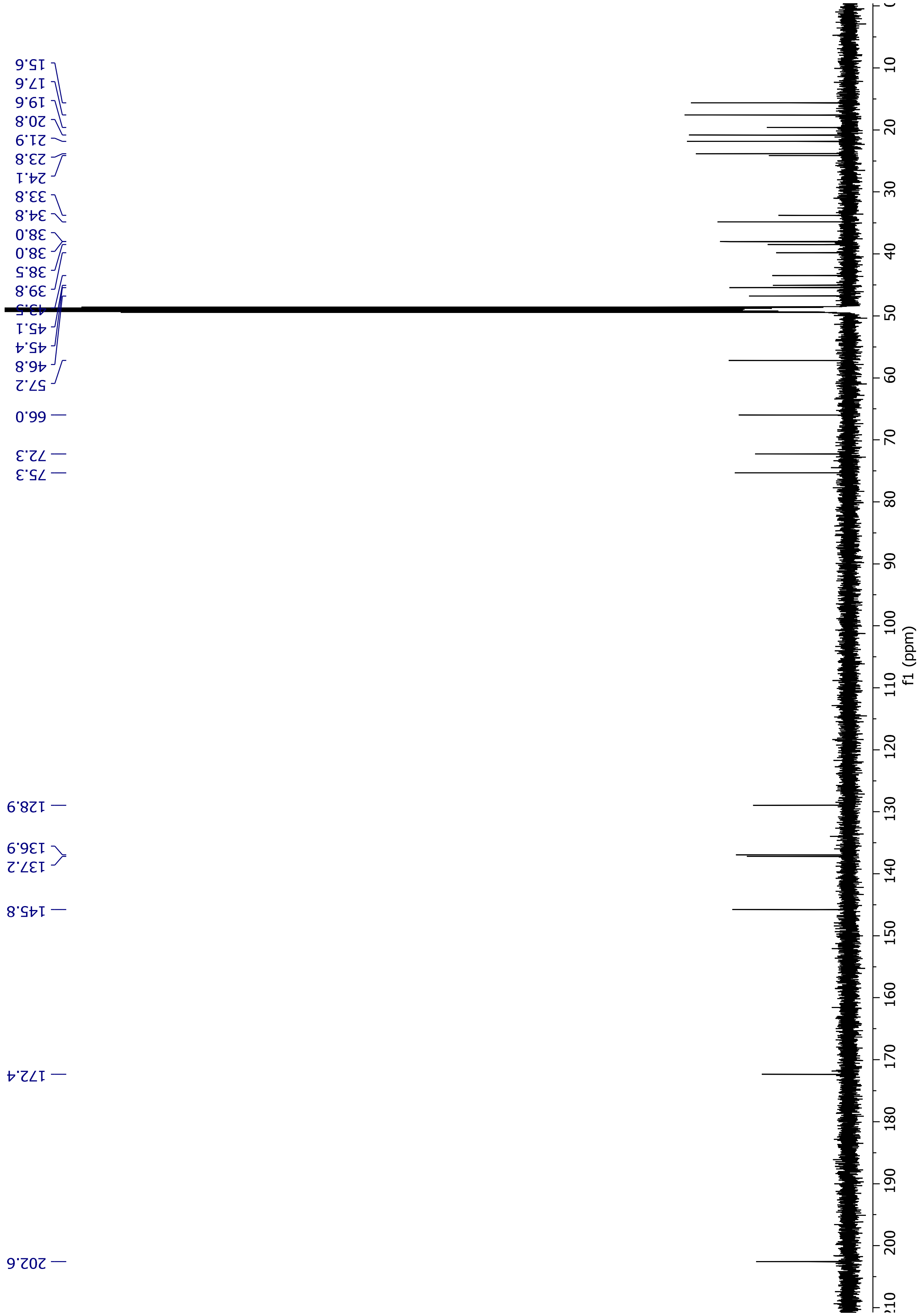

Figure S17 – COSY NMR spectrum (600 MHz, CD3OD) of **3**

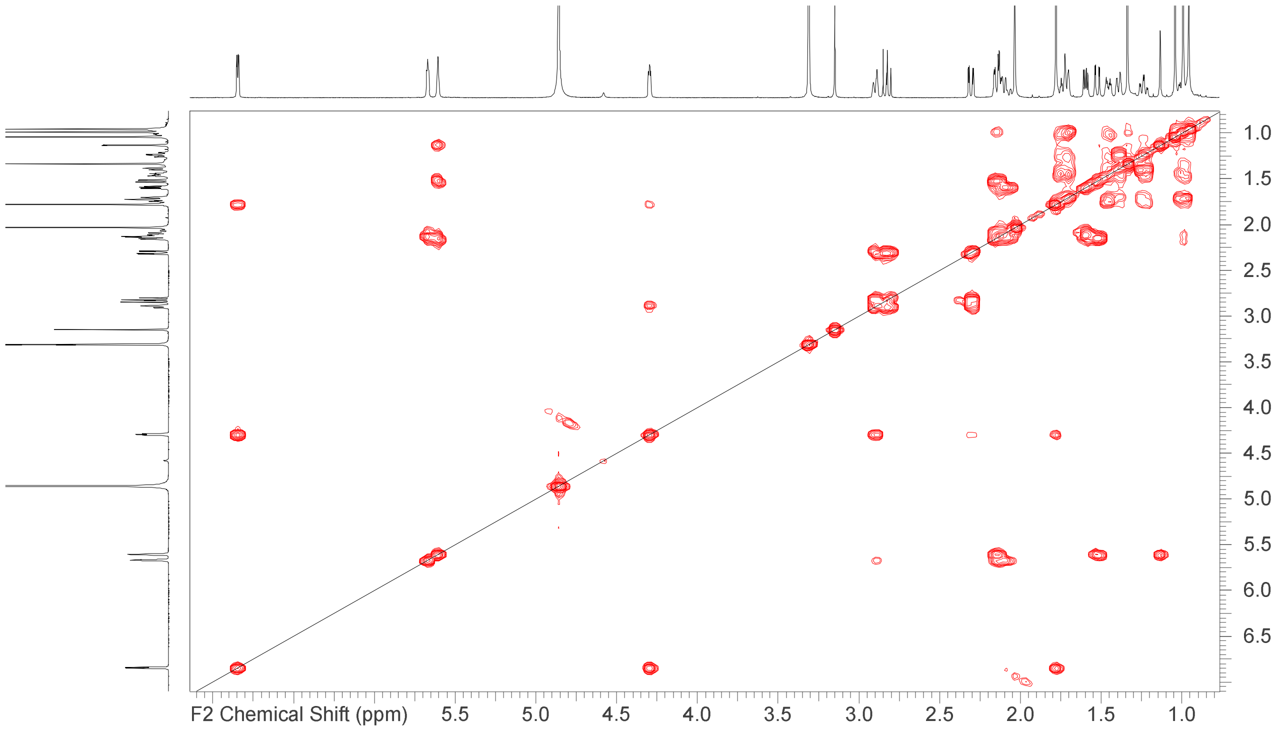

Figure S18 – HSQC NMR spectrum (600 MHz, CD3OD) of **3**

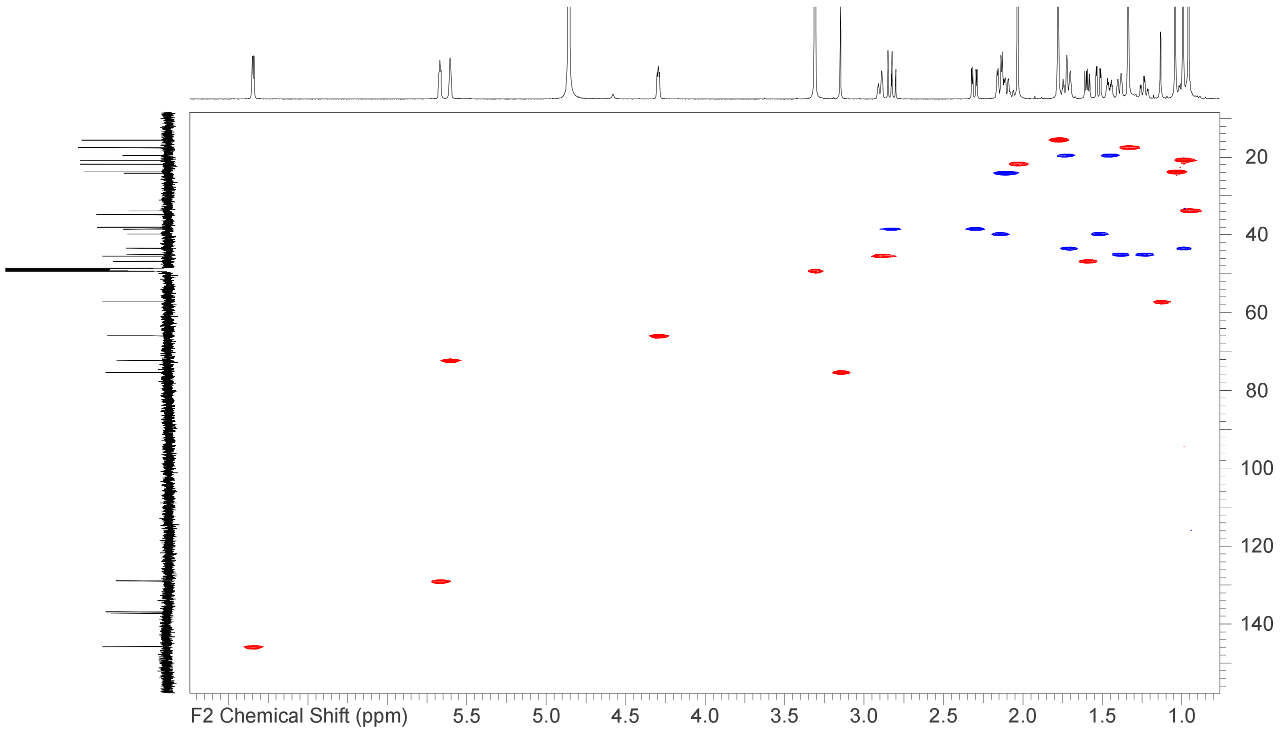

Figure S19 – HMBC NMR spectrum (500 MHz, CD_3OD) of **3**

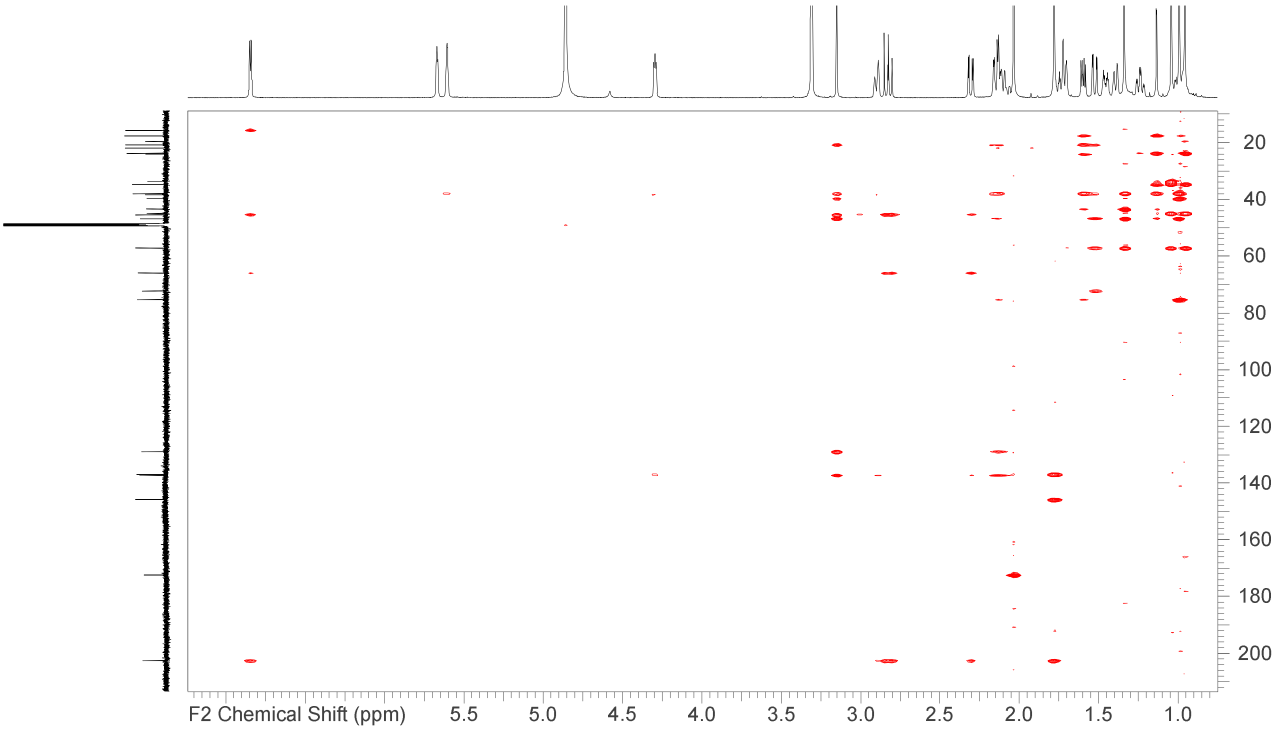

Figure S20 – NOESY NMR spectrum (600 MHz, CD_3OD) of **3**

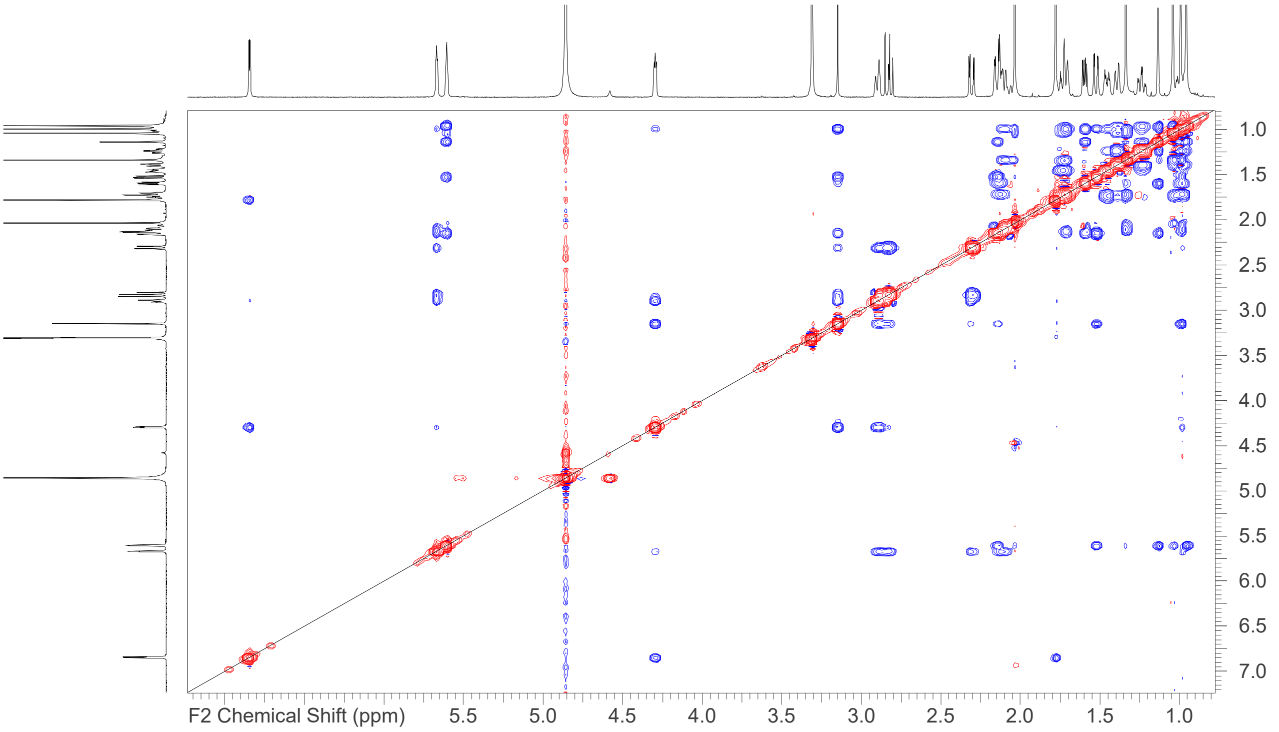

Figure S21 – HRESIMS analysis of **3**

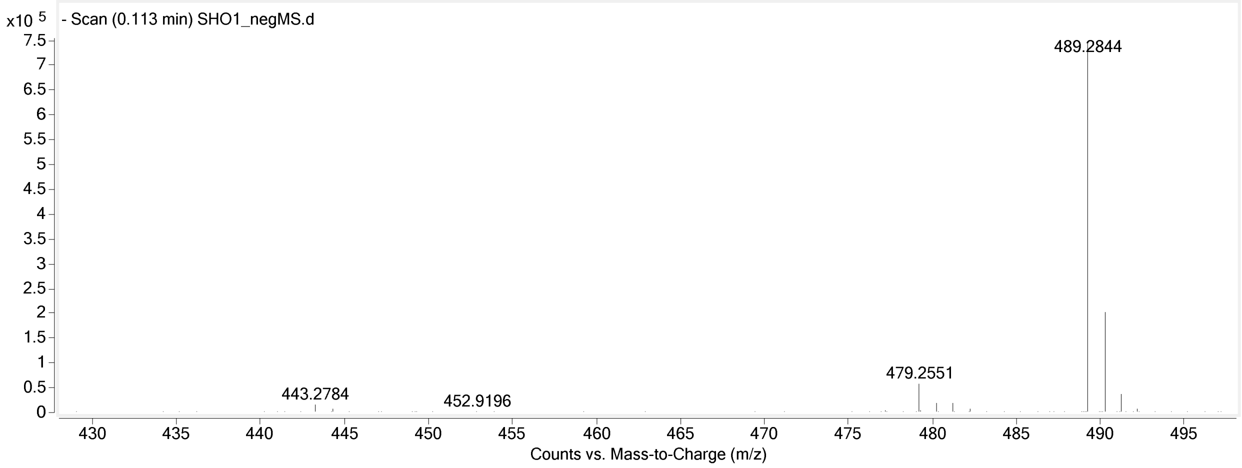

Best	Name	Formula	Score	Mass	Mass (DB)	Mass (MFG)	Diff (ppm)
⦿		C27 H40 O5	94.28	444.28592			3.73

Species	Ion Formula	m/z	Height	Score (MFG)	Score (MS)	Score (mass)	S
(M-H)-		443.2788	3357.4		84.82	88.61	
(M+Cl)-		479.2553	7024.7		79.29	87.03	
(M+HCOO)-		489.2841	144546.5		94.28	88.52	

m/z	m/z (Calc)	Diff (ppm)	Diff (mDa)	Height	Height (Calc)	Height %	He
489.2841	489.2858	3.34	1.6	144546.5	142738.5	100	
490.2874	490.2892	3.57	1.7	43854.3	44280.7	30.3	
491.2904	491.292	3.32	1.6	7405.7	8687.8	5.1	
492.2938	492.2947	1.86	0.9	1216.8	1275.6	0.8	
493.2936	493.2974	7.7	3.8	111.7	152.4	0.1	

Table S4 – NMR data for suberitenone G (**4**) (500 (^{1}H) and 150 (^{13}C) MHz, CD_3OD).

**pos**	δ_C, type	δ_H	gCOSY	gHMBC	NOESY
**1**	64.7, CH	4.14, br t (4.2)	2, 6	3, 5	5, 5', 23
**2**	145.5, CH	6.80, dq (1.5, 5.6)	1, 21	1, 4, 6, 21	6
**3**	136.8, C				
**4**	202.2, C				
**5**	37.7, CH_2	2.82, dd (16.2, 13.4)	5', 6	1, 4, 6	1, 8
**5'**		2.18, dd (16.2, 3.6)	5, 6	1, 4, 6, 7	1, 8
**6**	38.8, CH	3.39, m	1, 5, 5'		2
**7**	135.7, C				
**8**	147.2, CH	6.78, m	9	6, 10	5, 5'
**9**	24.6, CH_2	2.52, m	8, 10	7, 8, 10, 11	16', 23, 24
**10**	54.7, CH	1.71, o/l	9	9, 11, 12, 16, 22, 23, 24	14, 16'
**11**	45.7, C				
**12**	39.4, CH_2	2.13, dd (15.5, 2.7)	12', 13	10, 11, 13, 14, 23	23
**12'**		1.69, o/l	12, 13	11, 22, 23	14
**13**	71.3, CH	5.61, q (3.2)	12, 12', 14		20
**14**	56.5, CH	1.13, m	13	10, 15, 16, 18, 19, 20, 24, 25	10, 12', 20
**15**	39.0, C				
**16**	42.6, CH_2	1.76, o/l	16', 17, 17'	14, 18, 24	
**16'**		0.98, m	16, 17, 17'		9, 10
**17**	19.4, CH_2	1.77, o/l	16, 16', 18, 18'	16, 18	
**17'**		1.49, m	16, 16', 17, 18, 18'		
**18**	44.9, CH_2	1.40, m	17, 17', 18'		
**18'**		1.24, m	17, 17', 18		
**19**	34.8, C				
**20**	33.5, CH_3	0.95, s		14, 18, 19, 25	13, 14
**21**	15.6, CH_3	1.79, s	2	2, 3, 4	
**22**	205.5, C				
**23**	19.6, CH_3	1.27, s		10, 11, 12, 22	1, 9, 12, 24, 27
**24**	18.1, CH_3	1.42, s		10, 14, 15, 16	9, 23, 25, 27
**25**	23.7, CH_3	1.04, s		14, 18, 19, 20	24, 27
**26**	172.0, C				
**27**	21.8, CH_3	2.04, s		26	23, 24, 25

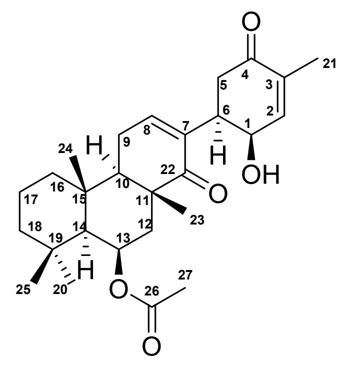

Figure S22 – ^{1}H NMR spectrum (500 MHz, CD_3OD) of **4**

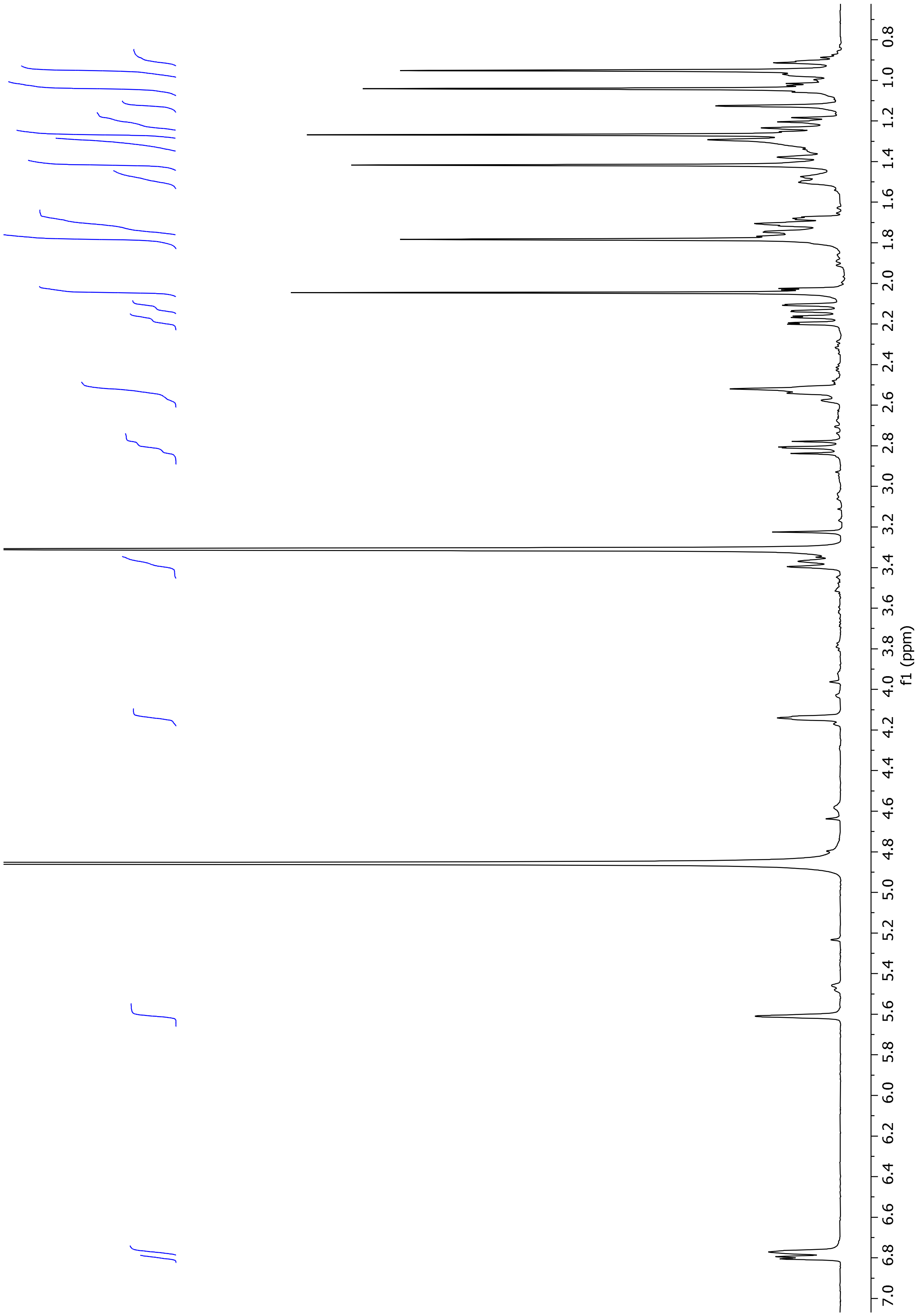

Figure S23 – ^{13}C NMR spectrum (150 MHz, CD_3OD) of **4** (formic acid impurity δ_C 170.3)

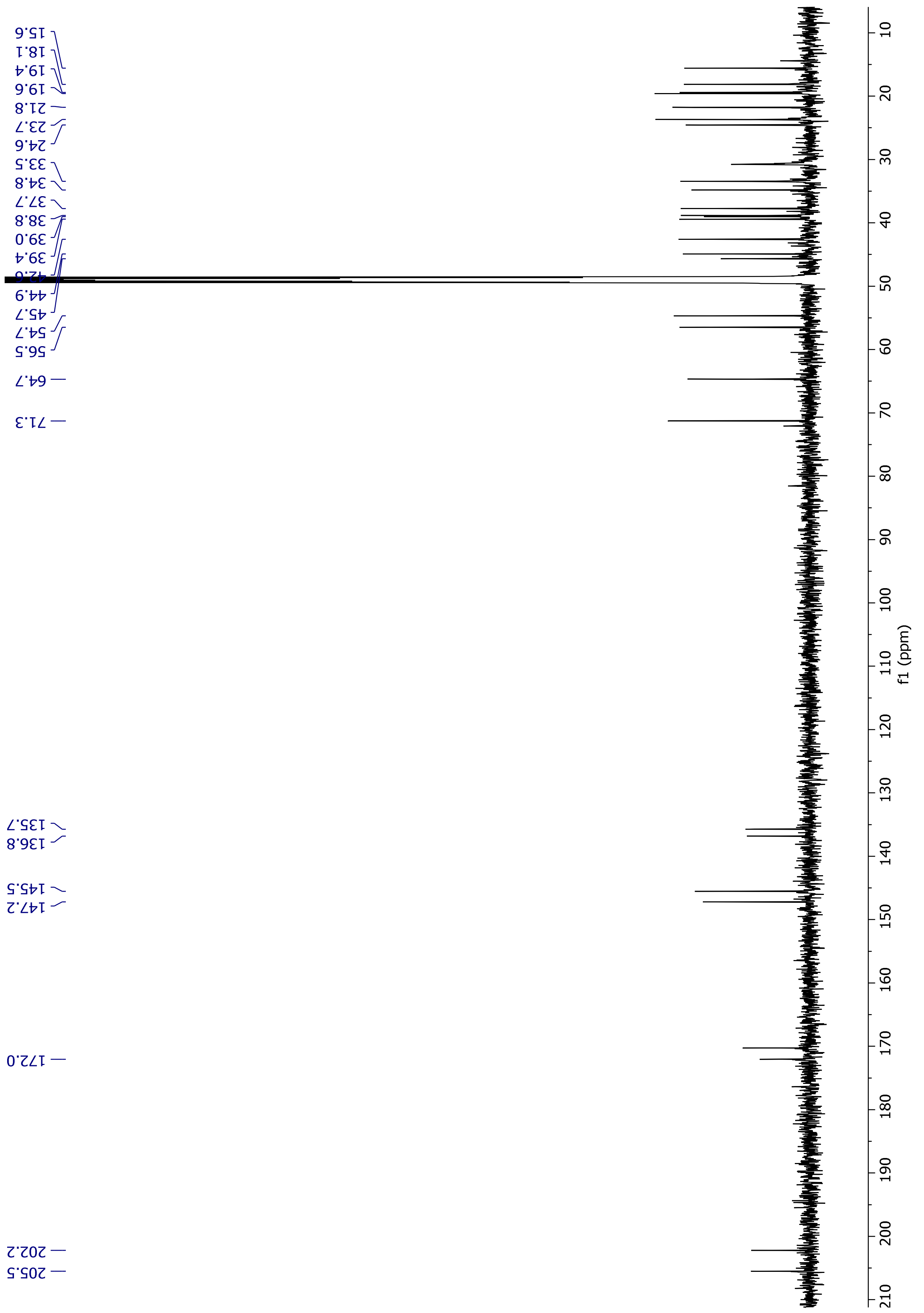

Figure S24 – COSY NMR spectrum (500 MHz, CD_3OD) of **4**

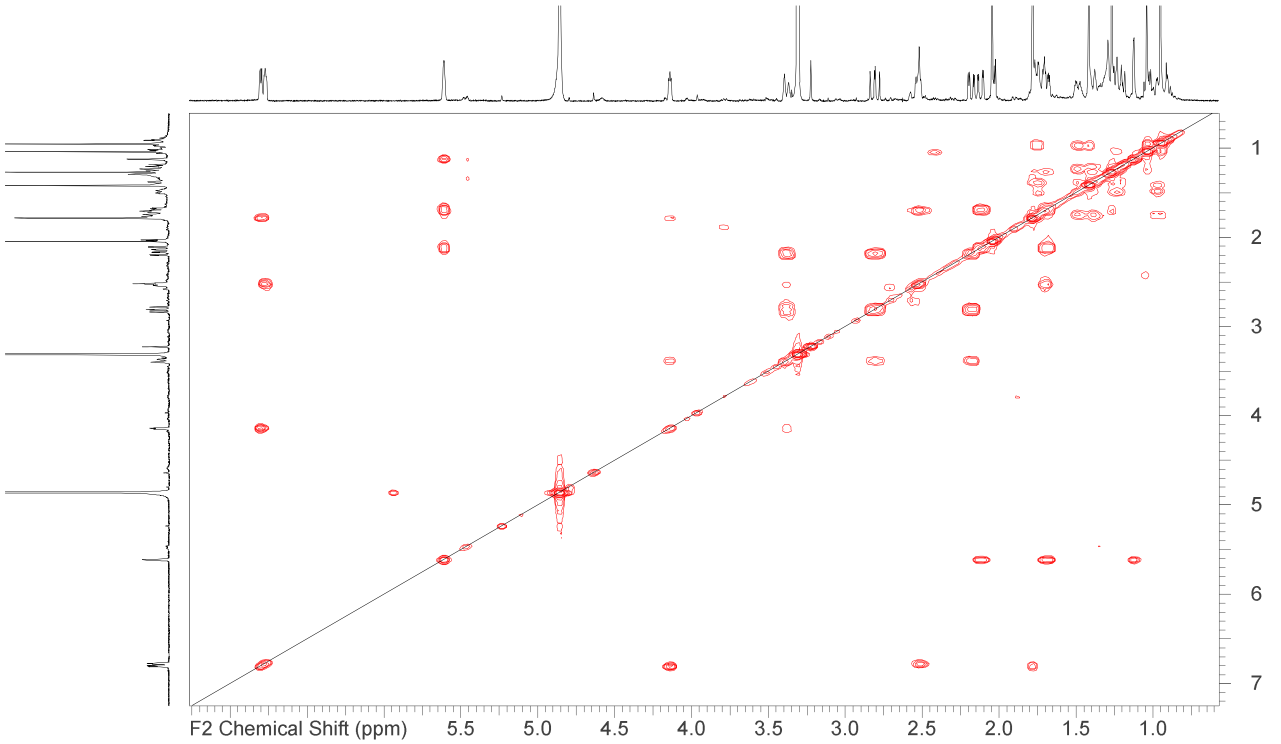

Figure S25 – HSQC NMR spectrum (500 MHz, CD_3OD) of **4**

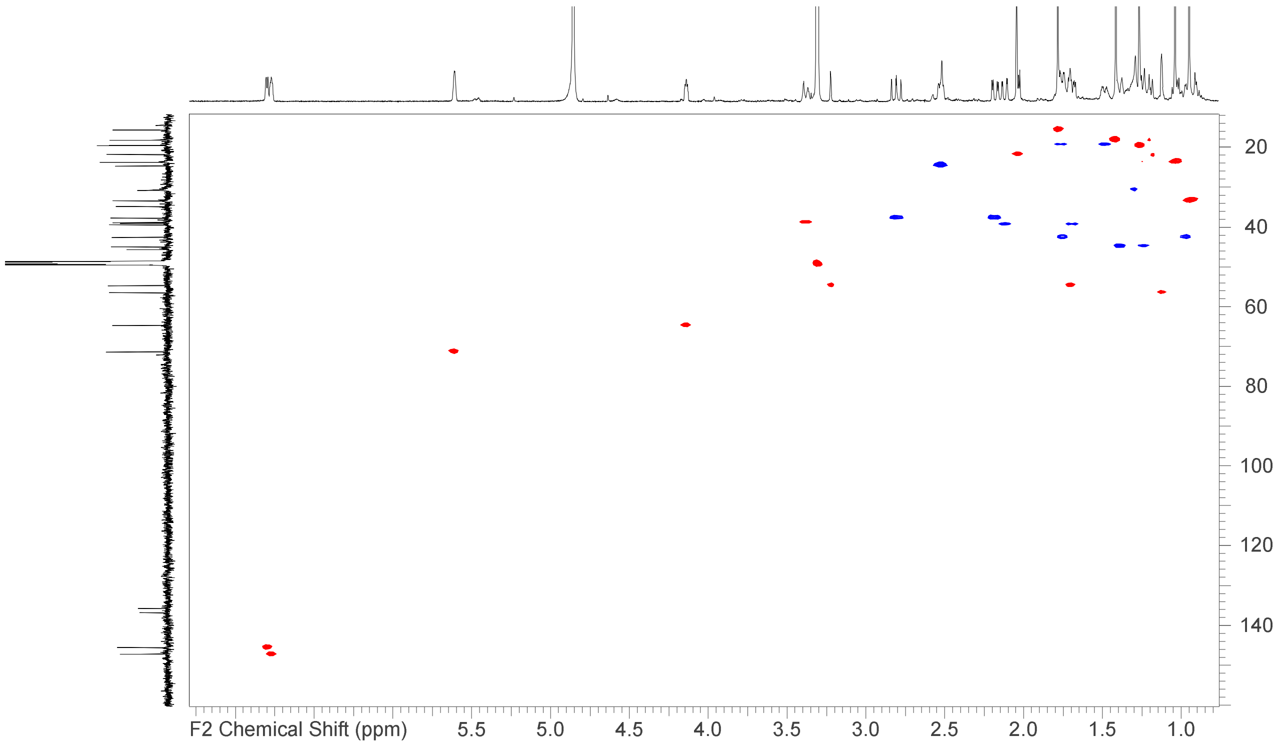

Figure S26 – HMBC NMR spectrum (500 MHz, CD_3OD) of **4**

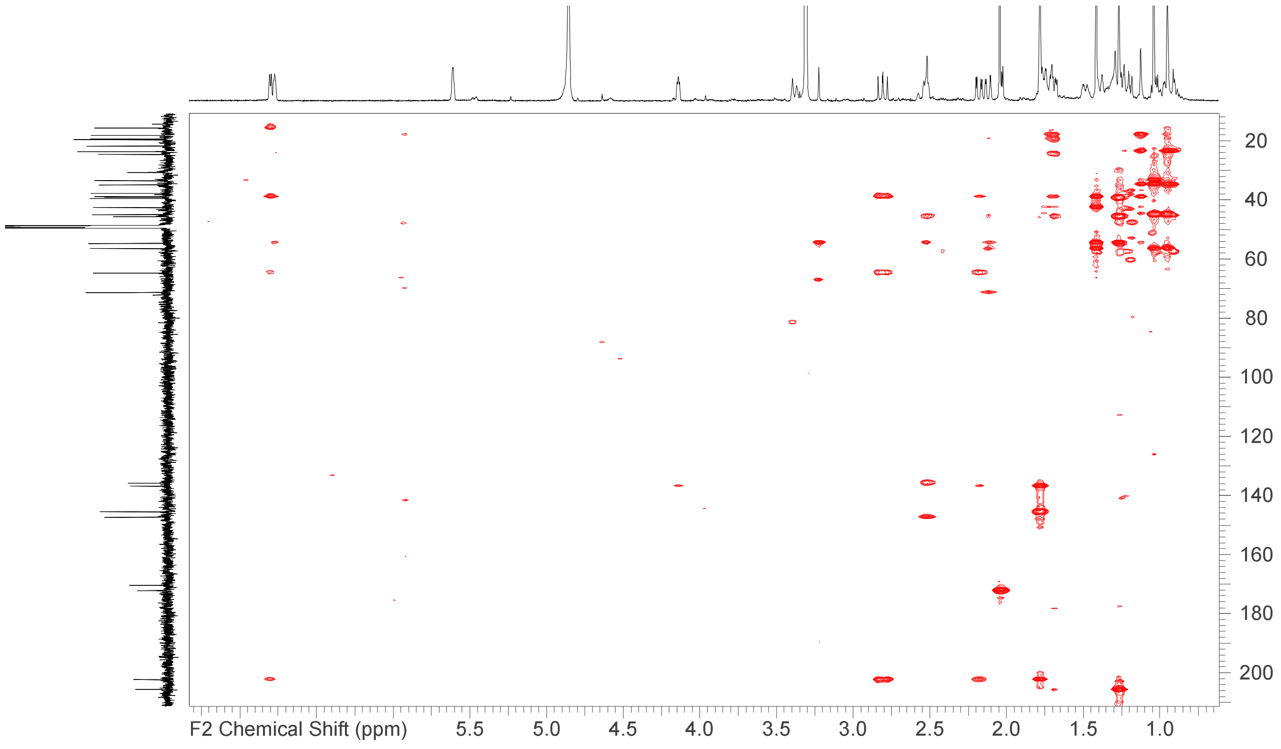

Figure S27 – NOESY NMR spectrum (600 MHz, CD_3OD) of **4**

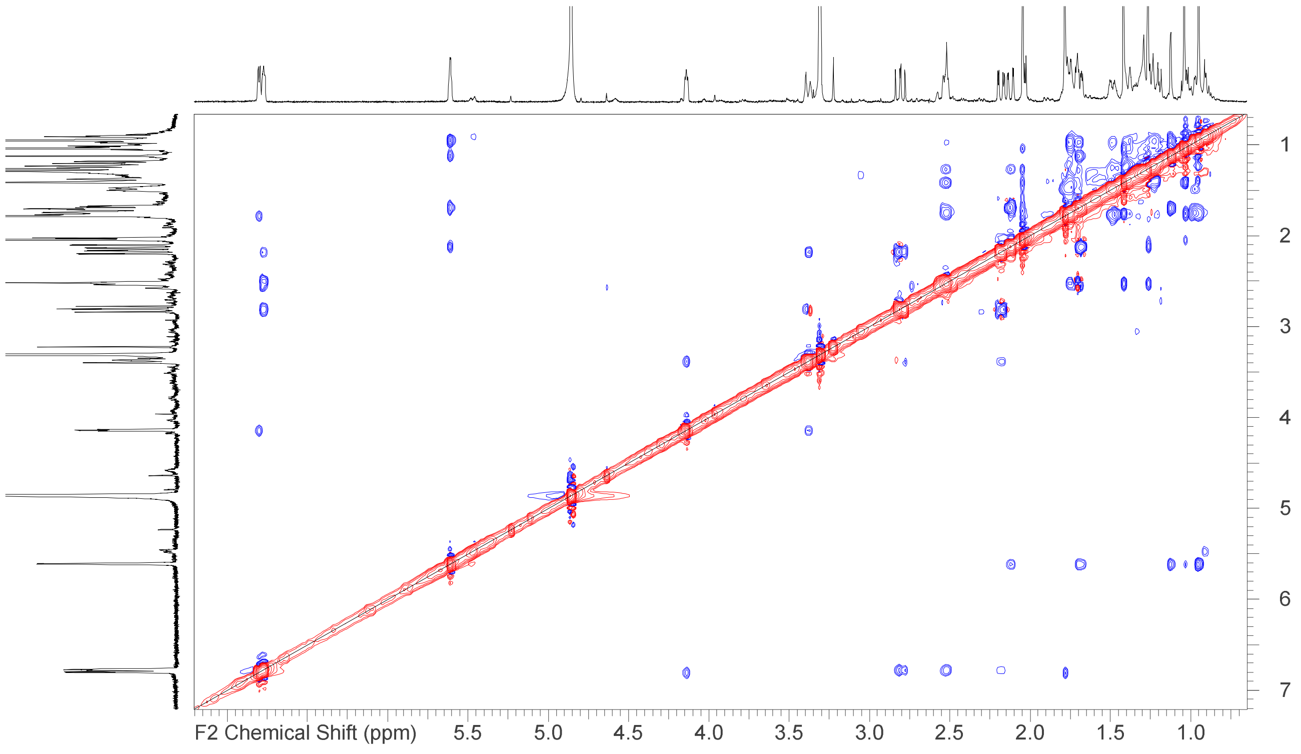

Figure S28 – HRESIMS analysis of **4**

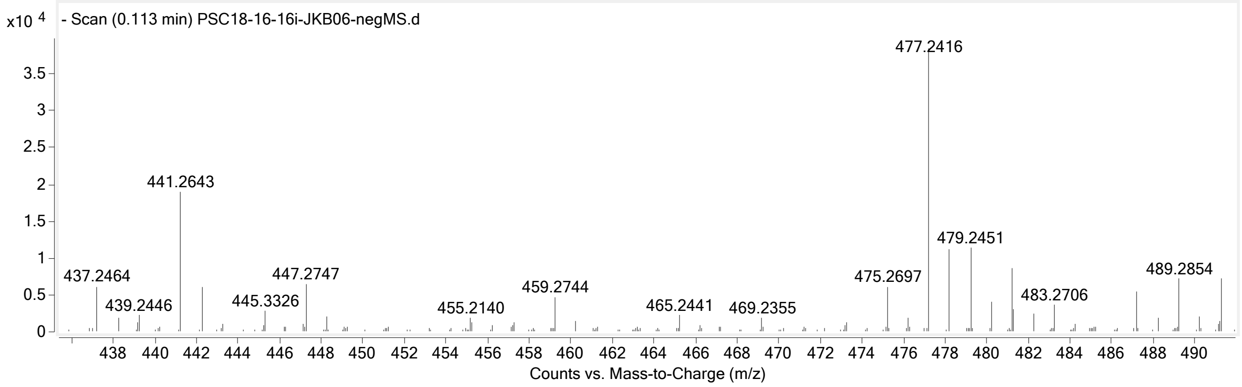

☑		C27 H38 O5	98.5	442.27288			-2.16	2.16	-0.96	0.113

Species	Ion Formula	m/z	Height	Score (MFG)	Score (MS)	Score (mass)	Score (iso. abund)	Score (iso. spacing)
(M-H)-		441.2645	11993.2		98.5	99.95	95.43	99.29

m/z	m/z (Calc)	Diff (ppm)	Diff (mDa)	Height	Height (Calc)	Height %	Height % (Calc)	Height Sum %	Height Su
441.2645	441.2646	0.42	0.2	11993.2	11835.6	100	100	74.6	
442.268	442.268	0.13	0.1	3284.9	3529.2	27.4	29.8	20.4	
443.2718	443.271	-1.81	-0.8	724.4	629.1	6	5.3	4.5	
444.2767	444.2737	-6.68	-3	74.5	83.1	0.6	0.7	0.5	

Species	Ion Formula	m/z	Height	Score (MFG)	Score (MS)	Score (mass)	Score (iso. abund)	Score (iso. spacing)
(M+Cl)-		477.2413	14947.5		92.43	99.81	78.6	94.25
(M+HCOO)-		489.2853	7007.3		30.73	35.69	0	57.7

Table S5 – NMR data for suberitenone H (**5**) (500 (^{1}H) and 150 (^{13}C) MHz, CD_3OD).

pos	δ_C, type	δ_H	gCOSY	gHMBC	NOESY
1	66.6, CH	4.46, br t (3.3)	2, 6	2, 3, 5	22', 28
2	88.6, CH	3.52, t (3.6)	1, 3	1, 3, 4, 6, 21, 28	21, 28
3	44.6, CH	3.03, m	2, 21	2, 4, 21	5
4	214.6, C				
5	37.4, CH_2	2.65, t (13.7)	5', 6	1, 4, 6, 7	3
5'		2.23, dd (4.7, 13.8)	5, 6	1, 3, 4, 6	8, 8'
6	48.3, CH	1.81, o/l	1, 5, 5', 8	5, 7, 8	8', 28
7	75.1, C				
8	38.7, CH_2	1.91, o/l	6, 8', 9, 9'	10, 22	5'
8'		1.21, o/l	8, 9, 9'		5', 6
9	18.0, CH_2	1.66, td (12.8, 3.4)	8, 8', 9', 10	10, 11, 15	
9'		1.57, m	8, 8', 9, 10	10, 11, 15	16
10	59.7, CH	0.99, dd (12.3, 2.5)	9, 9'		12', 14, 22'
11	35.5, C				
12	48.1, CH_2	1.88, o/l	12', 13	10, 11, 13, 14, 22, 23	
12'		1.32, m	12, 13		10, 14
13	72.3, CH	5.48, br q (3.2)	12, 12', 14	11, 15	20
14	57.7, CH	1.14, br s	13	10, 12, 15, 16, 18, 19, 20, 24, 25	10, 12', 16'
15	38.3, C				
16	43.0, CH_2	1.77 ,o/l	16', 17, 17'	10, 14, 15, 24	9'
16'		0.92, o/l	16, 17, 17'		14
17	19.7, CH_2	1.77, o/l	16, 16', 17', 18, 18'	15	24, 25
17'		1.47, m	16, 16', 17, 18, 18'		
18	45.4, CH_2	1.37, o/l	17, 17', 18'		20, 25
18'		1.23, o/l	17, 17', 18		
19	35.0, C				
20	33.3, CH_3	0.91, s		14, 18, 19, 25	13, 18
21	10.4, CH_3	1.02, d (6.8)	3	2, 3, 4	2, 28
22	55.3, CH_2	1.81, o/l		7, 8, 10, 11, 23	23
22		1.15/ o/l			1, 10
23	23.4, CH_3	1.37, s		10, 11, 12, 22	22, 25, 27
24	17.9, CH_3	1.23, s		10, 14, 15, 16	17, 25, 27
25	23.7, CH_3	1.04, s		14, 18, 19, 20	17, 18, 23, 24, 27
26	172.3, C				
27	21.8, CH_3	2.03, s		26	23, 24, 25
28	59.1, CH_3	3.35, s		2	1, 2, 6, 21

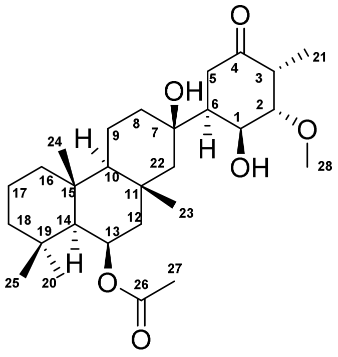

Figure S29 – ^{1}H NMR spectrum (500 MHz, CD_3OD) of **5**

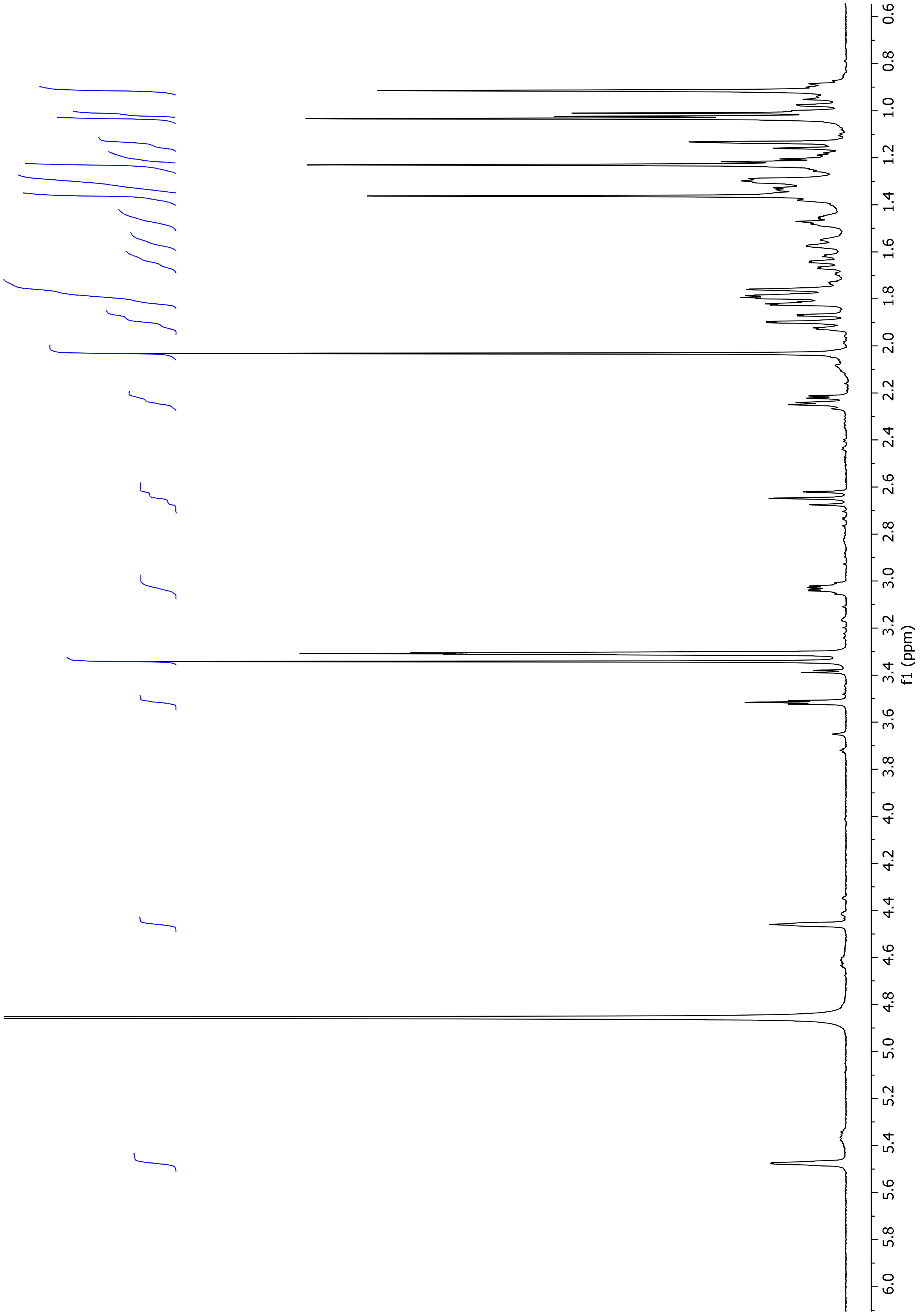

Figure S30 – ^{13}C NMR spectrum (150 MHz, CD_3OD) of **5**

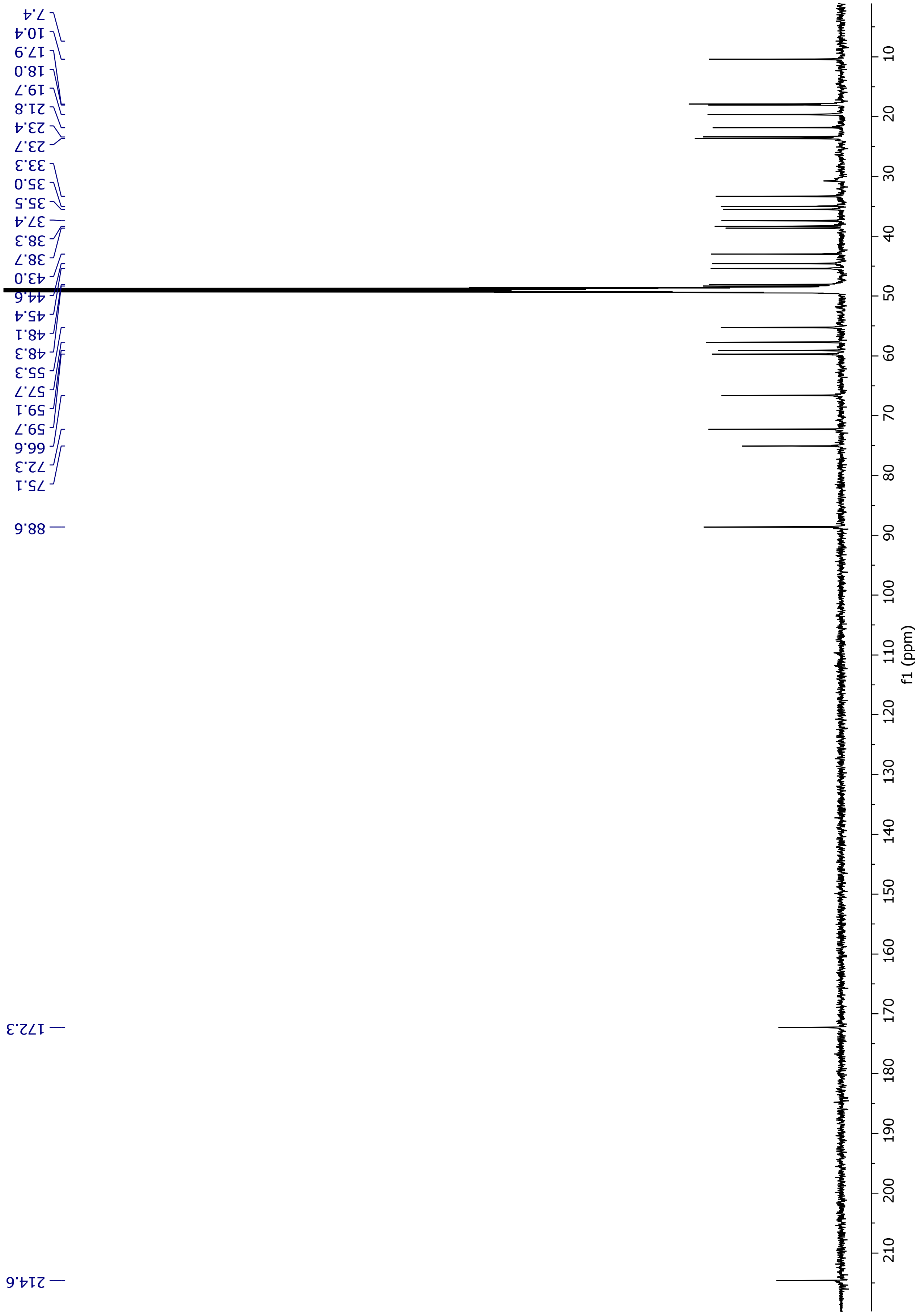

Figure S31 – COSY NMR spectrum (500 MHz, CD_3OD) of **5**

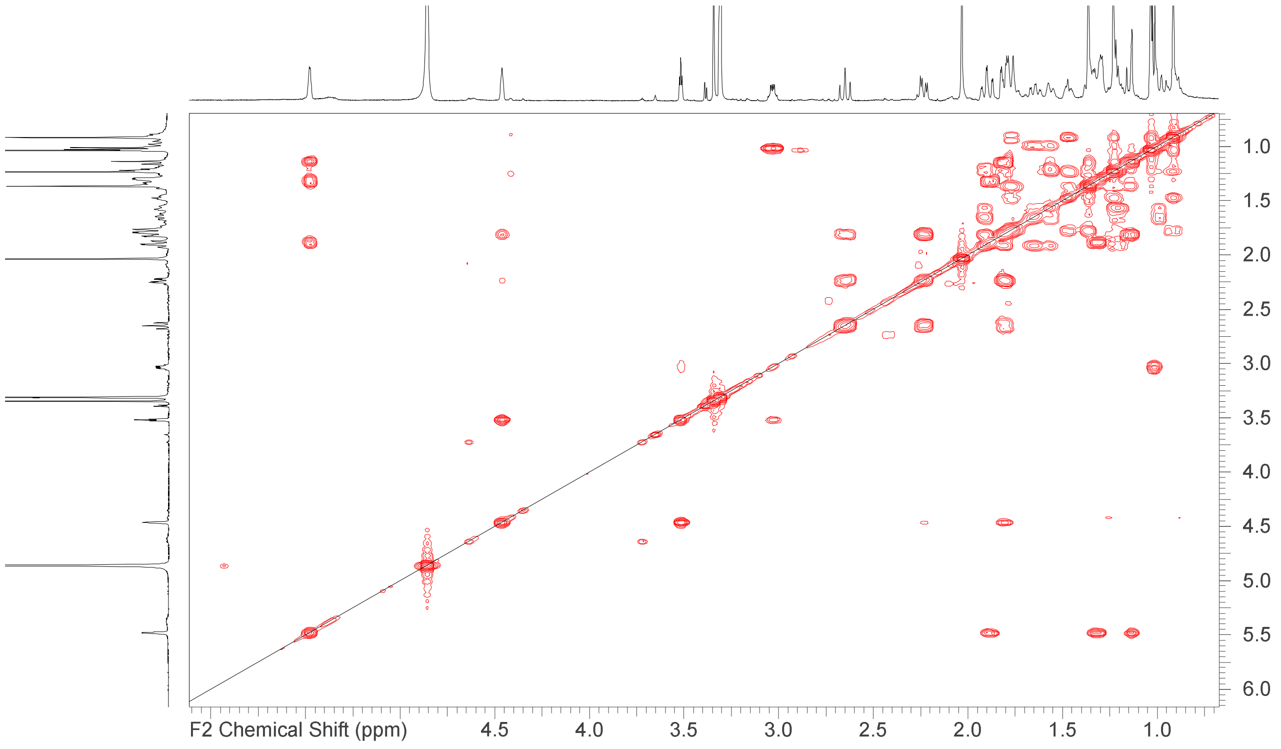

Figure S32 – HSQC NMR spectrum (500 MHz, CD_3OD) of **5**

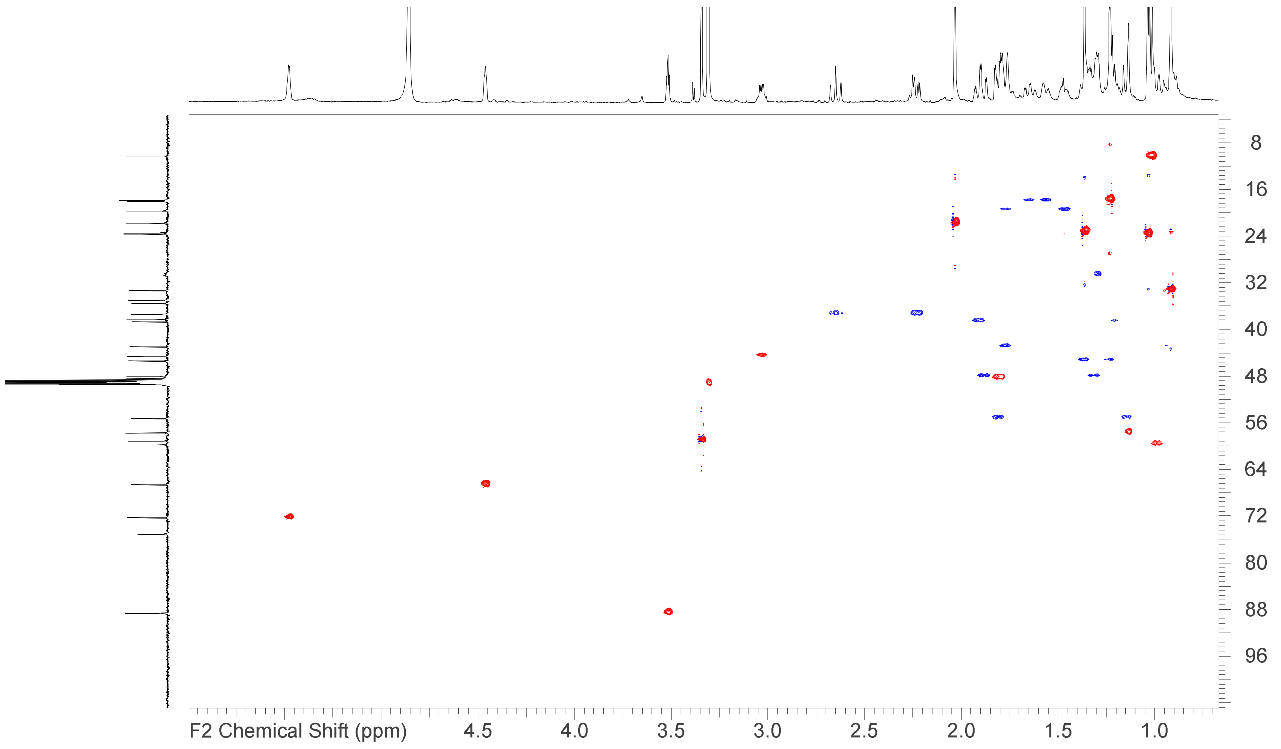

Figure S33 – HMBC NMR spectrum (500 MHz, CD_3OD) of **5**

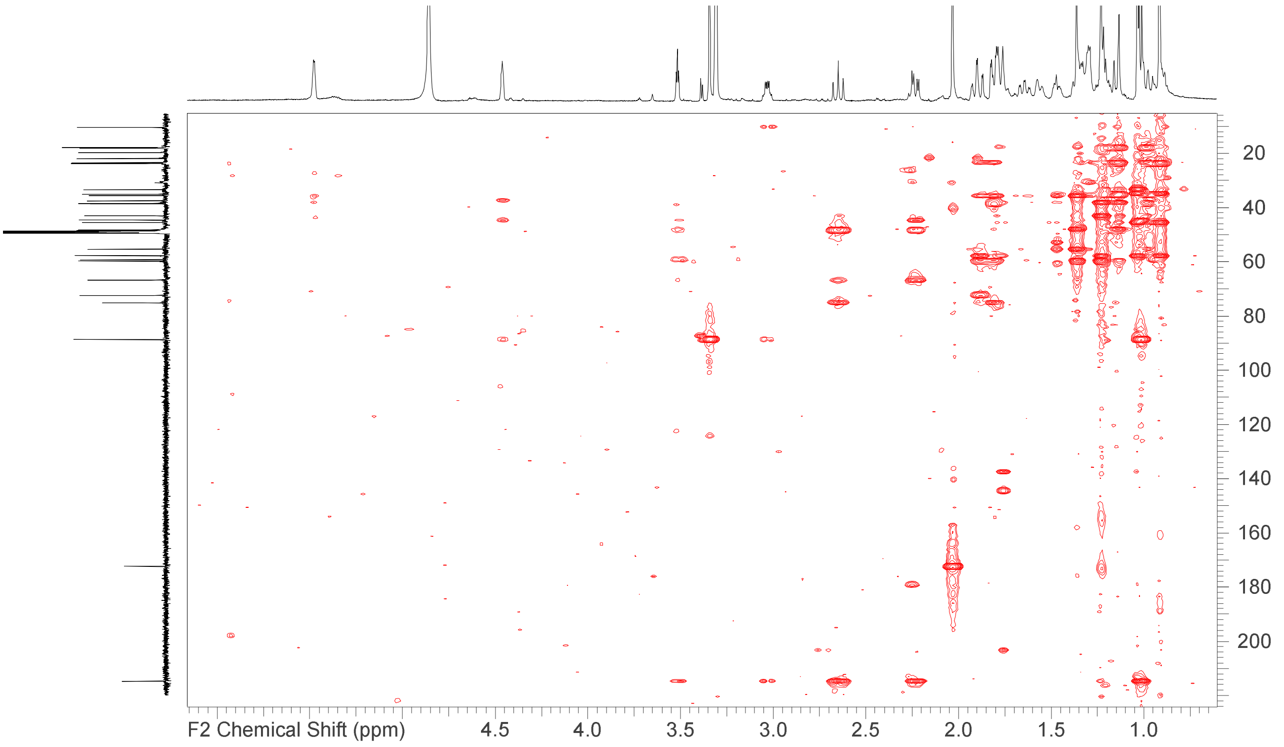

Figure S34 – NOESY NMR spectrum (600 MHz, CD_3OD) of **5**

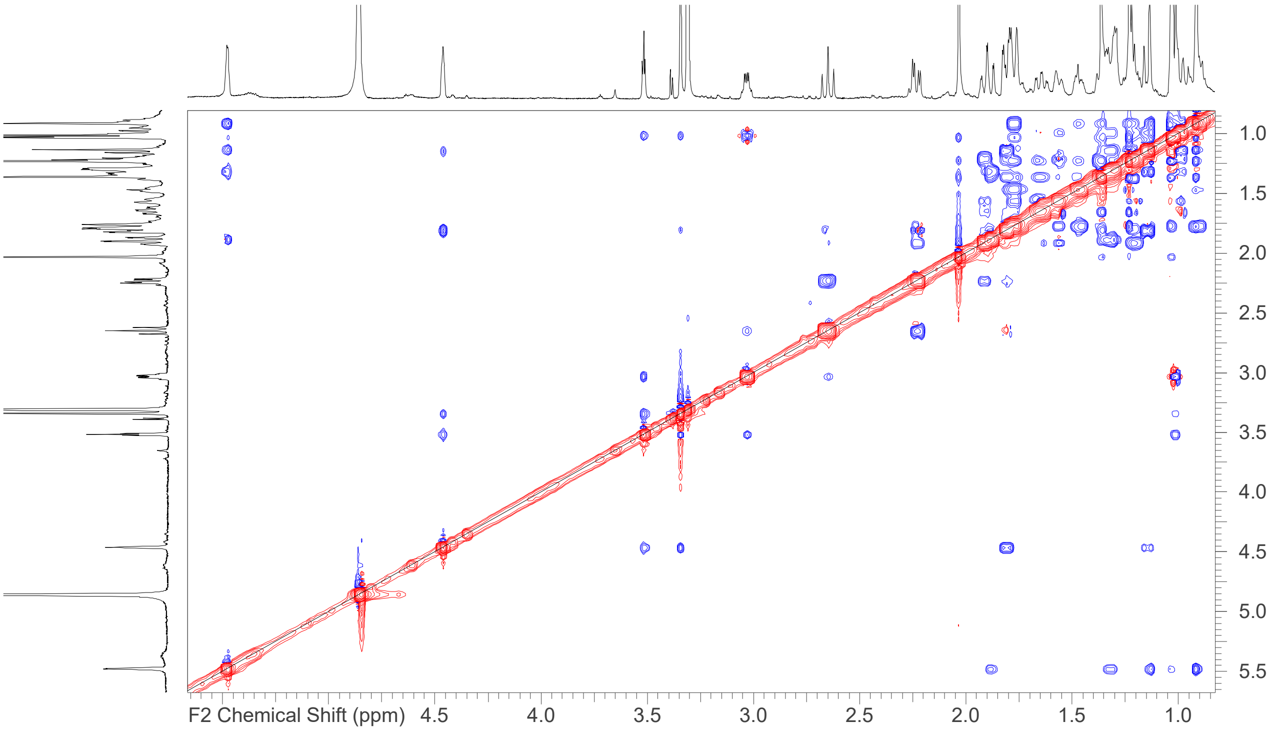

Figure S35 – HRESIMS analysis of **5**

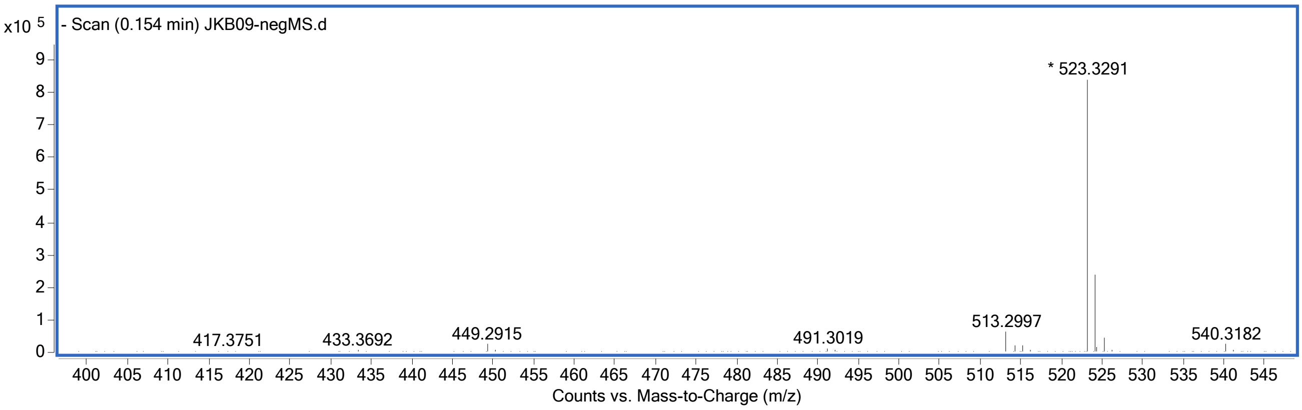

		C28 H46 O6	97.96	478.33007			-1.31	1.31	-0.63	0.104

Species	Ion Formula	m/z	Height	Score (MFG)	Score (MS)	Score (mass)	Score (iso. abund)	Score (iso. spacing)
(M-H)-		477.3236	99.2		43.19	90.7	0	0
(M+Cl)-		513.2994	19748.1		97.96	99.21	94.42	99.68
(M+HCOO)-		523.3284	148290.3		97.53	98.22	94.6	99.66

m/z	m/z (Calc)	Diff (ppm)	Diff (mDa)	Height	Height (Calc)	Height %	Height % (Calc)	Height Sum %	Height Su
523.3284	523.3276	-1.36	-0.7	148290.3	143728.5	100	100	73.7	
524.3315	524.3311	-0.78	-0.4	42810.1	46296.3	28.9	32.2	21.3	
525.3348	525.3338	-1.88	-1	8738	9575.2	5.9	6.7	4.3	
526.3377	526.3366	-2.23	-1.2	1245.5	1484	0.8	1	0.6	

Table S6 – NMR data for suberitenone I (**6**) (500 (^{1}H) and 150 (^{13}C) MHz, CD_3OD).

pos	δ_C, type	δ_H	gCOSY	gHMBC	NOESY
1	71.0, CH	4.17 br t (2.7)	2, 6	2, 5, 7	5
2	79.9, CH	4.03, br s	1, 3	1, 4, 6, 8	21
3	48.1, CH	2.64, m	2, 21		
4	212.8, C				
5	46.7, CH_2	2.83, dd (16.2, 5.2)	5', 6	1, 4, 6, 7	1
5'		2.30, dd (16.2, 2.5)	5', 6	1, 4	8
6	45.5, CH	2.68, m	1, 5, 5'		22
7	132.0, C				
8	70.8, CH	3.79, dd (9.4, 6.6)	9, 9'	7, 9, 22	5', 10, 21
9a	26.0, CH_2	1.90, o/l	8, 9'	7, 8, 10, 11, 15	16
9'		1.67, td (12.4, 9.5)	8, 9,10	8, 10, 11	23
10	55.1, CH	1.13, o/l	9'	8, 9, 11, 12, 14, 15, 16, 22, 23, 24	8, 12', 22
11	37.1, C				
12	44.3, CH_2	1.88, o/l	12', 13	10, 11, 13, 14	22, 23
12'		1.41, dd (14.6, 3.7)	12, 13	11, 22	10, 14, 22
13	72.1, CH	5.49, q (3.1)	12, 12', 14		20
14	57.7, CH	1.12, o/l	13	10, 12, 15, 16, 19, 24	12', 16', 20
15	38.0, C				
16	42.6, CH_2	1.74, o/l	16', 17, 17'	14, 18	9
16'		0.85, td (13.1, 3.9)	16, 17, 17'	10, 15, 17, 24	14
17	19.5, CH_2	1.78, o/l	16, 16', 17', 18, 18'		
17'		1.49, m	16, 16', 17, 18, 18'		
18	45.3, CH_2	1.36, m	17, 17', 18'		20, 25
18'		1.23, o/l	17, 17', 18'		
19	35.0, C		17, 17', 18		
20	33.1, CH_3	0.91, s		14, 18, 19, 24	13, 14, 18
21	11.6, CH_3	1.19, d (6.8)		2, 3, 4	2, 8
22	140.9, CH	5.24, s		6, 8, 10, 11, 12, 23	10, 23, 12, 12', 6
23	23.7, CH_3	1.26, s		10, 11, 12, 22	9', 12, 22, 25, 27
24	18.1, CH_3	1.24, s		10, 14, 15, 16	25, 27
25	23.5, CH_3	1.03, s		14, 18, 19, 20	18, 23, 24, 27
26	172.1, C				
27	21.8, CH_3	2.04, s		26	23, 24, 25

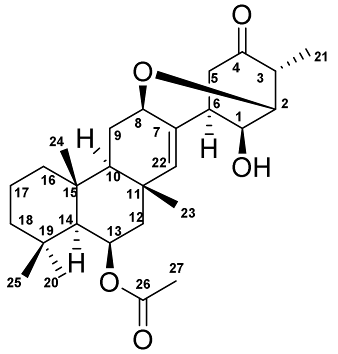

Table S7 – Assigned NMR data for compound **7** (500 (^{1}H) and 150 (^{13}C) MHz, CD_3OD) using correlations observed when acquiring data on **6**.

pos	δ_C, type	δ_H	gHMBC	NOESY
1	64.4, CH	4.38, br t (4.3)		
2	145.4, CH	6.84, dd (5.6, 1.3)	4, 6, 21	
3	137.1, C			
4	202.7, C			
5	37.6, CH_2	2.72, dd (16.3, 13.7)		
5'		2.21, dd (16.2, 3.6)		
6	40.5, CH	3.14, m		
7	135.9, C			
8	70.3, CH	4.30, m	7, 22	10
9a	29.4, CH_2	2.11, dd (12.5, 7.8)	7	
9'		1.59, o/l		
10	54.5, CH	1.37, o/l		8
11	36.7, C			
12	44.8, CH_2	1.89, o/l		
12'		1.48, o/l		
13	72.2, CH	5.50, q (2.3)		
21	15.6, CH_3	1.78, s	2,3,4	
22	140.1, CH_3	5.11, s	6, 8, 10, 11, 12	

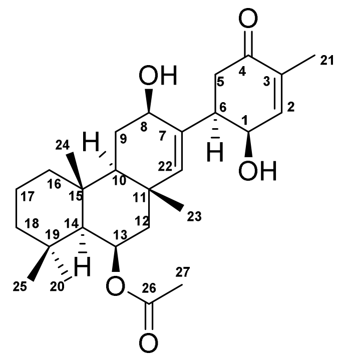

Figure S36 – ^{1}H NMR spectrum (600 MHz, CD_3OD) of **6**

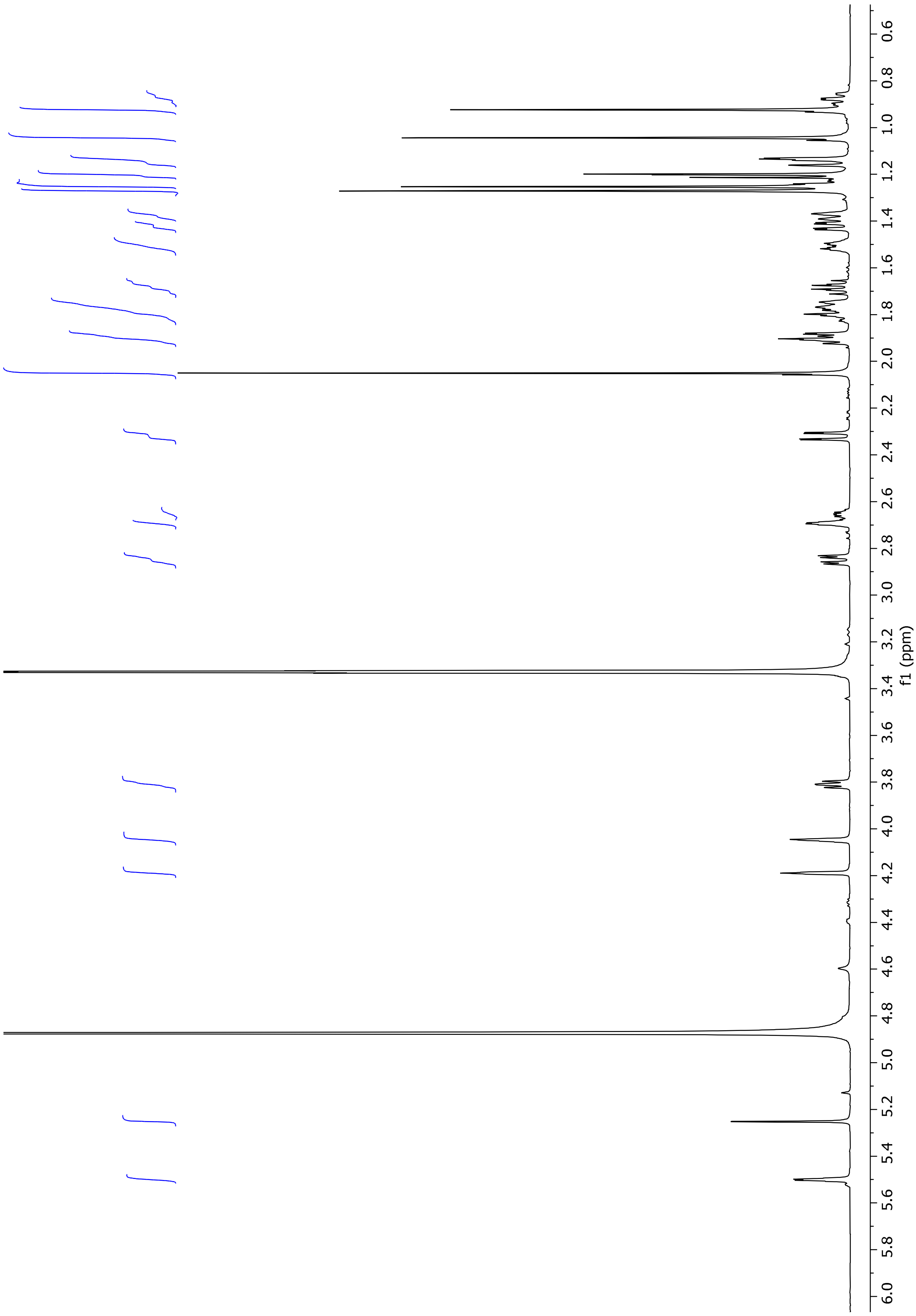

Figure S37 – ^{13}C NMR spectrum (150 MHz, CD_3OD) of **6**

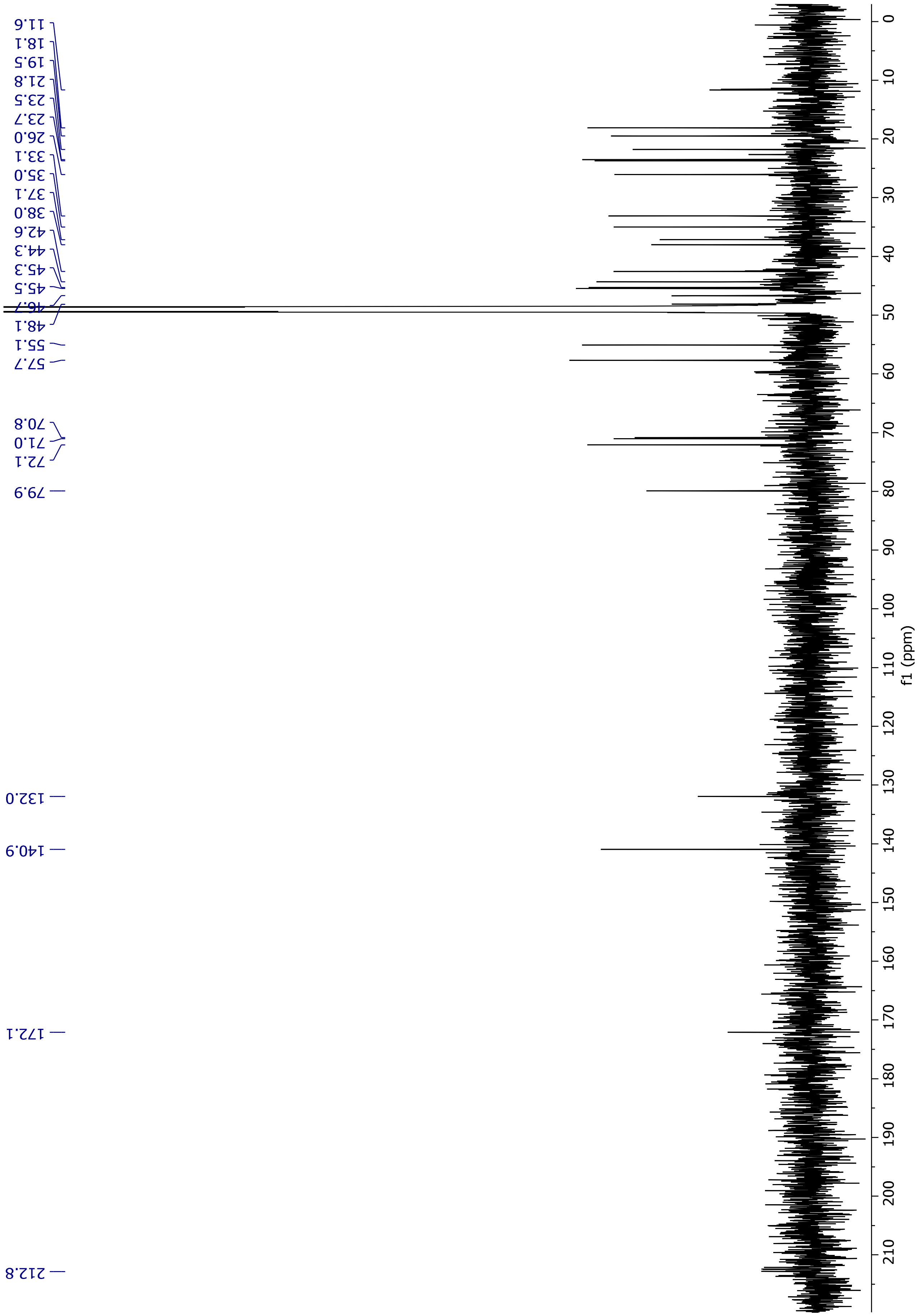

Figure S38 – ^{1}H NMR spectrum (600 MHz, CD_3OD) of **7**

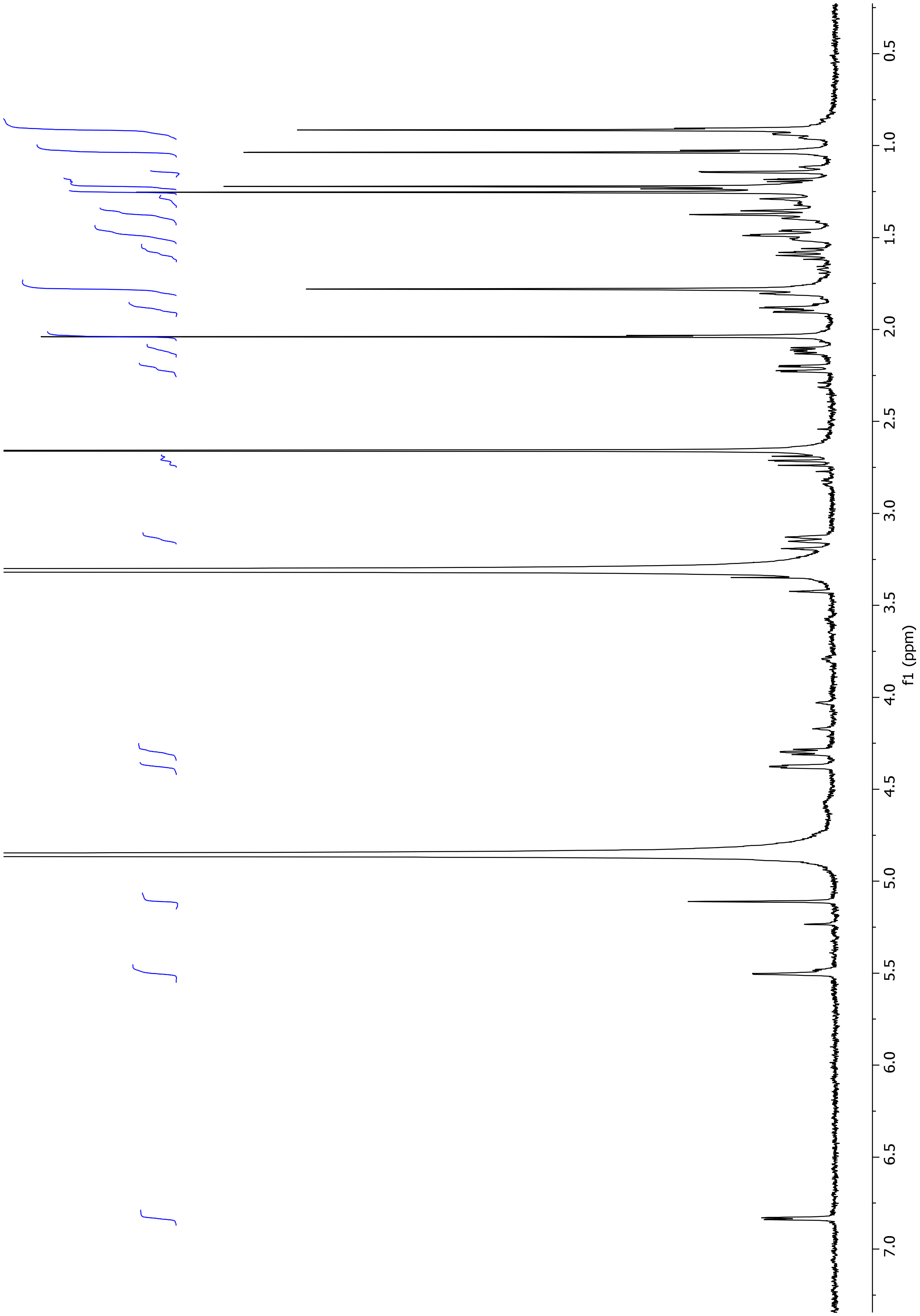

Figure S39 – COSY NMR spectrum (500 MHz, CD_3OD) of **6**

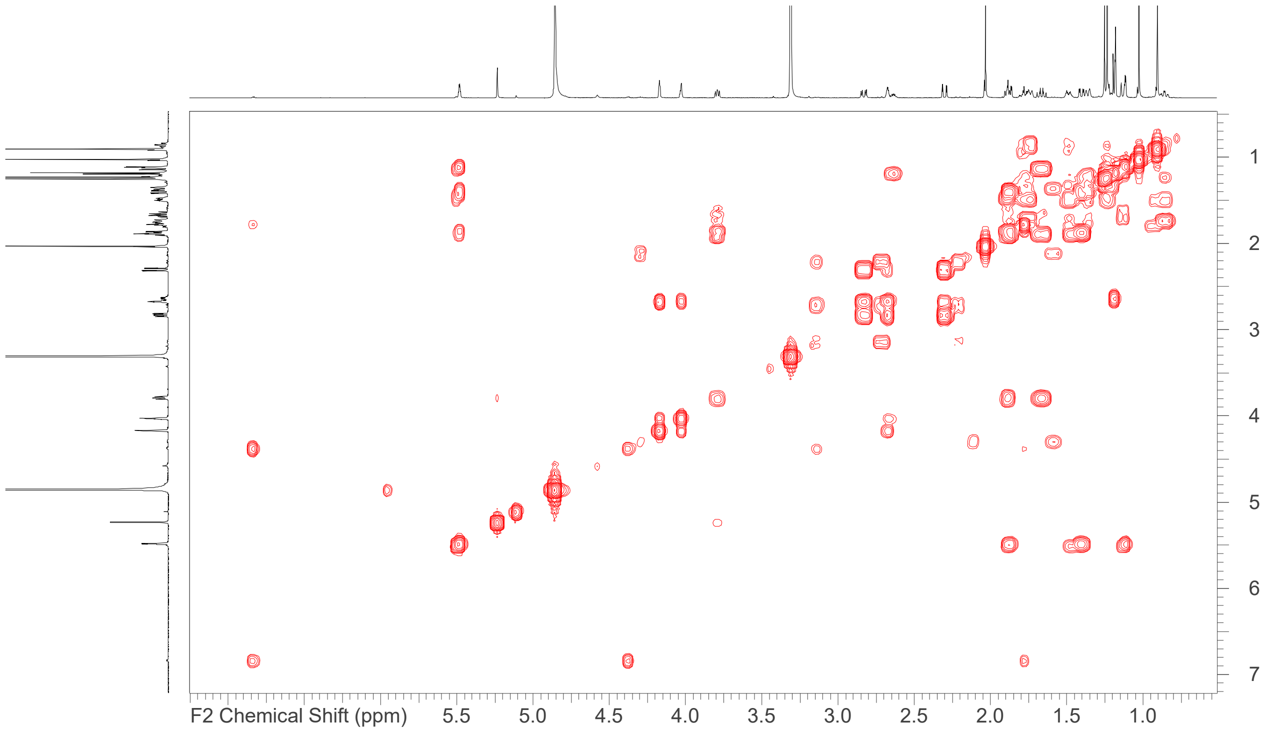

Figure S40 – HSQC NMR spectrum (500 MHz, CD_3OD) of **6** (extra signals due to conversion to **7** during acquisition).

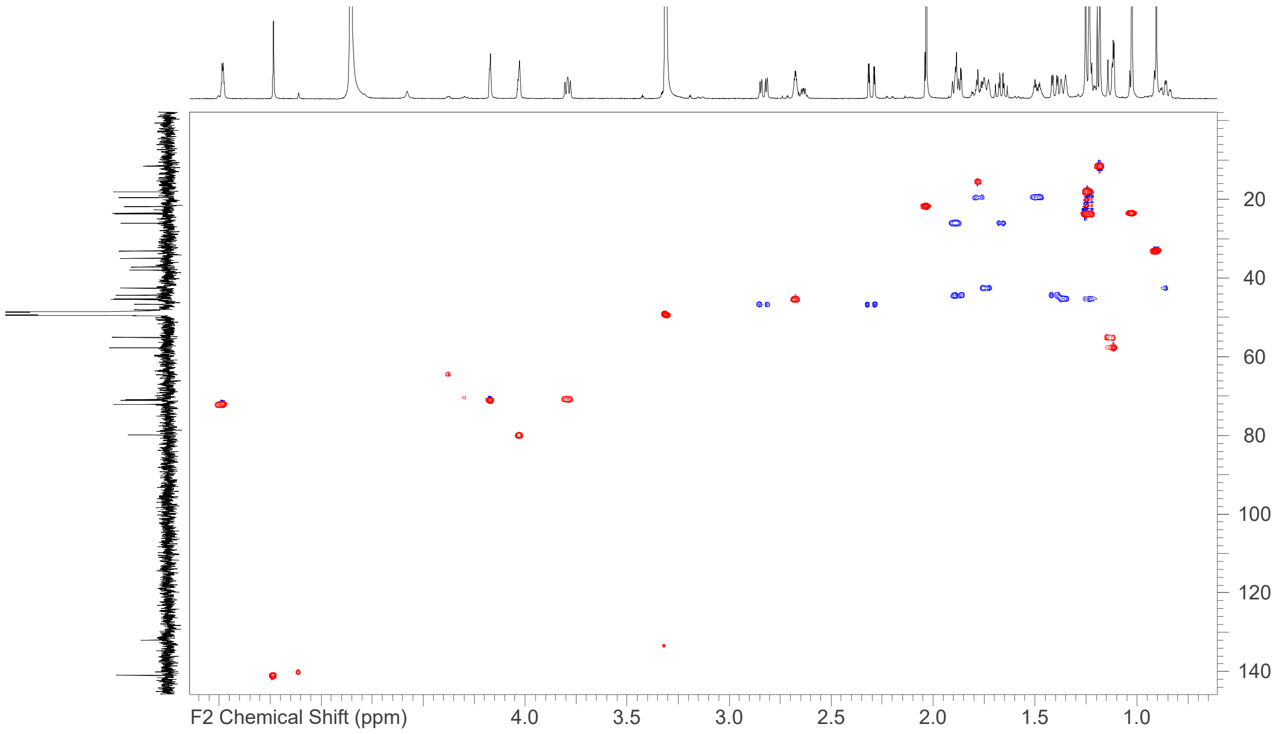

Figure S41 – HMBC NMR spectrum (500 MHz, CD_3OD) of **6** (extra signals due to conversion to **7** during acquisition).

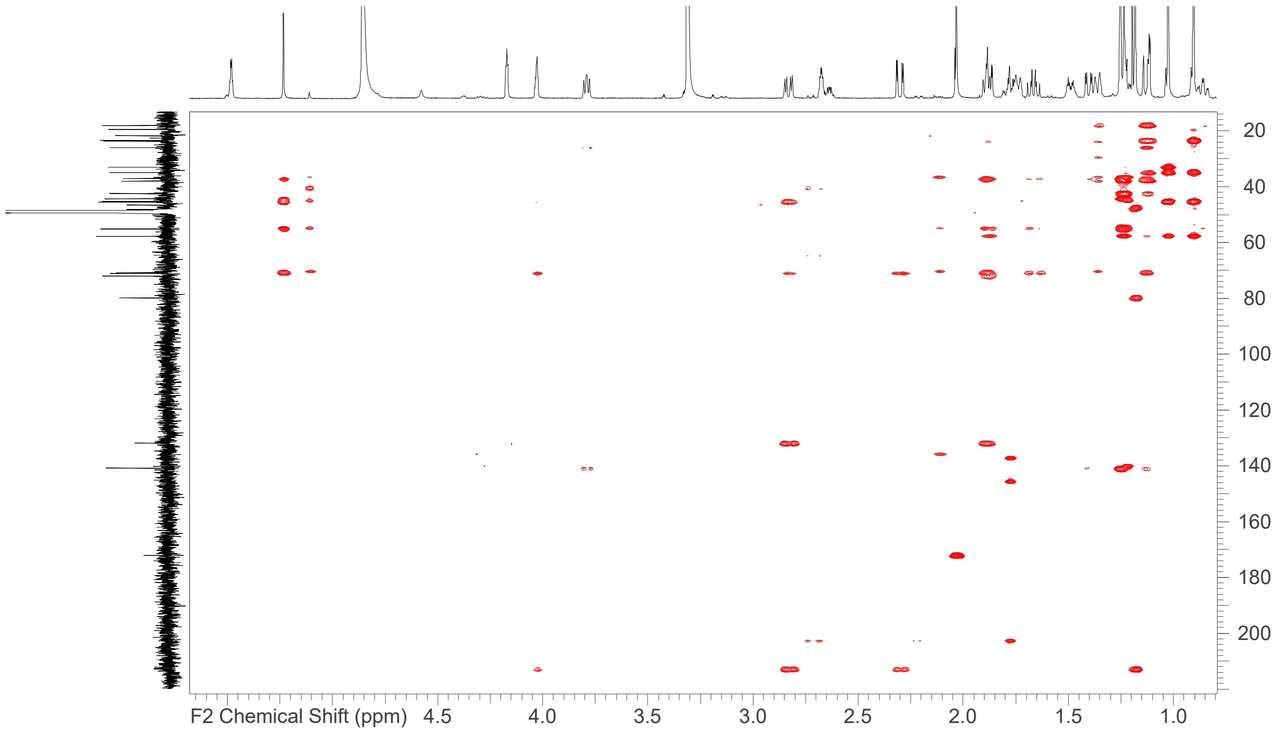

Figure S42 – NOESY NMR spectrum (600 MHz, CD_3OD) of **6** (extra signals due to conversion to **7** during acquisition).

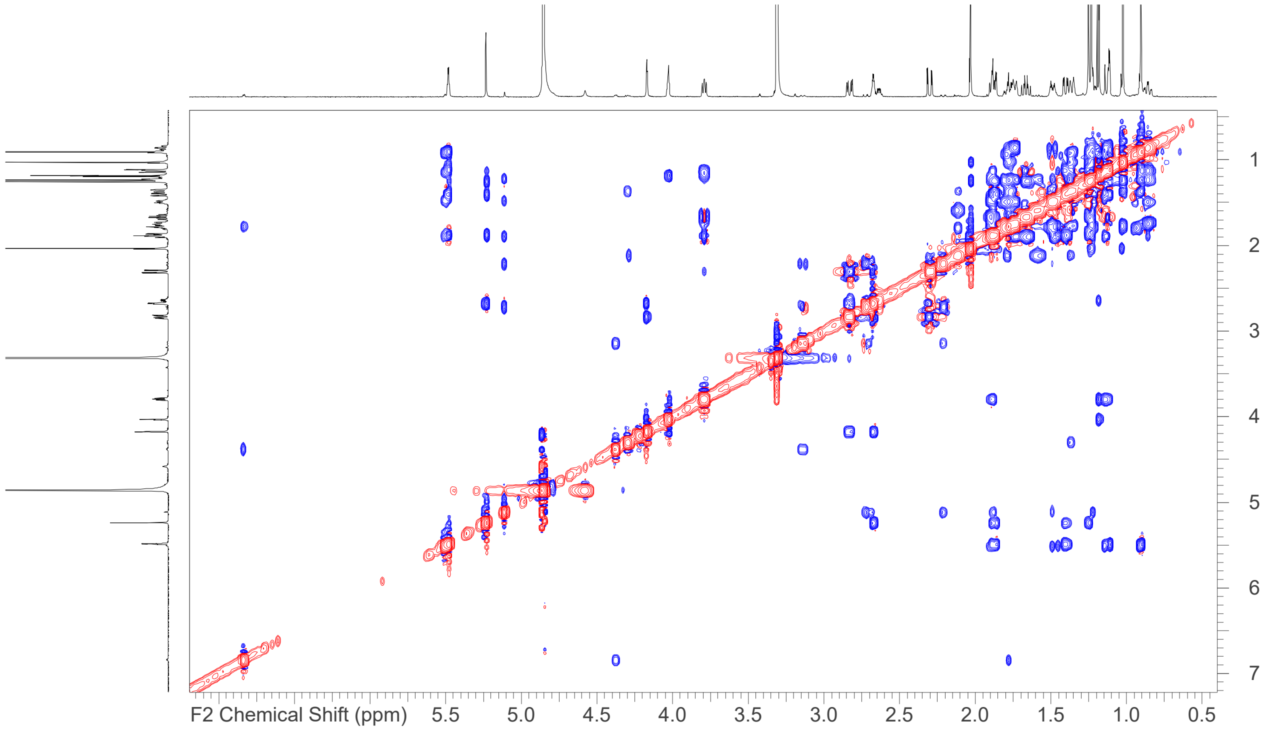

Figure S43 – HRESIMS analysis of **6**

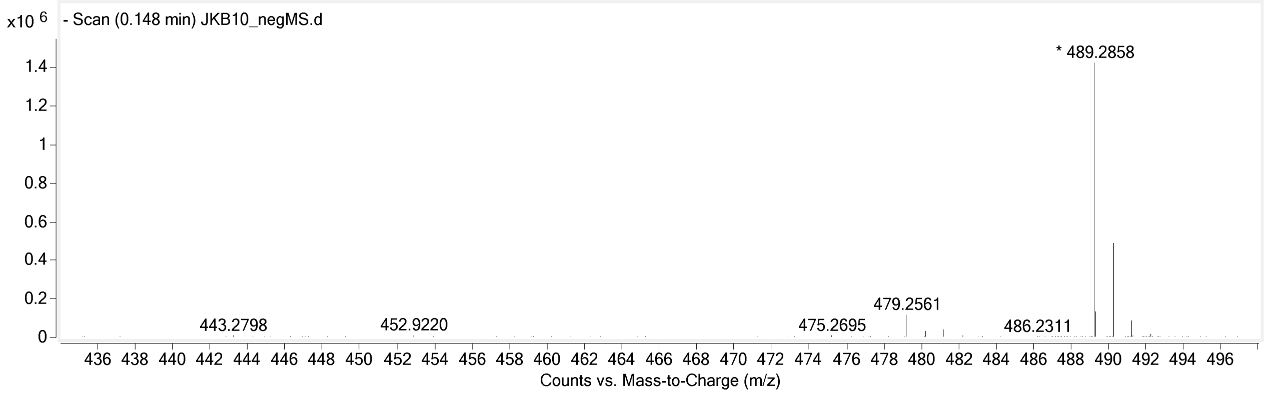

Best	Name	Formula	Score	Mass	Mass (DB)	Mass (MFG)	Diff (ppm)
⊙		C27 H40 O5	99.85	444.28732			0.57

Species	Ion Formula	m/z	Height	Score (MFG)	Score (MS)	Score (mass)	S
(M-H)-		443.2799	883.7		79.42	98.5	
(M+Cl)-		479.2562	6973.1		81.39	97.16	
(M+HCOO)-		489.2855	197169.1		99.85	99.76	

m/z	m/z (Calc)	Diff (ppm)	Diff (mDa)	Height	Height (Calc)	Height %	He
489.2855	489.2858	0.54	0.3	197169.1	196111.7	100	
490.289	490.2892	0.35	0.2	60595.7	60838.3	30.7	
491.2917	491.292	0.63	0.3	11455.3	11936.4	5.8	
492.2972	492.2947	-5.05	-2.5	1392.3	1752.5	0.7	
493.2995	493.2974	-4.26	-2.1	236	209.4	0.1	

Figure S44 – Chromatogram of C18 HPLC separation of **6** and **7** at 210 nm and their UV/Vis spectra.

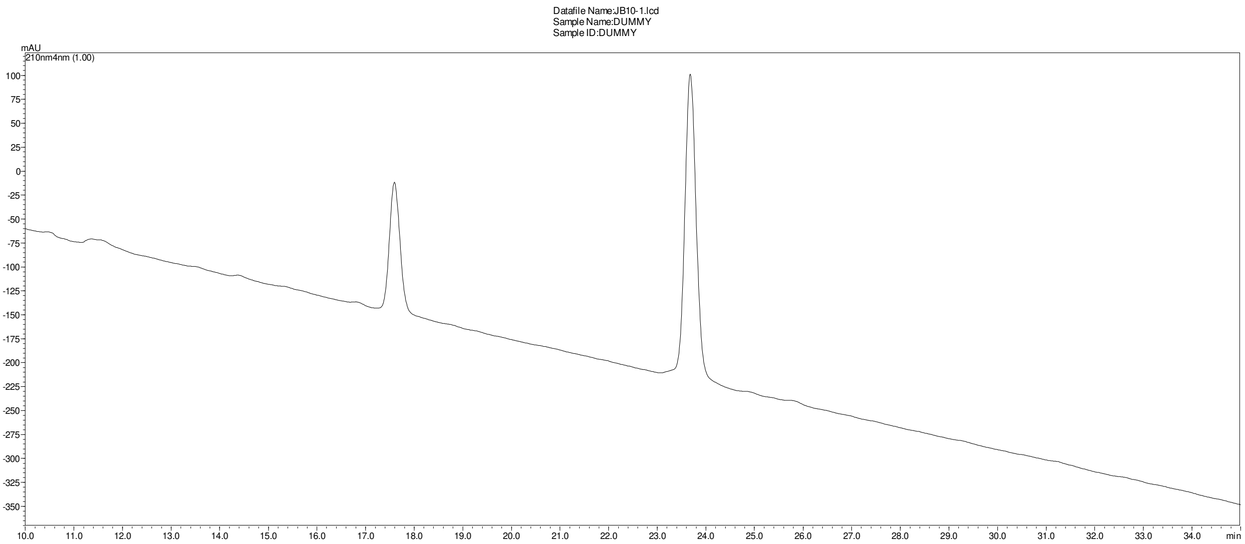

6

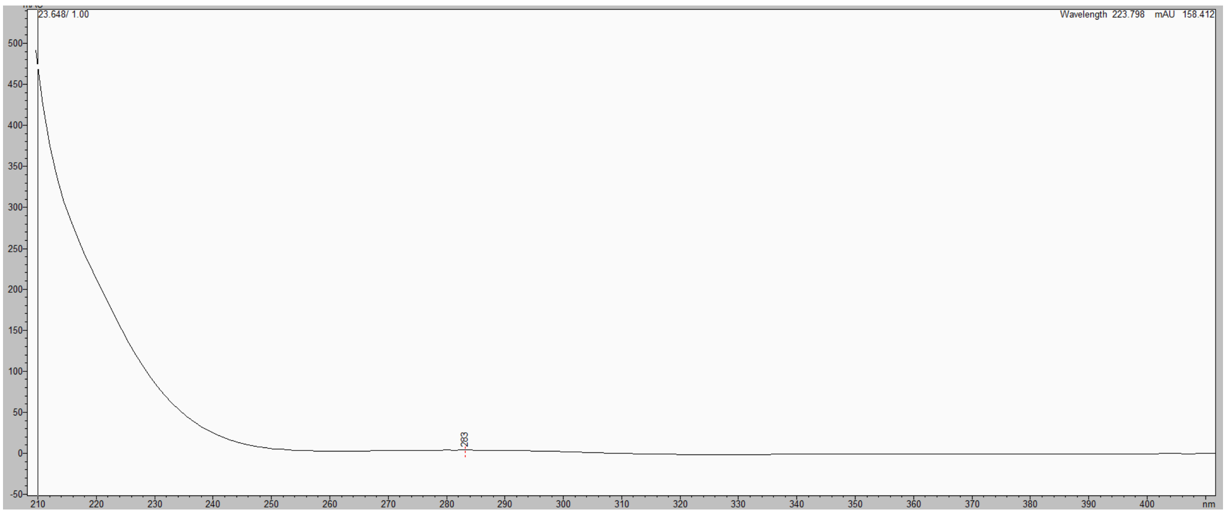

7

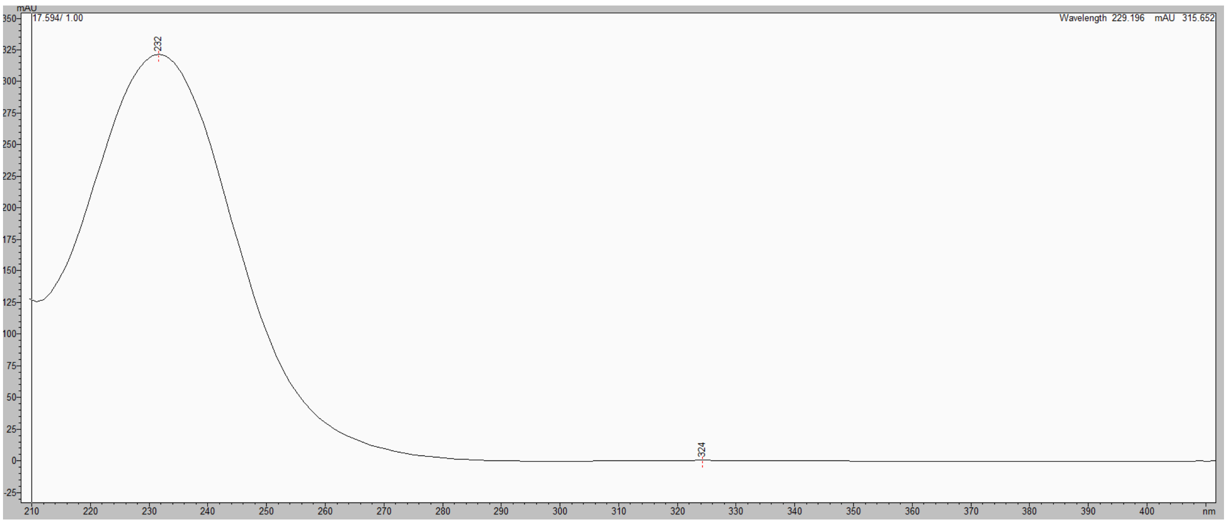

Table S8 – NMR data for secosuberitenone A (**8**) (600 (^{1}H) and 150 (^{13}C) MHz, $CDCl_3$).

pos	δ_C, type	δ_H	gCOSY	gHMBC	NOESY
**1**	63.6, CH	4.31, br t	2, 6, 21	2, 3, 5	2, 6, 22’
**2**	141.7, CH	6.78, br d (4.4)	1, 21	1, 4, 6, 21	1, 8’
**3**	137.6, C				
**4**	200.0 C				
**5**	37.3, CH_2	2.84, m	5’, 6	1, 4, 6	8, 22’
**5’**		2.36, o/l	5, 6	1, 3, 4, 6	8’
**6**	45.5, CH	2.75, m	1, 5, 5’, 22	1, 4	1, 8’
**7**	148.8, C				
**8**	34.2, CH_2	2.35, o/l	8’, 9	7, 9, 10, 22	
**8’**		1.86 o/l	8, 9, 9’	6, 7, 9, 22	2, 6, 12
**9**	22.5, CH_2	1.76, o/l	8, 8’, 9’	7, 8, 10, 11	17’, 22
**9’**		1.52, o/l	8, 8’, 9, 10	7, 8, 10, 11	22
**10**	57.5, CH	1.68, m	9’, 23, 23’	8, 11, 12, 15, 16, 24	14
**11**	144.4, C				
**12**	47.8, CH_2	2.34, o/l	13, 23, 23’	11, 13, 23	8’, 13, 22
**13**	69.5, CH	4.38, br t	12, 14		12, 14, 16’, 20
**14**	57.6, CH	1.08, o/l	13	13, 15, 19, 20, 24, 25	10, 13, 20
**15**	41.2, C				
**16**	42.2, CH_2	1.78, o/l	16’, 17, 17’, 18		16’, 25
**16’**		1.07, o/l	16, 17, 17’		13, 16
**17**	19.7, CH_2	1.64, m	16, 16’, 18		
**17’**		1.50, m	16, 16’, 17, 18’		
**18**	44.0, CH_2	1.39, m	16, 17, 17’, 18’		18’, 20
**18’**		1.19, m	17’, 18	17, 25	18
**19**	34.6, C				
**20**	33.8, CH_3	1.01, s	25	14, 15, 17, 19, 25	13, 14, 16, 18
**21**	15.8, CH_3	1.84, s	1, 2	1, 2, 3, 4	
**22**	112.4, CH_2	5.16, s	22’	1, 6, 7, 8	9, 9’, 22’
**22’**		4.92, s	6, 22	1, 6, 7, 8	1, 5, 22, 23, 23’
**23**	110.4, CH_2	5.03, s	10, 12, 23’	10, 11, 12	22’, 23’
**23’**		4.77, s	10, 12, 23	10, 11, 12	22’, 23
**24**	17.3, CH_3	1.00, s		10, 14, 15, 16	25
**25**	23.8, CH_3	1.22, s	20	14, 18, 19, 20	24

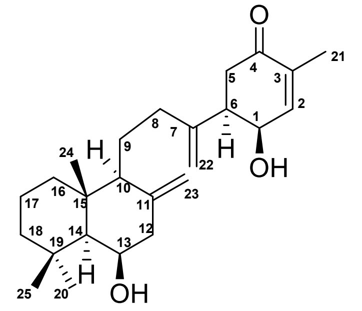

Figure S45 – ^{1}H NMR spectrum (600 MHz, $CDCl_3$) of **8**

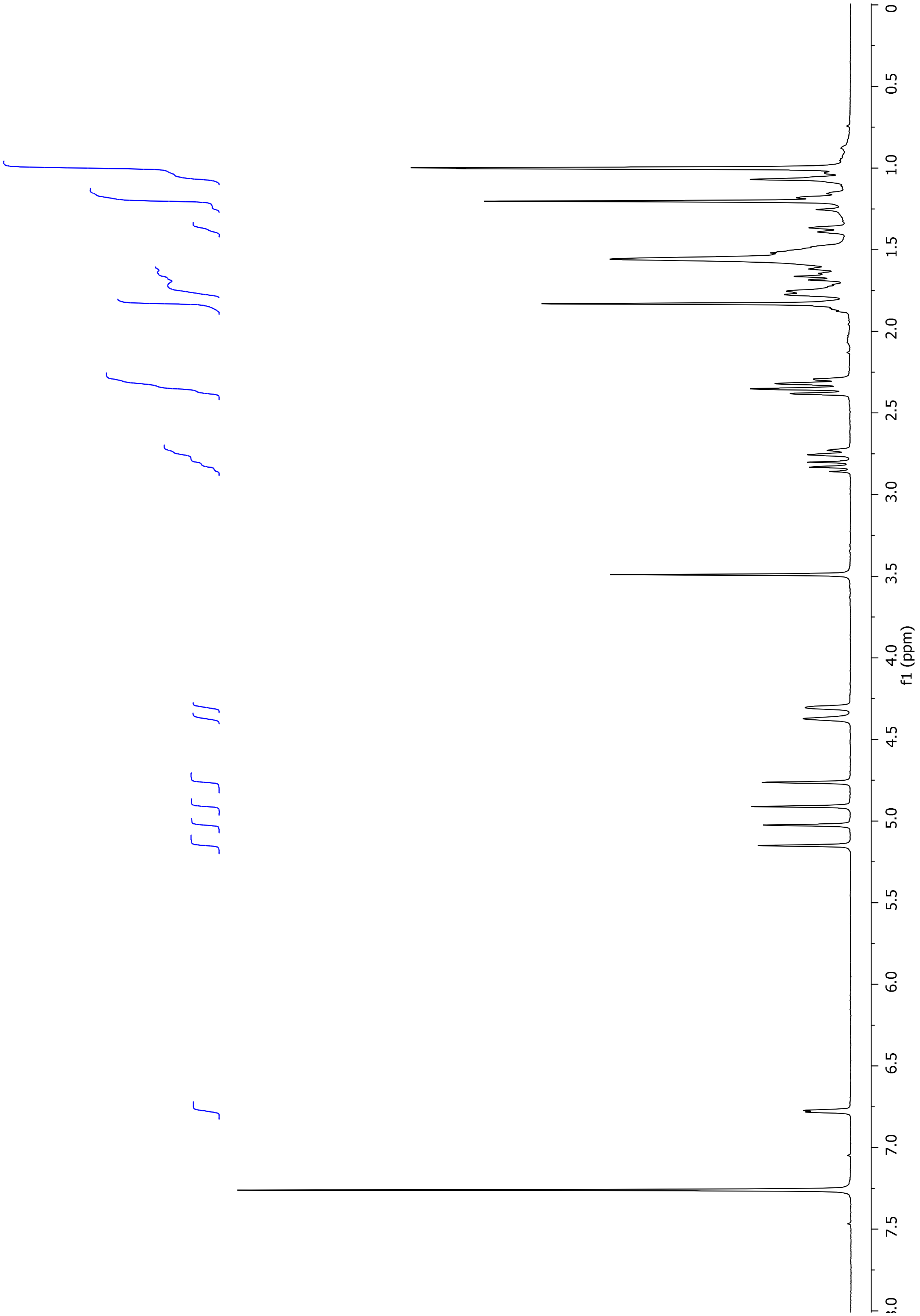

Figure S46 – ^{13}C NMR spectrum (125 MHz, $CDCl_3$) of **8**

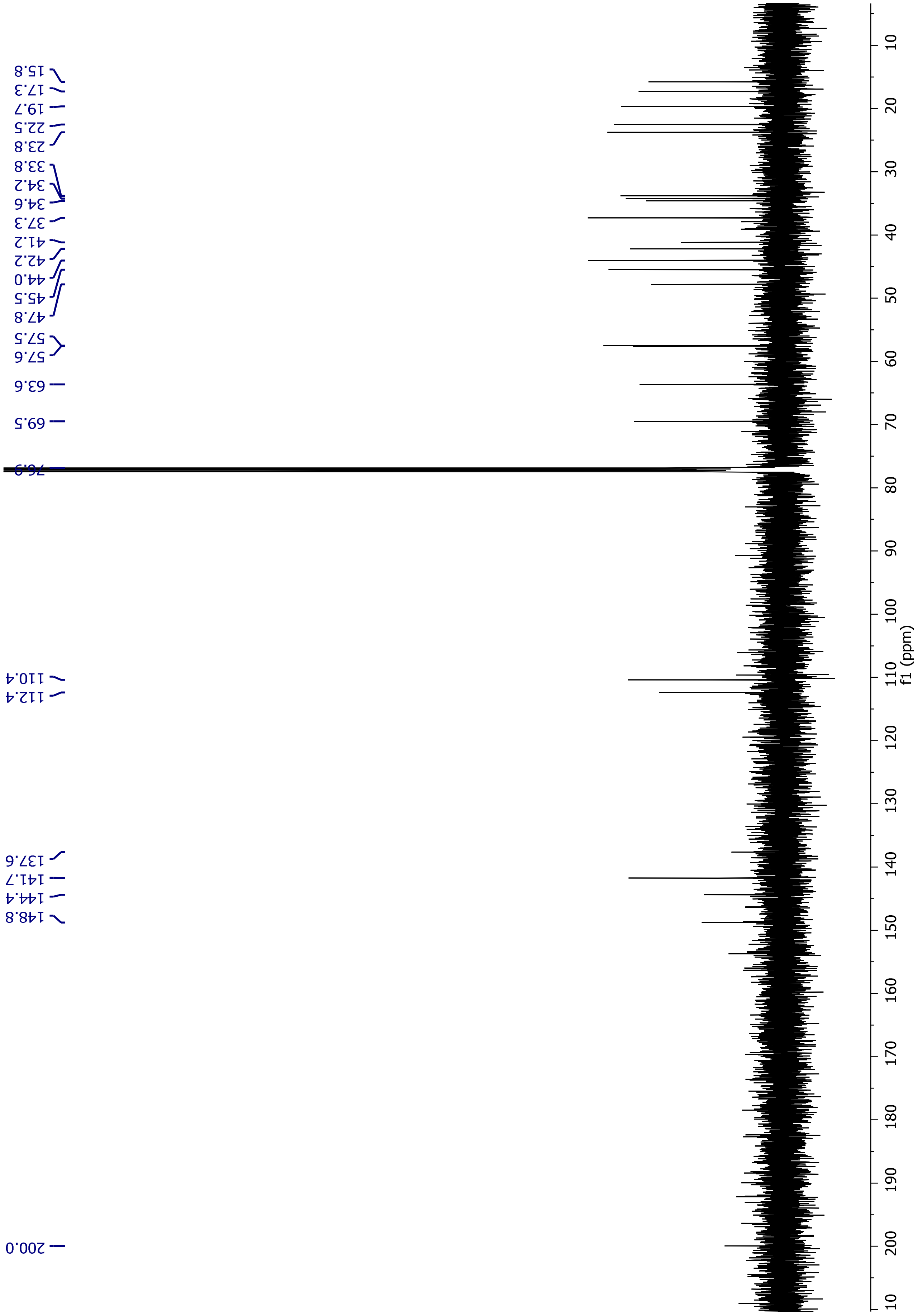

Figure S47 – COSY NMR spectrum (600 MHz, $CDCl_3$) of **8**

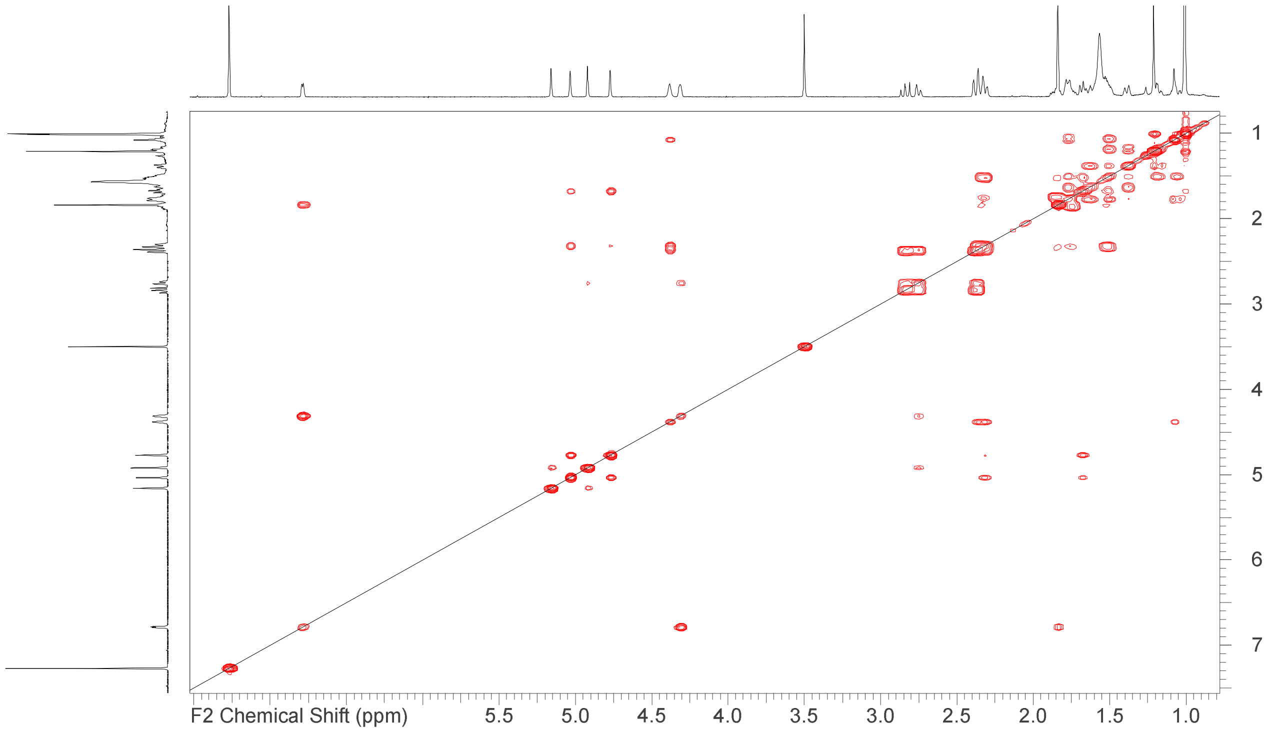

Figure S48 – HSQC NMR spectrum (600 MHz, $CDCl_3$) of **8**

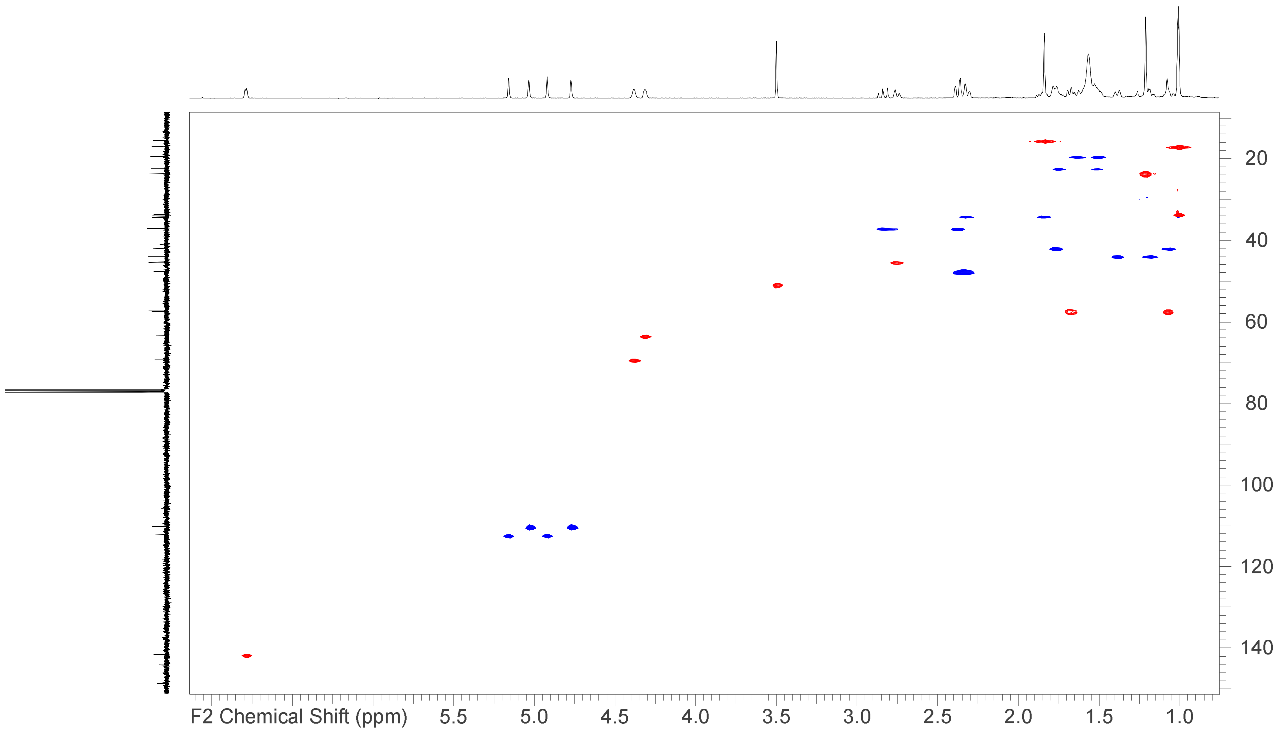

Figure S49 – HMBC NMR spectrum (600 MHz, $CDCl_3$) of **8**

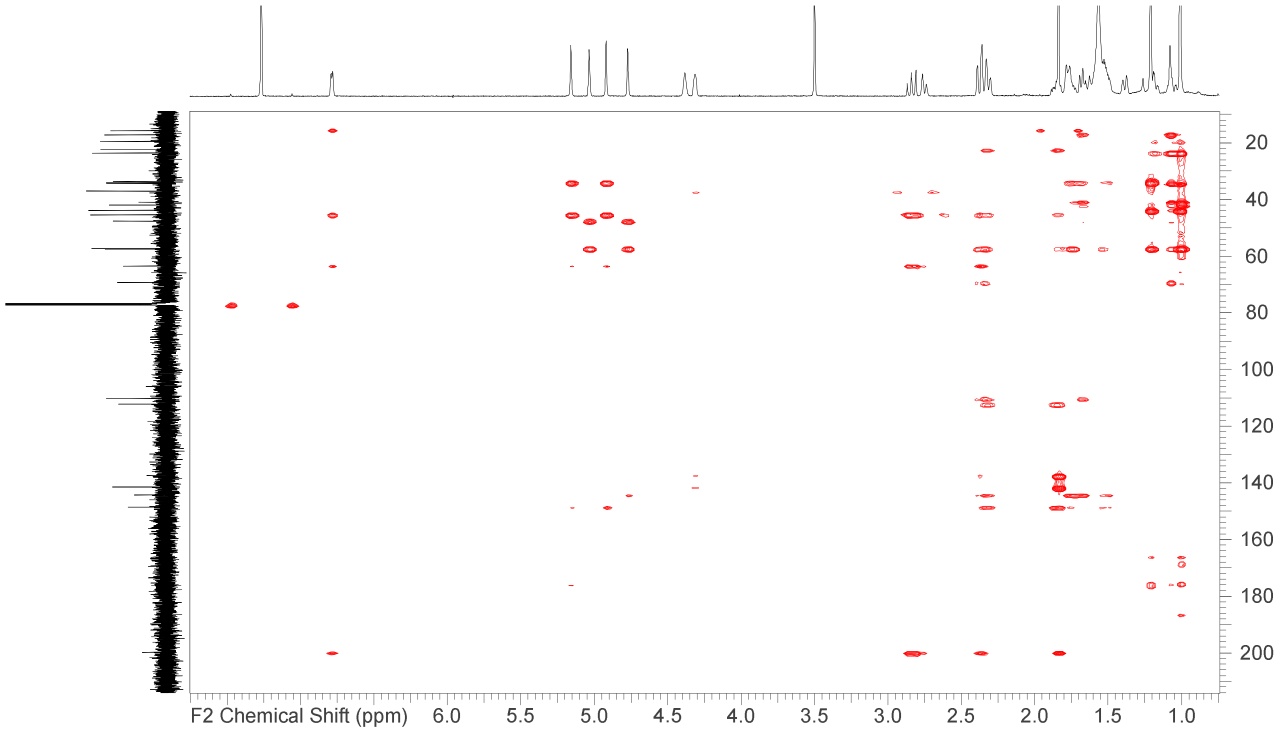

Figure S50 – NOESY NMR spectrum (600 MHz, $CDCl_3$) of **8**

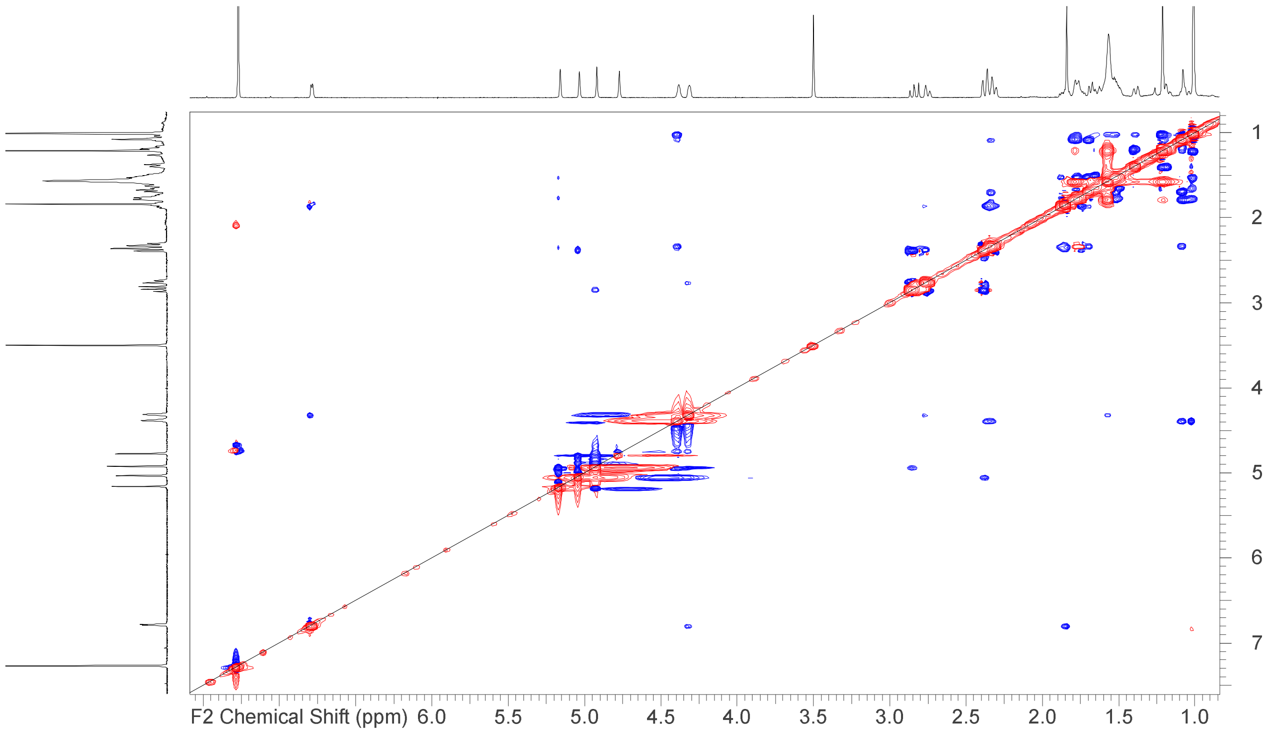

Figure S51 – HRESIMS analysis of **6**

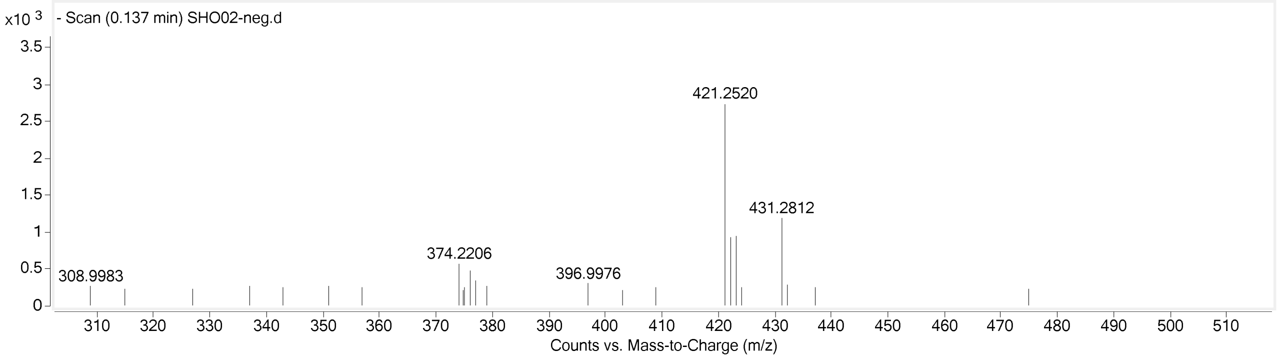

Best	Name	Formula	Score	Mass	Mass (DB)	Mass (MFG)	Diff (ppm)
⦿		C25 H38 O3	78.24	386.28256			-1.19

Species	Ion Formula	m/z	Height	Score (MFG)	Score (MS)	Score (mass)	S
(M+Cl)-		421.2531	1468.7		71.42	99.83	
(M+HCOO)-		431.2814	1085.7		78.24	95.21	

m/z	m/z (Calc)	Diff (ppm)	Diff (mDa)	Height	Height (Calc)	Height %	He
431.2814	431.2803	-2.51	-1.1	1085.7	1052.6	100	
432.2843	432.2837	-1.29	-0.6	269.7	302.7	24.8	

Table S9 – NMR data for norsuberitenone A (**8**) (500 (^{1}H) and 125 (^{13}C) MHz, CD_3OD).

pos	δ_C, type	δ_H	gCOSY	gHMBC	NOESY
**1**	61.0, CH_2	2.21, d (13.1)	1', 16	2, 5, 6, 7, 16	5, 7'
**1'**		1.90, dd (13.1, 2.4)	1, 3'	2, 3, 5, 6, 7, 16	16
**2**	215.1, C				
**3**	42.4, CH_2	2.37, m	3', 4	2, 4	5
**3'**		2.33, m	1', 3, 4'	2, 5	
**4**	23.3, CH_2	2.05, m	3, 4', 5	2, 3, 6	
**4'**		1.78, o/l	3', 4, 5	3, 5, 6	
**5**	58.1, CH	1.56, dd (12.7, 3.0)	4, 4'	1, 4, 6, 10, 16, 17	1, 3, 9
**6**	39.5, C				
**7**	51.2, CH_2	1.76, o/l	7', 8	6, 8, 9, 16	
**7'**		1.52, dd (14.1, 3.3)	7, 8, 16	1, 6, 16	1
**8**	68.6, CH	4.44, q (2.5)	7, 7', 9		15
**9**	58.7, CH	1.02, br d (2.1)	8	8, 10, 13, 14, 15, 17	5
**10**	38.6, C				
**11**	43.6, CH_2	1.75, o/l	11', 12, 12'	9	
**11'**		1.03, o/l	11, 12, 12'		
**12**	19.8, CH_2	1.78, o/l	11, 11', 12', 13, 13'		17, 18
**12'**		1.45, m	11, 11', 12, 13, 13'		
**13**	45.4, CH_2	1.35, m	12, 12', 13'		15
**13'**		1.24, m	12, 12', 13		
**14**	35.2, C				
**15**	33.8, CH_3	0.98, s		9, 13, 14, 18	8, 13, 18
**16**	22.2, CH_3	1.16, s	1, 7'	1, 5, 6, 7	1'
**17**	17.7, CH_3	1.25, s		5, 9, 10, 11	12
**18**	24.1, CH_3	1.23, s		9, 13, 14, 15	12, 15

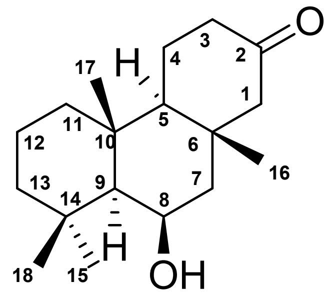

Figure S52 – ^{1}H NMR spectrum (500 MHz, CD_3OD) of **8**

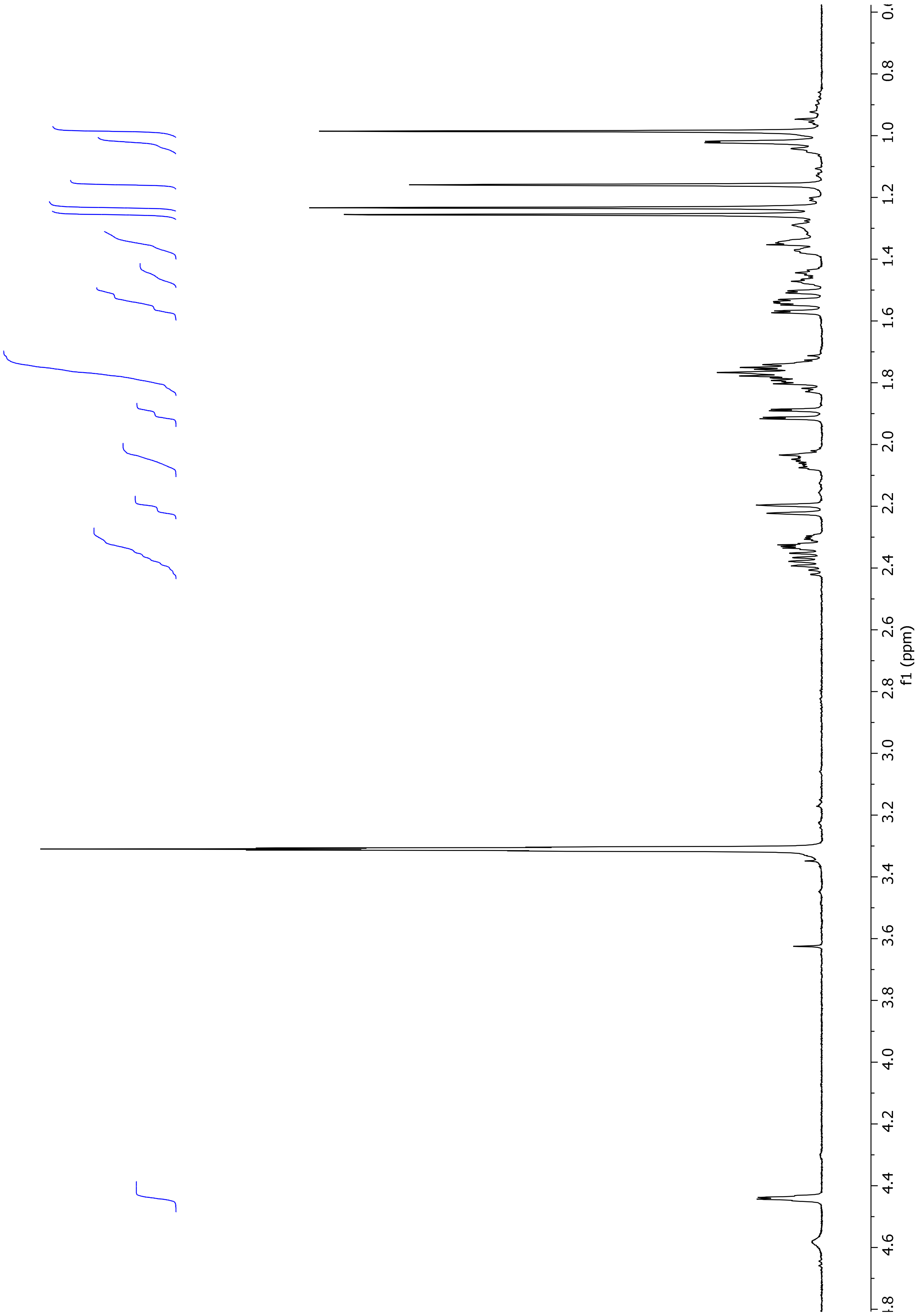

Figure S53 – ^{13}C NMR spectrum (150 MHz, CD_3OD) of **9** (formic acid impurity δ_C 170.3)

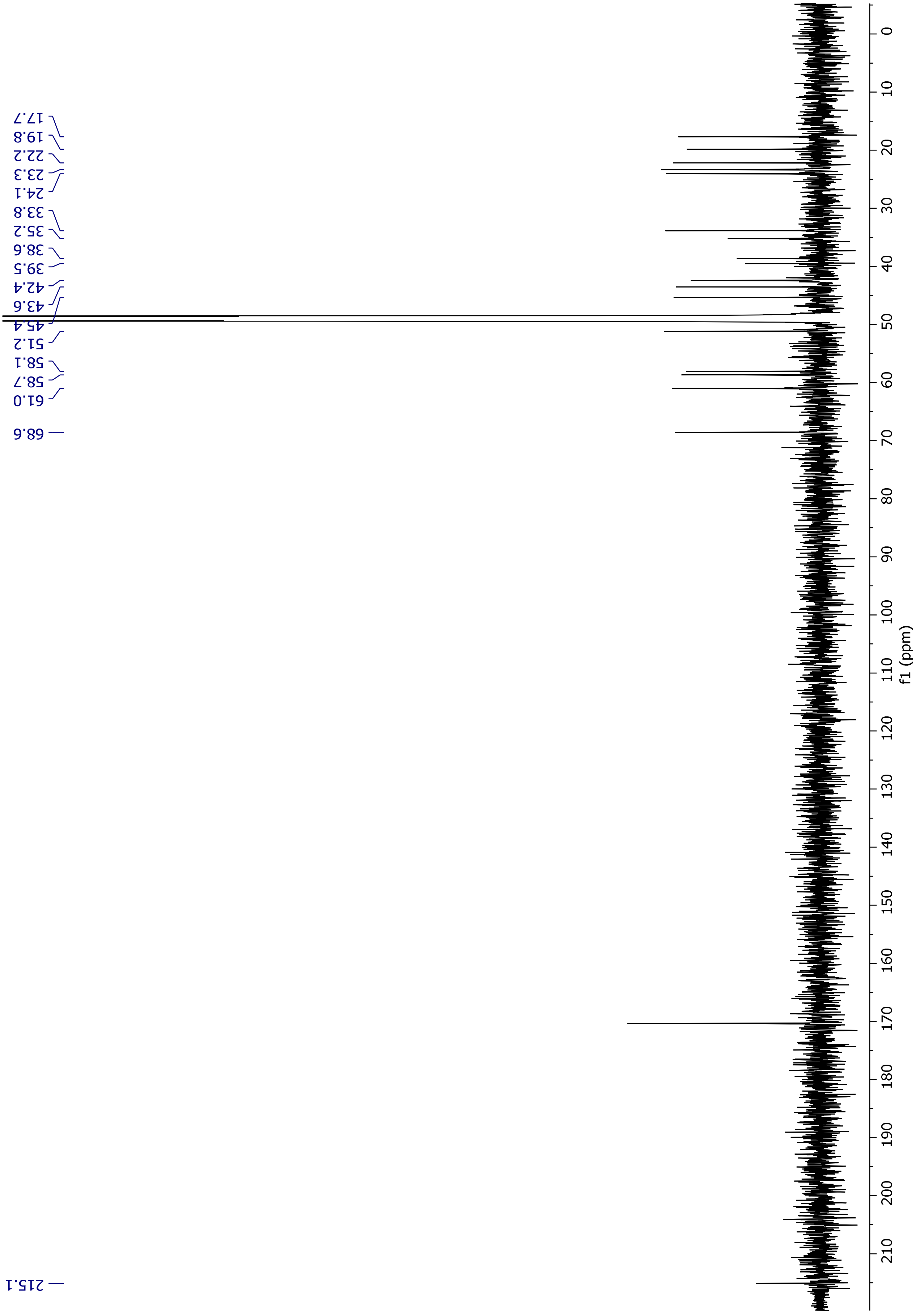

Figure S54 – COSY NMR spectrum (500 MHz, CD_3OD) of **9**

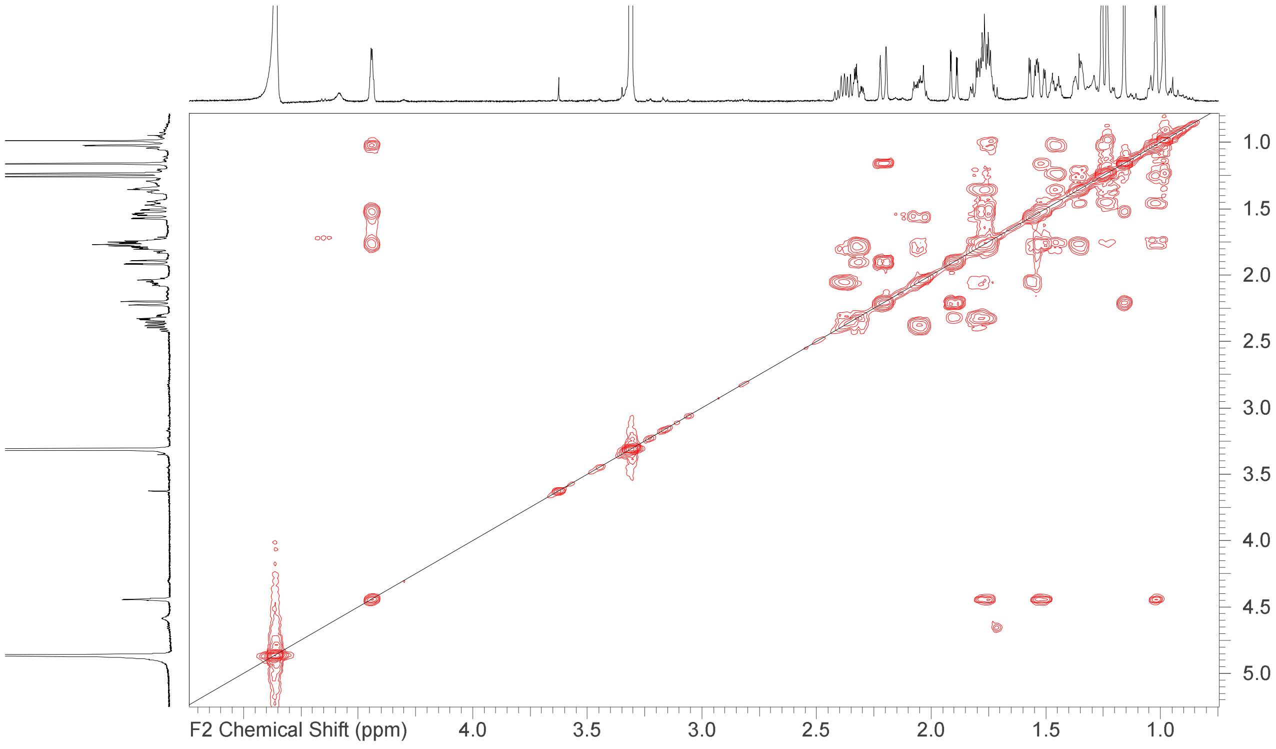

Figure S55 – HSQC NMR spectrum (500 MHz, CD_3OD) of **9**

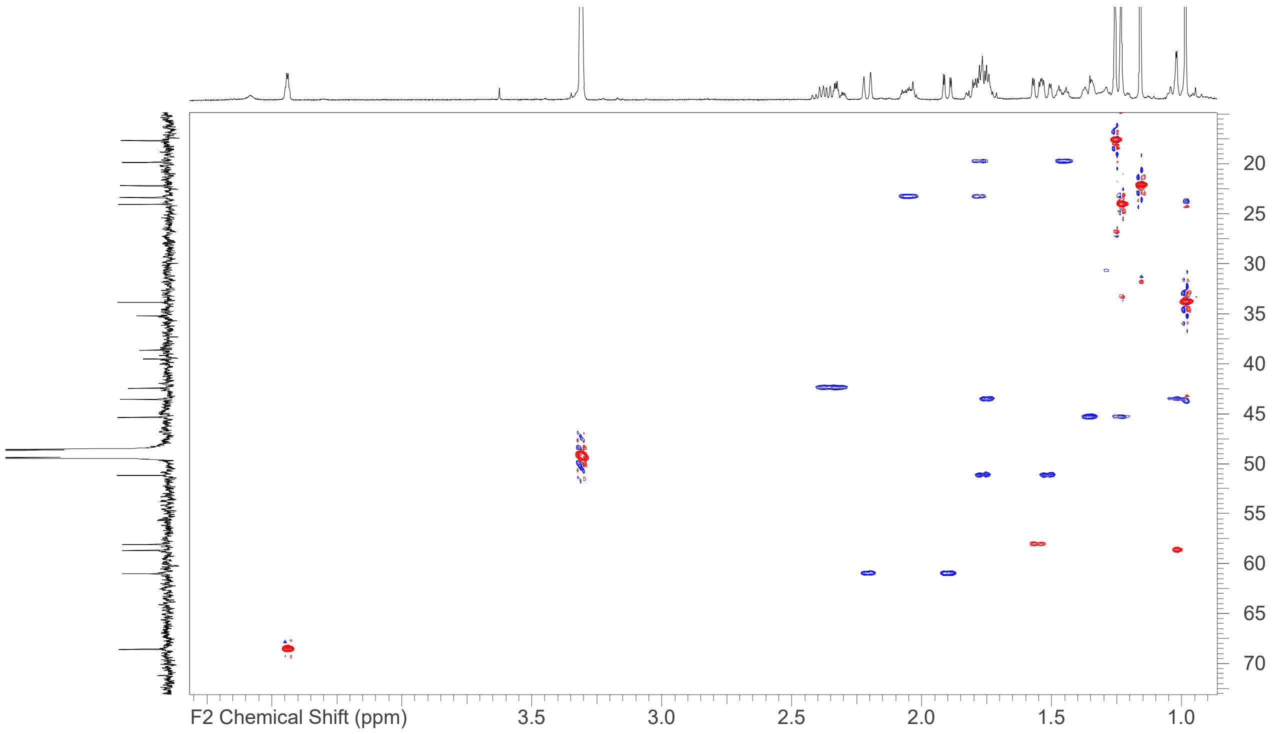

Figure S56 – HMBC NMR spectrum (500 MHz, CD_3OD) of **9**

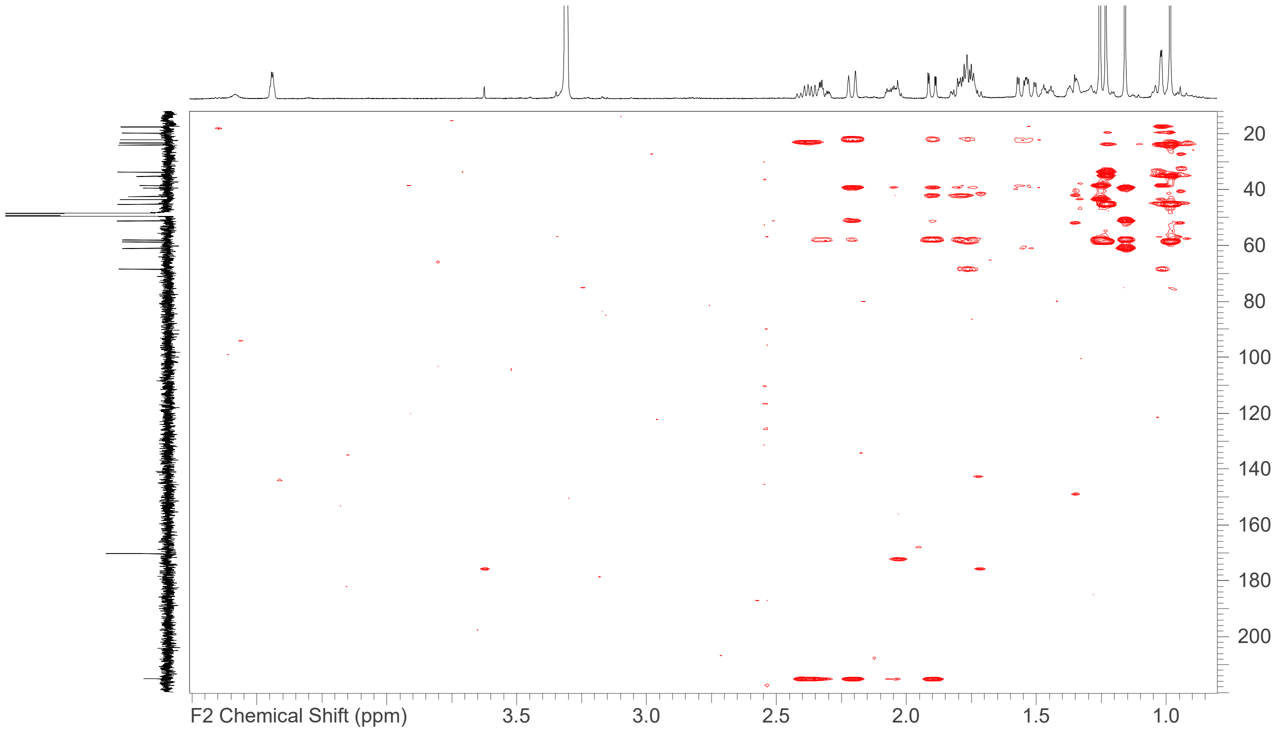

Figure S57 – NOESY NMR spectrum (600 MHz, CD_3OD) of **9**

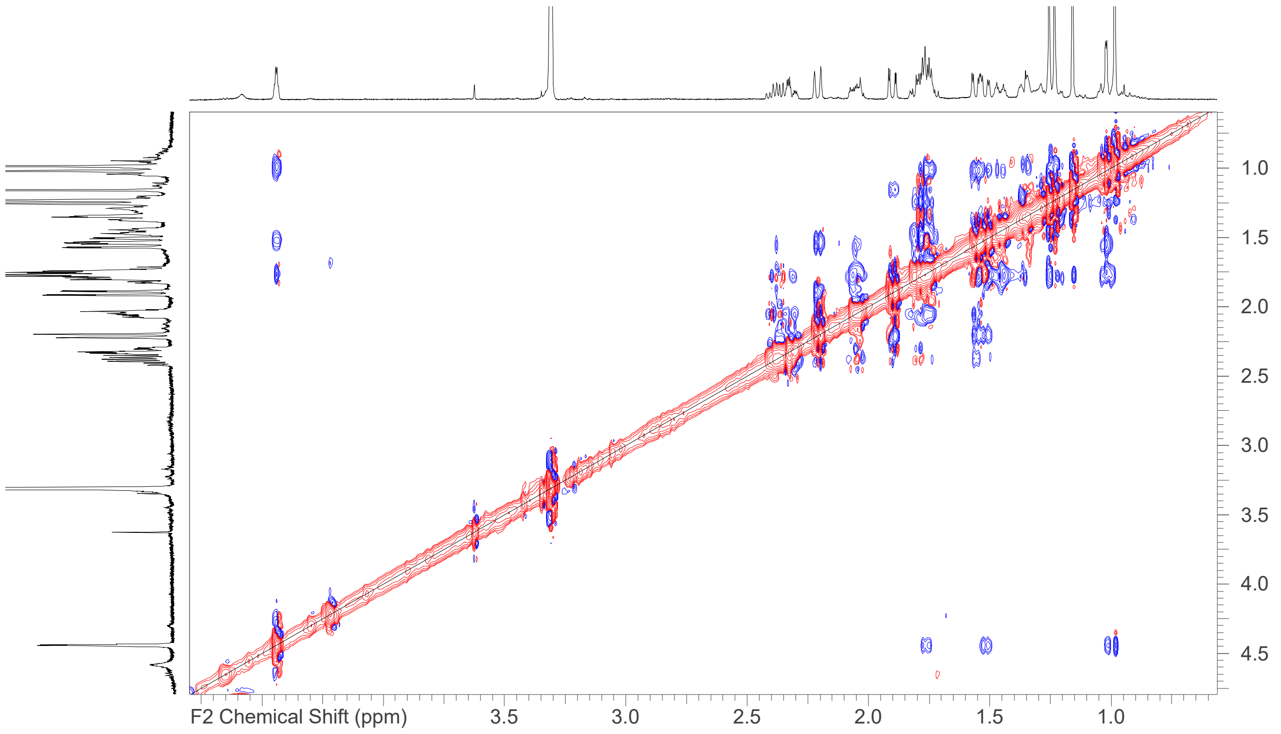

Figure S58 – HRESIMS analysis of **9**

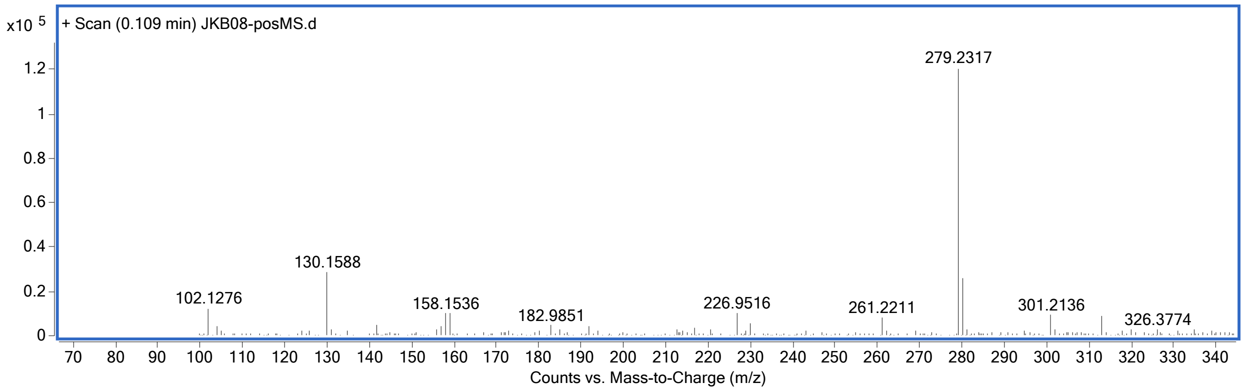

		C18 H30 O2	99.55	278.22476			-0.64	0.64	-0.18	0

Species	Io	m/z	Height	Score (MFG)	Score (MS)	Score (mass)	Score (iso. abund)	Score (iso. spacing)
(M+H)+		279.232	78426.3		99.55	99.75	99.54	99.18

m/z	m/z (Calc)	Diff (ppm)	Diff (mDa)	Height	Height (Calc)	Height %	Height % (Calc)	Height Sum %	He
279.232	279.2319	-0.38	-0.1	77650.2	78426.3	100	100	80.9	
280.23...	280.2353	-1.01	-0.3	15884.3	15607.7	20.5	19.9	16.5	
281.24	281.2382	-6.31	-1.8	2179.6	1792.8	2.8	2.3	2.3	
282.24...	282.241	-6.41	-1.8	263.8	151.2	0.3	0.2	0.3	

Species	Io	m/z	Height	Score (MFG)	Score (MS)	Score (mass)	Score (iso. abund)	Score (iso. spacing)
(M+Na)+		301.214	2937.9		96.05	99.72	89.6	96.46

Table S10 – Crystal data and structure refinement for neosuberitenone A (**1**)

Empirical formula	$C_{27}H_{40}O_4$
Formula weight	428.59
Temperature/K	100.00
Crystal system	monoclinic
Space group	C2
a/Å	24.7343(6)
b/Å	6.27570(10)
c/Å	18.2166(4)
α/°	90
β/°	125.2637(7)
γ/°	90
Volume/Å^3	2308.80(9)
Z	4
ρ_{calc}g/cm^3	1.233
μ/mm^{-1}	0.636
F(000)	936.0
Crystal size/mm^3	0.58 × 0.21 × 0.17
Radiation	CuKα (λ = 1.54178)
2Θ range for data collection/°	5.942 to 159.866
Index ranges	$-31 \leq h \leq 31$, $-7 \leq k \leq 7$, $-22 \leq l \leq 22$
Reflections collected	21421
Independent reflections	4852 [R_{int} = 0.0409, R_{sigma} = 0.0334]
Data/restraints/parameters	4852/1/290
Goodness-of-fit on F^2	1.058
Final R indexes [I>=2σ (I)]	R_1 = 0.0330, wR_2 = 0.0879
Final R indexes [all data]	R_1 = 0.0332, wR_2 = 0.0881
Largest diff. peak/hole / e Å^{-3}	0.24/-0.20
Flack parameter	0.06(6)

Table S11 – Crystal data and structure refinement for suberitenone E (**2**)

Empirical formula	$C_{27}H_{40}O_5$
Formula weight	444.59
Temperature/K	298.00
Crystal system	monoclinic
Space group	$P2_1$
a/Å	6.7821(2)
b/Å	8.9780(3)
c/Å	20.5457(6)
α/°	90
β/°	95.5970(10)
γ/°	90
Volume/Å^3	1245.06(7)
Z	2
ρ_{calc}g/cm^3	1.186
μ/mm^{-1}	0.638
F(000)	484.0
Crystal size/mm^3	0.4 × 0.16 × 0.07
Radiation	CuKα (λ = 1.54178)
2Θ range for data collection/°	8.648 to 158.82
Index ranges	$-8 \leq h \leq 8$, $-9 \leq k \leq 10$, $-26 \leq l \leq 25$
Reflections collected	28530
Independent reflections	5185 [R_{int} = 0.0572, R_{sigma} = 0.0426]
Data/restraints/parameters	5185/2/299
Goodness-of-fit on F^2	1.079
Final R indexes [I>=2σ (I)]	R_1 = 0.0465, wR_2 = 0.1281
Final R indexes [all data]	R_1 = 0.0504, wR_2 = 0.1325
Largest diff. peak/hole / e Å^{-3}	0.20/-0.20
Flack parameter	0.04(11)

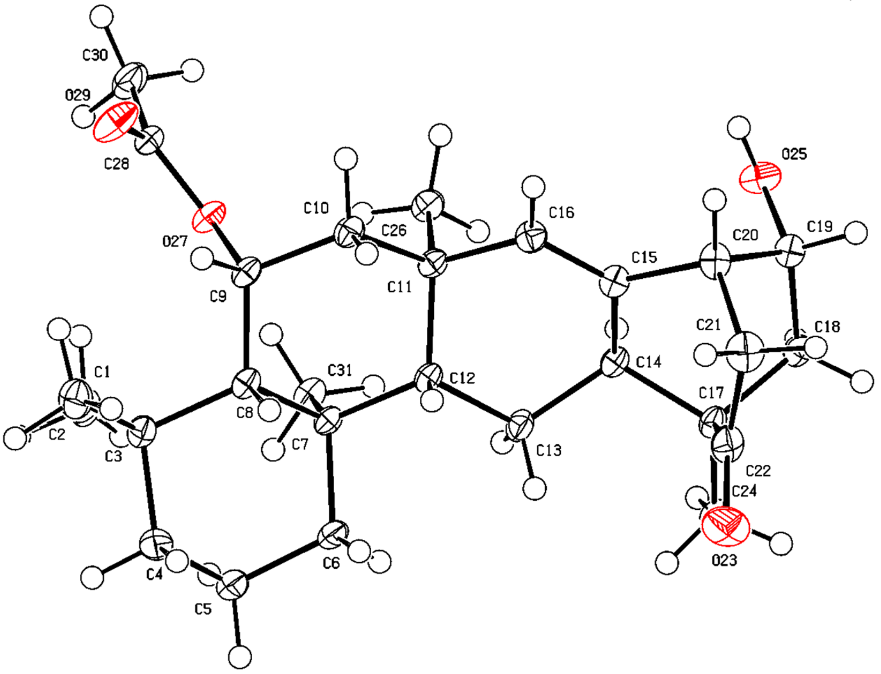

Figure.S59. Ellipsoid plot of neosuberitenone A (**1**). Anisotropic displacement parameters were drawn at 50% probability level.

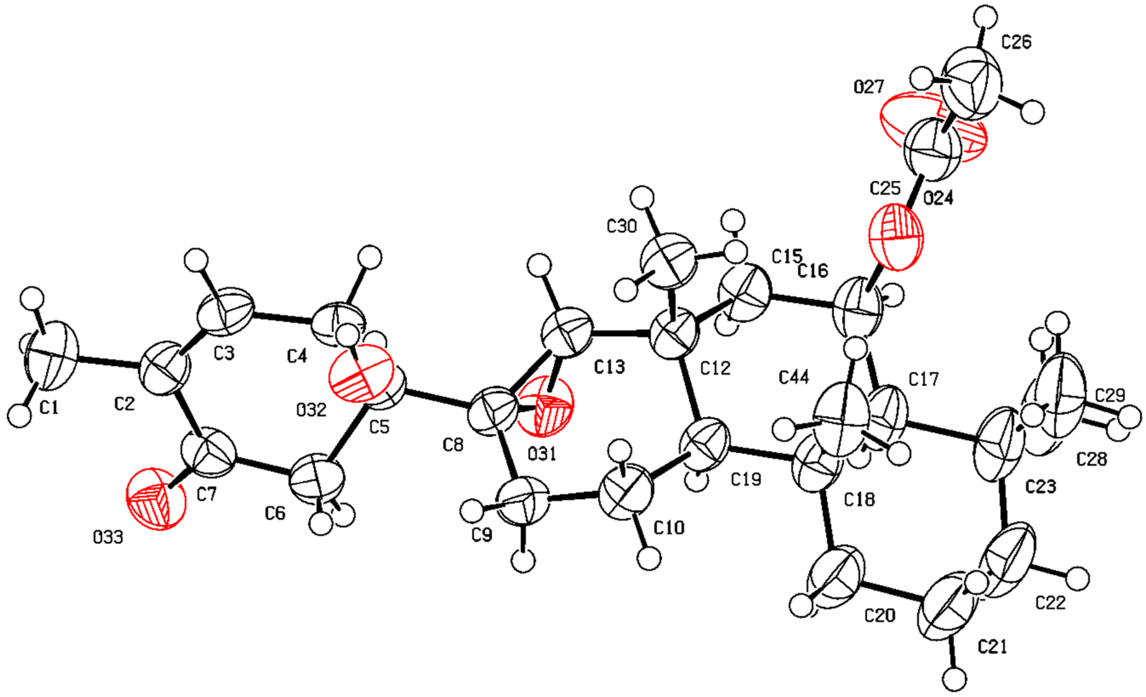

Figure.S60. Ellipsoid plot of suberitenone E (**2**). Anisotropic displacement parameters were drawn at 50% probability level.

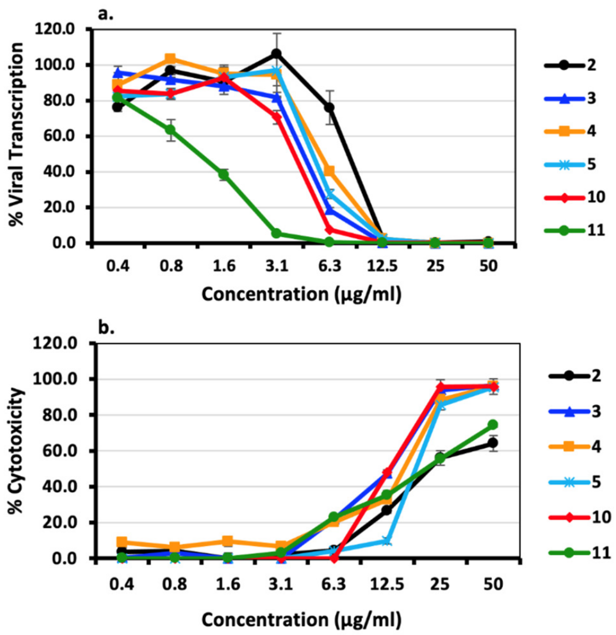

Figure S61. Antiviral activity and cytotoxicity of selected compounds. A549 Cells were infected with rA2-Rluc for 1h (a) or left uninfected (b) then treated in triplicate with serially diluted purified compounds. 24h post-infection, antiviral activity was determined by Renilla luciferase assay (a) and cytotoxicity was measured by MTT assay (b). Values are normalized to DMSO-treated cells. Shown are means ± SEM.

Table S12. IC_{50} values of isolated compounds against RSV.

Compound	EC_{50} (μM)
Neosuberitenone A (**1**)	>50
Suberitenone E (**2**)	20.5
Suberitenone F (**3**)	9.8
Suberitenone G (**4**)	11.0
Suberitenone H (**5**)	10.9
Suberitenone A (**9**)	7.9
Suberitenone B (**10**)	3.5
